# Red deer in Iberia: Molecular ecological studies in a southern refugium and inferences on European postglacial colonization history

**DOI:** 10.1371/journal.pone.0210282

**Published:** 2019-01-08

**Authors:** João Queirós, Pelayo Acevedo, João P. V. Santos, Jose Barasona, Beatriz Beltran-Beck, David González-Barrio, Jose A. Armenteros, Iratxe Diez-Delgado, Mariana Boadella, Isabel Fernandéz de Mera, Jose F. Ruiz-Fons, Joaquin Vicente, Jose de la Fuente, Christian Gortázar, Jeremy B. Searle, Paulo C. Alves

**Affiliations:** 1 Centro de Investigacão em Biodiversidade e Recursos Genéticos (CIBIO)/InBio Laboratório Associado, Universidade do Porto, R. Monte-Crasto, Vairão, Portugal; 2 Departamento de Biologia, Faculdade de Ciências da Universidade do Porto (FCUP), Porto, Portugal; 3 SaBio Research Group, Instituto de Investigación en Recursos Cinegéticos IREC (CSIC-UCLM-JCCM), Ronda de Toledo s/n, Ciudad Real, Spain; 4 Departamento de Biologia & CESAM, Universidade de Aveiro, Aveiro, Portugal; 5 SABIOtec. Ed. Polivalente UCLM, Ciudad Real, Spain; 6 Department of Veterinary Pathobiology, Center for Veterinary Health Sciences, Oklahoma State University, Stillwater, OK, United States of America; 7 Department of Ecology and Evolutionary Biology, Cornell University, Ithaca, NY, United States of America; 8 Wildlife Biology Program, University of Montana, Missoula, MT, United States of America; National Cheng Kung University, TAIWAN

## Abstract

The red deer (*Cervus elaphus*) is a widespread wild ungulate in Europe that has suffered strong anthropogenic impacts over their distribution during the last centuries, but also at the present time, due its economic importance as a game species. Here we focus on the evolutionary history of the red deer in Iberia, one of the three main southern refugial areas for temperate species in Europe, and addressed the hypothesis of a cryptic refugia at higher latitudes during the Last Glacial Maximum (LGM). A total of 911 individuals were sampled, genotyped for 34 microsatellites specifically developed for red deer and sequenced for a fragment of 670 bp of the mitochondrial (mtDNA) D-loop. The results were combined with published mtDNA sequences, and integrated with species distribution models and historical European paleo-distribution data, in order to further examine the alternative glacial refugial models and the influence of cryptic refugia on European postglacial colonization history. Clear genetic differentiation between Iberian and European contemporary populations was observed at nuclear and mtDNA levels, despite the mtDNA haplotypes central to the phylogenetic network are present across western Europe (including Iberia) suggesting a panmictic population in the past. Species distribution models, fossil records and genetic data support a timing of divergence between Iberian and European populations that overlap with the LGM. A notable population structure was also found within the Iberian Peninsula, although several populations displayed high levels of admixture as a consequence of recent red deer translocations. Five D-loop sub-lineages were found in Iberia that belong to the Western European mtDNA lineage, while there were four main clusters based on analysis of nuclear markers. Regarding glacial refugial models, our findings provide detailed support for the hypothesis that red deer may have persisted in cryptic northern refugia in western Europe during the LGM, most likely in southern France, southern Ireland, or in a region between them (continental shelf), and these regions were the source of individuals during the European re-colonization. This evidence heightens the importance of conserving the high mitochondrial and nuclear diversity currently observed in Iberian populations.

## Introduction

Understanding demographic and evolutionary history is fundamental to explain current species distributions and to forecast future trends under global climate change [1,2]. It has long been recognized that the Pleistocene glacial cycles caused massive fluctuations in the distributions of temperate species in Europe. During glaciations, central and northern European populations became extinct, with re-colonization from southern refugia during warm periods [3,4]. This demographic scenario with expansion-contraction involving southern refugia is supported by numerous species, however there is increasing evidence that northern cryptic refugia may have existed for a broad range of taxa and could have played an important role in the postglacial re-colonization at high latitudes in Europe [5–12]. The traditional southern refugial areas of the Mediterranean peninsulas may therefore either be important as source areas for the whole European distributions of widespread species or they may be sites of endemism, with central and northern Europe populated from previously unrecognized high latitude refugia [13,14].

The red deer (*Cervus elaphus*) is a good model species to address the hypothesis of cryptic refugia and its influence on the European postglacial colonization history, owing to its current and past widespread natural distribution across Europe [15,16], and known phylogeographical structure [17]. The most comprehensive study on a large biogeographical scale, using mitochondrial (mtDNA) *cytochrome b* data, has revealed that red deer originated in Asia and evolved into two distinct groups: a Western group with four lineages (Western Europe, Balkans, Africa and Middle East); and an Eastern group consisting of three lineages (North Asia/America, South-Asia and East-Asia) [18]. A more detailed analysis of the Western group throughout Europe and North Africa using mtDNA D-loop confirmed three of the four lineages previously reported: Western European lineage or haplogroup A, mainly distributed in Western Europe; Eastern European lineage or haplogroup C, distributed in Balkans (Eastern Europe); and Mediterranean lineage or haplogroup B, distributed in Africa and the Italian islands of Sardinia and Corsica. The estimated divergence time between these three lineages was around 300 thousand years before present (ky BP) [19], much earlier than the Last Glacial Maximum (LGM). However, the evolutionary rate used in Skog et al. [19] is based on a between-species fossil calibration [20] while, due to the time dependency of evolutionary rates [21], it is preferable to apply within-species calibrations for phylogeographic studies by incorporating ancient DNA sequences or population expansions attributable to well-dated geophysical events [22–24]. In addition, the dated red deer fossil records found outside the southern European Mediterranean peninsulas within the LGM interval [25] together with DNA analyses of some of these fossils [15,26] are consistent with the existence of one or more northern European refugium/refugia for this species. Therefore, northern cryptic refugia is a reasonable possibility for the red deer, and this hypothesis needs to be addressed and contrasted with the traditional model of southern (Mediterranean) refugia.

Iberia, one of the three main Mediterranean peninsulas (Iberian, Italian and Balkan), was an important southern refugial area for a wide range of temperate species [27] and also a suitable location for large cold-adapted mammals during the coldest periods [28]. Iberia is connected to central Europe via the Pyrenean mountain range, which has been considered an important barrier to gene flow between southern and northern populations during the Pleistocene [29–31]. Due to its physiographic complexity, multiple glacial refugia for various animal and plant species have been described in Iberia, supporting the ‘refugia within refugia’ model [27,32]. In particular, the demographic and evolutionary history of the red deer in Iberia and its relation with western European populations has been studied substantially over the last years, although the majority of wide-ranging phylogeographical studies are based in a limited number of populations and/or molecular markers [15,18–20,25,33–37]. Recent studies focused on Iberia using ancient [37] and contemporary [36] Iberian red deer samples have revealed a complex phylogeographical history. The analysis of ancient red deer specimens detected Western and Eastern European D-loop haplotypes in northwestern Spain during pre-LGM times [37]. Moreover, analysis of contemporary samples by Carranza et al. [33] revealed two mtDNA D-loop sub-lineages within the Western European lineage, and the authors argued that one of them contributed to the northern postglacial re-colonization of western Europe even though nuclear data were not adequate in this respect. Here we extend substantially on those studies, by employing a multidisciplinary approach involving mtDNA and nuclear data, species distribution models and fossil evidence in populations across Europe. This approach allows an unprecedented consideration of the LGM refugia of the red deer as well as the role of cryptic refugia in western Europe.

Since the Pleistocene, and especially during the 20th century, the red deer distribution has been subjected to direct (e.g. hunting pressure, translocations, introductions) and indirect (e.g. habitat fragmentation) anthropogenic influences [26,38]. In the Iberian Peninsula, both Portuguese and Spanish red deer populations suffered a steep decline in the middle of the last century as a consequence of habitat fragmentation and overexploitation [39]. Red deer distribution and density reached a minimum during that period, but a demographic expansion has been observed over the last decades, mainly promoted by humans [40,41]. Despite these recent anthropogenic impacts, the demographic history of Iberian populations is still marked by past natural biogeographical events [36,42]. However, in order to account for the recent human-induced effects, and discriminate them from the past demographic history, it is essential to use fast-evolving molecular markers [43]. We combined nuclear (34 microsatellite loci specifically developed for red deer) and mitochondrial (D-loop sequences) markers, together with species distribution modeling and paleo-distribution data, to elucidate red deer evolutionary history in Iberia and then address the hypothesis of a cryptic refugium at higher latitudes in Europe during the LGM.

## Materials and methods

### Ethics statement

All animal sampling took place post-mortem. Samples were obtained from harvested individuals during control programs or hunting events, independent of our research. According to EU and National legislation (2010/63/UE Directive and Spanish Royal Decree (53/2013) and to the University of Castilla–La Mancha guidelines, no permission or consent is required to conduct the research reported herein.

### Sampling

**Fig 1:**
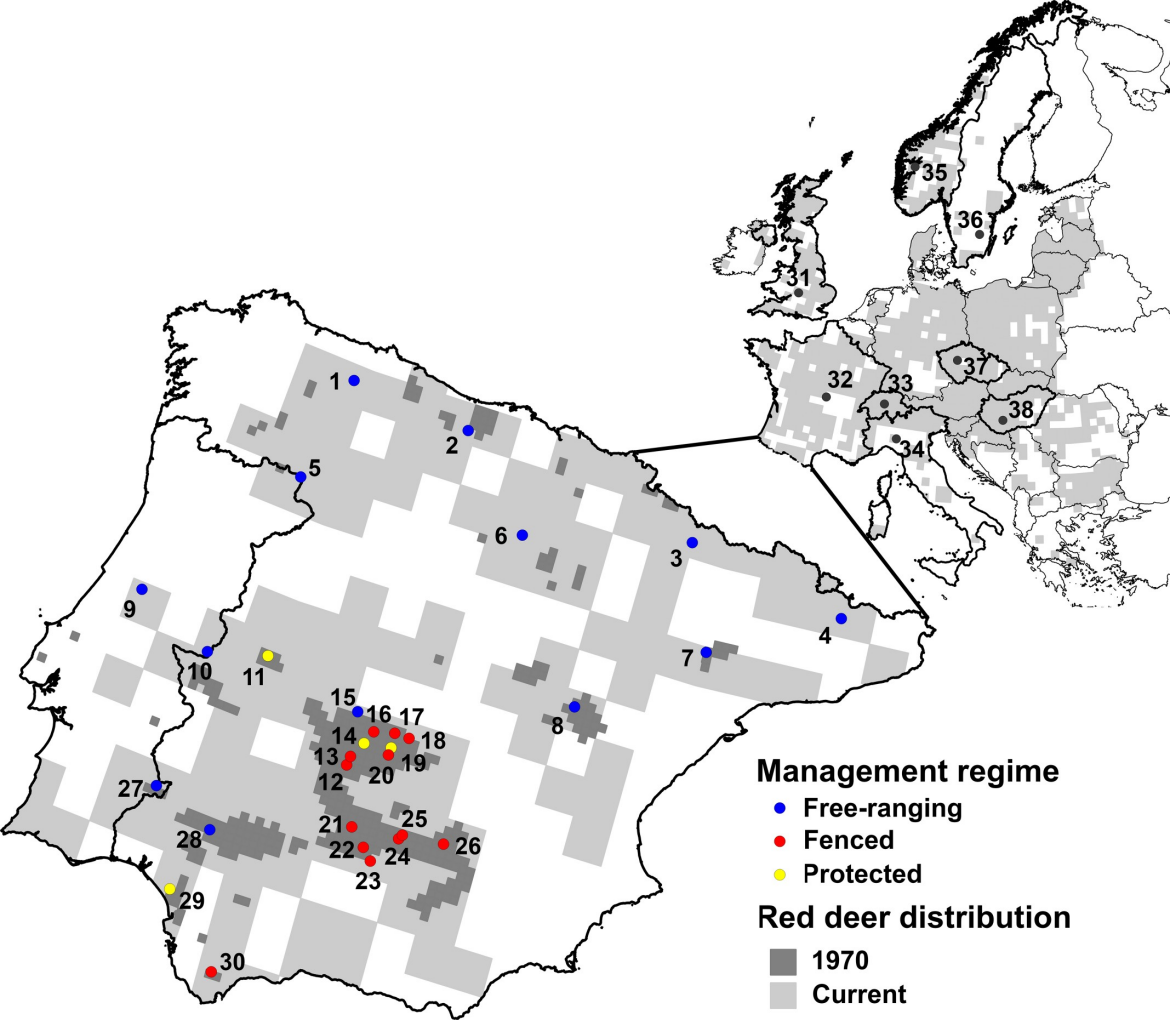
Location and management regime (within the Iberian Peninsula) of red deer populations sampled in this study (population code in brackets): 1-*Asturias* (ASR); 2-*Cantabria* (CTR); 3-*Huesca* (HUR); 4-*El Berguedà* (BER); 5-*Parque Natural de Montesinho/Sierra Culebra* (PMC); 6-*Burgos* (BUR); 7-*Caspe-Fraga* (CFR); 8-*Parque Natural do Alto Tajo* (PNA); 9-*Serra da Lousa* (SLR); 10-*Parque Natural do Tejo Internacional* (PNB); 11-*Parque Nacional de Monfragüe* (PNM); 12/20-*Montes de Toledo* region (MTR): 12-MT1, 13-MT2, 14-*Parque Nacional Cabañeros* (PNC), 15-MT3, 16-MT4, 17-MT5, 18-MT6, 19-*Quintos de Mora* estate (QMS) and 20-MT7; 21/26-*Sierra Morena* region (SMR): 21-SM1, 22-SM2, 23-SM3, 24-SM4, 25-SM5 and 26-SM6; 27-*Moura/Barrancos* (MBR); 28-*Parque Natural Sierra de Aracena y Picos de Aroche* (PNP); 29-*Parque Nacional Doñana* (PND); 30-*Parque Natural de Sierra de Grazalema* (PNS); 31-England (EN); 32-France (FR); 33-Switzerland (SW); 34-Italy (IT); 35-Norway (NO); 36-Sweden (SE); 37-Czech Republic (CZ); and 38-Hungary (HU). The red deer distribution range in western Europe used for modelling purposes is shown in light grey [16], while the Iberian red deer distribution around 1970 is represented in dark grey [39].

Sampling was conducted across Europe (n = 38 populations), with a particular focus on the Iberian Peninsula (n = 30). The sampling sites comprised a series of landholdings dispersed across the entire distribution range of the red deer (**Fig 1**). Most of the populations sampled are located in (or near) the regions where the red deer has been present since the 1970s, when there was still a reasonably natural distribution pattern [39]. These regions harbor populations under fenced, protected and free-ranging management regimes (**Fig 1**) [42]. Red deer populations sampled from central and northern Europe [England (EN), France (FR), Switzerland (SW), Italy (IT), Norway (NO), Sweden (SE), Czech Republic (CZ) and Hungary (HU)] were all from free-ranging populations, although hunted. The tissue samples consisted of a portion of the spleen taken from individuals hunted during the regular hunting season or during targeted control programs (between 2005/2006 and 2014/2015—October to February). In total, 911 samples were collected.

### Molecular procedures

Genomic DNA was extracted using the EasySpin Genomic DNA Tissue Minipreps Kit according to the manufacturer’s instructions. A set of 35 autosomal microsatellite markers previously developed for red deer was used to determine individual multilocus genotypes [44]. Primer sequences, details of multiplex reactions and amplification procedures follow [44]. Multiplex PCR products were run on an ABI3100xl genetic analyzer together with the 400 LIS^TM^ size standard. Fragment analysis was conducted using the software GENEMAPPER 4.0 (Applied Biosystems) and checked manually by two researchers independently. In order to thoroughly verify and confirm the genotypes observed, 10% of the samples were replicated and reanalyzed to check allelic compatibility. Furthermore, MICRO-CHECKER 2.2.3 was used to discard null alleles, scoring errors and allelic dropout [45]. Deviations from Hardy-Weinberg equilibrium and linkage equilibrium between loci were tested for each red deer population using Fisher’s exact tests implemented in GENEPOP 4.0.10 [46]. Significance levels estimated by Markov Chain Analysis (10^4^ dememorization steps, 10^3^ batches and 10^4^ iterations per batch) were adjusted using Bonferroni’s sequential method for multiple comparisons [47]. All microsatellites are in Hardy-Weinberg equilibrium within each population except at locus Ceh33 in CFR, Ceh66 in MBR, Ceh45 in MT2 and MT6, Ceh43 and Ceh44 in SW, Ceh50 and Ceh53 in FR, Ceh55 in SM4 and Ceh73 in PNS. All markers are in linkage equilibrium. The Ceh78 locus was excluded from the final dataset due to the absence of polymorphism in the majority of the populations.

A mtDNA fragment covering the hyper-variable regions HVR1 and part of HVR2 of the D-loop (670 bp) was amplified using the primer pair LD5 and HD6, following the PCR conditions reported by Nagata el al. [48]. Successful amplifications were purified using the enzymes exonuclease I and shrimp alkaline phosphatase, and then sequenced with BigDye chemistry (Applied Biosystems), mainly using the HD6 primer and following the BigDye Terminator v3.1 cycle sequencing protocol (Applied Biosystems). Electropherograms were checked and aligned using SEQSCAPE 2.5 (Applied Biosystems). Samples that produced singleton haplotypes or haplotypes that differ in only one base pair from a common haplotype were re-amplified and re-sequenced with forward and reverse primers in order to confirm the presence of true haplotypes.

### Data analysis

#### Nuclear genetic diversity and population structure

Basic population genetic diversity parameters, number of alleles (*Na*); private alleles (*PA*); and observed (*H*_*o*_) and expected (*H*_*e*_) heterozygosities, were calculated for autosomal microsatellite markers using GENEALEX 6.5 [49]. The allelic richness (*AR*) and *F*-statistics were estimated using FSTAT 2.9.3.2 [50]. Signatures of genetic bottlenecks were assessed using the heterozygosity excess test implemented in BOTTLENECK 1.2.02 [51]. A two-phase mutation model [52] was used with the default settings of 30% of variation from the infinite allele model and of 70% from the stepwise-mutation-model. Heterozygous excess at all loci was tested with a one-tailed Wilcoxon sign rank test (10^4^ iterations) [53].

The degree of genetic differentiation between populations was quantified using *F*-statistics [50,54] and isolation-by-distance within the Iberian Peninsula was tested by a Mantel test [55] (using the pairwise *F*_*ST*_ values). The Bayesian cluster method implemented in STRUCTURE 2.3.4 [56] and the factorial correspondence analysis (FCA) implemented in GENETIX 4.05 [57] were used to assess the levels of population structure. A hierarchical structure analysis was performed using different datasets: 1) all populations studied (Iberia plus central and northern Europe); 2) only the Iberian populations and the individuals assigned to the Iberian cluster (>95% membership proportion) in the previous analysis. In this dataset, we also excluded the specimens that had haplotype 11, since it might have evolved outside the Iberian Peninsula [33]; and 3) only the central and northern European populations. Default STRUCTURE parameters were set together with an admixture model in combination with correlated allele frequencies [58] and no prior-information about population origin. The log likelihood of the data ln (P(*X*|*K*)) was calculated for *K* = 1 to *K* = 17 in the first and second run, and *K* = 1 to *K* = 9 in the third run, with 10 repetitions of 10^6^ MCMC iterations following a burn-in period of 10^5^ steps. Moreover, Δ*K* was calculated following the procedures described in [59], and using STRUCTURE HARVESTER 0.6.94 [60]. Since the best Δ*K* value inferred from global analysis allowed us to distinguish Iberian from other European populations, it was then used to quantify the degree of genetic introgresion from Europe into the Iberian populations.

#### Mitochondrial genetic diversity and phylogeography

Mitochondrial diversity was evaluated based on the number of private haplotypes, haplotype diversity (*h*) [61] and nucleotide diversity (π;) [62] using DnaSP 5.10.1 [63]. Additionally, two neutrality test statistics, Tajima’s (1989) *D* [64] and Fu’s (1997) *F*_*S*_ [65], were calculated to detect signs of population expansion or selection using ARLEQUIN 3.5.1.2 [66]. Departures from a neutral model were tested with 10^4^ coalescent simulations of the genealogy. The degree of genetic differentiation between populations was quantified using *F*-statistics [54,66] and isolation-by-distance within the Iberian Peninsula tested by a Mantel test [55] (using the pairwise *F*_*ST*_ values).

The new D-loop sequences (670 bp) were aligned together with 624 sequences retrieved from GenBank, comprising the representative haplotypes from the majority of red deer studies published throughout Europe and North Africa up to 2017 [13,17,24,30,31,34,64–82]. Phylogeographic analyses were performed at two geographical scales: the central and northern European level and the Iberian level. At the central and northern European level, two datasets covering distinct D-loop fragment sizes were used: i) a 329 bp D-loop fragment (HVR1), which comprised our sequences and those available in GenBank (see S1 Table); ii) a 670 bp D-loop fragment (HVR1 and partially HVR2), which just included the sequences amplified in this study across Europe. A median-joining network [83] was built using NETWORK 4.6 (Fluxus Technology Ltd) for both datasets in order to assess the level of homoplasy between distinct mtDNA fragment lengths. Among all the available sequences in GenBank, the sequences published by Niedziałkowska et al. [84] (n = 28), Meiri et al. [15] (n = 59), Rey-Iglesia et al. [37] (n = 14) and Stanton et al. [26] (n = 14) were not included in the former analysis (i) due to their small and non-overlapping fragment size. Nevertheless, the similarity between those sequences (248 bp, 180 bp, 449 bp and 264 bp, respectively) and our data (ii) was assessed and can be consulted in the S2, S3, S4 and S5 Tables, respectively.

At the Iberian Peninsula level, two datasets covering distinct D-loop fragment sizes were used: i) a 650 bp D-loop fragment, which included our Iberian sequences and those described in Iberian contemporary populations by Carranza et al. [36]; ii) a 670 bp D-loop fragment, which just comprise the Iberian sequences amplified in this study. Networks were constructed following the same procedures described above for the European level.

### Mitochondrial D-Loop evolutionary rate: Within-species calibration model

The evolutionary rate for the D-loop fragment was estimated using a within-species calibration model. The substitution rate of five ancient DNA sequences (dispersed throughout Europe) directly dated by Meiri et al. [15] (see S6 Table) were used together with the contemporary haplotypes described in this study. These analyses were carried out in BEAST 1.8.2 [85] using the HKY+I+G substitution model, which was selected based on the heuristic algorithm implemented in the jModelTest2 2.1.4 [86], and a strict and an uncorrelated lognormal-distributed relaxed clock, assuming a constant population size coalescent as the tree priors. For each model, two independent runs were performed using 3x10^7^ Markov Chain Monte Carlo (MCMC) iterations preceded by 3x10^6^ steps of burn-in, with model parameters being sampled every 3x10^4^ steps. For each model, both independent runs were combined resulting in 54x10^6^ iterations. Using TRACER 1.5 [87], the final model was chosen based on a Log_10_ of the Bayes factor (BF) being more than 1.3. The best-fit comparison of the results of the Bayesian MCMC analysis, calibrated with fossil age data, favors the relaxed clock model over the strict clock model (Log_10_ BF = 2.92 >1.3; see S7 Table). Using the constant size model and a relaxed clock we estimated a substitution rate of 3.849x10^-7^ substitutions per site per year (CI_95%_ 4.398x10^-8^ to 8.301x10^-7^).

### Population demography and divergence

To assess the population demography and estimate the levels of divergence among red deer populations, we used both the mitochondrial and microsatellite data obtained in this study. Microsatellite data and the Approximate Bayesian Computation (ABC) analysis implemented on DIYABC 2.0 [88] was used to test scenarios of divergence among populations and estimate timing of divergence and effective population sizes of each population. Mitochondrial data and the Bayesian Skyline Plot methodology implemented on BEAST 1.8.2 [85] was used to explore the past demographic changes of red deer populations and the time of divergence among mitochondrial lineages.

**Fig 2:**
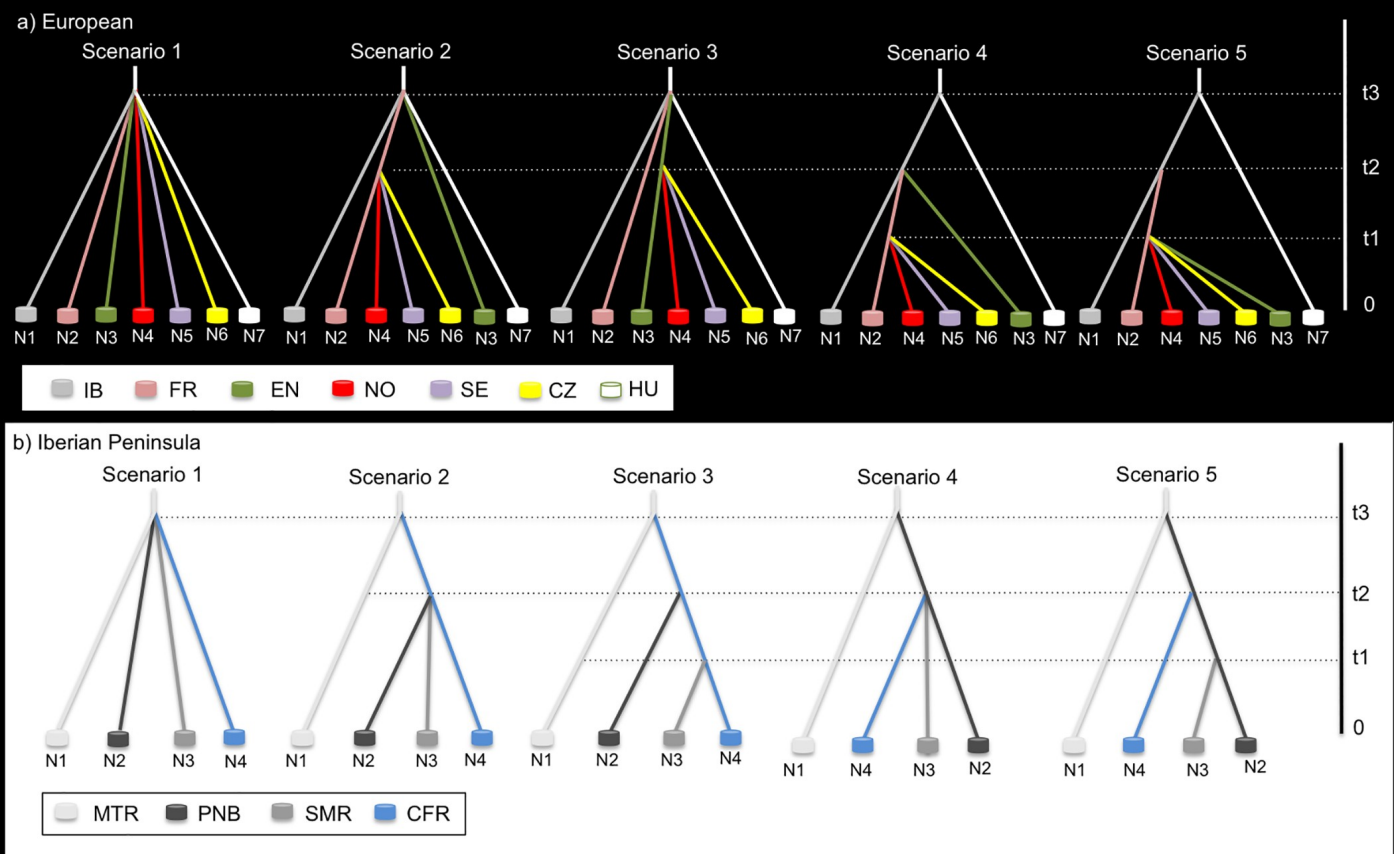
Putative scenarios of divergence (for 3 different times: t1, t2 and t3) among (a) European red deer populations (Iberian—IB, French—FR, English–EN, Norwegian–NO, Swedish–SE, Czech–CZ and Hungarian–HU) and (b) the Iberian populations (Montes de Toledo region–MTR, Parque Natural de Tejo Internacional–PNB, Sierra Morena region–SMR and Caspe/Fraga region–CFR;) tested using Approximate Bayesian Computation (ABC) analyses implemented in DIYABC 2.0. Details about the individuals included on the Iberian populations are depicted in the Material and methods section.

An ABC [89,90] statistical framework was employed to compare plausible scenarios of divergence among red deer populations across Europe (Iberia plus central and northern Europe; five scenarios) and within the Iberian Peninsula (five scenarios). The time of divergence and effective population sizes of each population was also estimated. Scenarios were created on the basis of the most plausible refugium, and subsequent post-glacial expansion of populations, that could be inferred from the results of molecular, paleontological and species distribution models obtained in this study (see below), together with previous evidence reported in other studies (e.g. [5–12]). To reduce the computational demands and avoid potentially confounding effects of contemporary hybridization we only included individuals with membership proportion higher than 95% to their respective population in STRUCTURE analyses. In the case of analyses at the European level, the Italian and Swiss populations were excluded due to the admixture pattern inferred. The first scenario tested a simultaneous divergence of all European populations at t3. The second scenario predicts that the Iberian (IB), English (EN), French (FR) and Hungarian (HU) populations diverged simultaneously at t3, but the Norwegian (NO), Swedish (SE) and Czech (CZ) populations diverged at t2 from FR. The third scenario is similar to the previous one but predicts that NO, SE and CZ diverged at t2 from EN. The fourth scenario predicts that IB and HU diverged at t3, and afterwards FR and EN diverged from IB at t2 and NO, SE and CZ diverged from FR at t1. The fifth scenario is similar to scenario 4, but in this case, predicts that EN population diverged at t1 from FR (**Fig 2A**).

Scenarios of divergence were also constructed considering only the Iberian populations (**Fig 2B**). To reduce the computational demands and avoid potentially confounding effects of contemporary hybridization, random individuals from the most preserved populations were selected: individuals from PNC (n = 16), QMS (n = 31), PNA (n = 13), PND (n = 28), SM2 (n = 11), PNB (n = 14) and CFR (n = 49). The first scenario consisted of a simultaneous divergence of populations at t3. The second scenario predicts that the Caspe/Fraga region (CFR) populations diverged from a putative refugium in Montes de Toledo (MTR, a cluster that harbors mtDNA haplotypes central to the phylogenetic network) at t3, and afterwards the Sierra Morena (SMR) and Parque Natural de Tejo Internacional (PNB) populations diverged simultaneously at t2 from CFR. The third scenario is similar to the previous one but predicts that SMR diverged at t1 from CFR. The fourth scenario predicts that PNB diverged at t3 from a putative refugium in MTR, and afterwards CFR and SMR diverged from PNB at t2. The fifth scenario is similar to scenario 4, but in this case, predicts that SMR population diverged at t1 from PNB (**Fig 2B**).

Simulations for each scenario and ABC analyses were carried out using DIYABC 2.0 [88]. One million simulated datasets per scenario were generated with a 1:1 female to male sex ratio, a generalized stepwise-mutation model and uniform priors with default values for all parameters. We considered the following constraints on temporal parameters: t3>t2, t3>t1 and t2>t1 for all populations. Amongst the recommended summary statistics [88] we selected five that have been commonly used to infer population divergence and admixture [91,92]: i) mean number of alleles; ii) mean genic diversity, ii) mean allelic size variance, iv) Fst, v) classification index. The posterior probability of scenarios was assessed using a polychotomic weighted logistic regression on the 1% of simulated datasets closest to the observed data [88]. The confidence in scenario choice was assessed (Type I and Type II error rates) using 500 pseudo-observed datasets simulated under each scenario. For the best supported scenario, the posterior distribution of all parameters was estimated using local linear regressions on the 1% of the simulations closest to the observed data after a logit transformation of parameter values [89,93]. The performance of parameter estimation was assessed by calculating the median of the relative median of absolute errors from 500 pseudo-observed datasets [94]. Finally, the goodness of fit of the best supported scenario was evaluated by simulating 10 000 pseudo-observed datasets from the posterior distribution of all parameters. Summary statistics parameters different from which had been used for model selection or parameter estimation in previous ABC analyses were applied [94]. We used a principal component analysis on test statistic vectors to visualize the fit between simulated and observed datasets, and ranking summary statistics to assess whether the best supported model can successfully reproduce observed data (i.e. % of simulated data < observed data).

A Bayesian Skyline Plot was constructed for the Iberian red deer populations using the D-loop fragment (670 bp), the estimated evolutionary rate and the model of nucleotide evolution (HKY+I+G) under both a strict and a relaxed molecular clock and assuming a constant population size coalescent as the tree priors. For each model, two independent runs were performed using 3x10^7^ Markov Chain Monte Carlo (MCMC) iterations preceded by 3x10^6^ steps of burn-in, with model parameters being sampled every 3x10^4^ steps. For each model, both independent runs were combined resulting in 54x10^6^ iterations. The best-fit comparison of MCMC favors, in this case, a strict clock over the relaxed molecular clock (Log_10_ BF = 200 >1.3). Thus, the strict clock model was used for the final runs. Additionally, because of the high number of haplotypes described in the British Isles, the sequences published by Pérez-Espona et al. [71] and McDevitt et al. [68] were used to explore the past demographic changes of the red deer populations in that region, as described for the Iberian Peninsula. Using a within-species calibration rate (3.849x10^-7^, determined with our data), we also calculated the divergence time among the Western European lineage (haplogroup A), the Eastern European lineage (haplogroup C) and the Mediterranean lineage (haplogroup B) [19] using the D-loop 329 bp fragment, and the divergence time between the private evolutionary sub-lineages found in Iberia and the mtDNA haplotypes central to the phylogenetic network using the 670 bp fragment.

### Species distribution modelling

The distribution range of the red deer throughout Europe and North Africa was obtained on UTM 50 km × 50 km grid squares from [16] and [95], respectively. Using these data and an inductive approach based on current bioclimatic conditions, the climatic requirements of the species were determined, i.e., the climatic niche of the species. Current and past bioclimatic variables were downloaded from WorldClim 1.4: present (CCSM4 at 2.5 arc-minutes resolution) [96], 6 ky BP (CCSM4 at 2.5 arc-minutes resolution) and 22 ky BP (CCSM4 at 2.5 arc-minutes resolution) [97], and 120 ky BP (30 arc-seconds resolution) [98]. Model parameterization was conducted using an 80% random sample of the dataset and the model predictive performance was evaluated against the remaining 20% of the data (validation dataset). In order to avoid multicollinearity among predictors (see S8 Table), which can bias the model predictions when transferred outside the time period where it was trained, their collinearity was quantified using the variance inflation factor (VIF) following [99]. The covariate with the highest VIF was sequentially dropped, the VIF were recalculated, and this process was repeated until all variables achieved VIF no higher than 10 [100]. Finally, seven variables (BIO1, BIO2, BIO4, BIO8, BIO14, BIO15, BIO19) were selected for modelling purposes. Generalized linear models (GLM) were fitted for the presence/absence of red deer using non-collinear variables [99], a logit link function and a binomial error (S9 Table). Two components of the model’s predictive performance were assessed on the validation dataset, namely discrimination (area under the ROC curve) and reliability (calibration plot and associated statistics) [101].

The model was then hindcasted in order to integrate distributional data with genetic and paleontological evidence. The species’ climatic requirements were assumed to have remained stable over time to infer suitable areas for red deer in the past. This was carried out through replacing the bioclimatic variables selected in the final model by those estimated for the past scenarios. However, the environmental domain of the model in the past should be assessed before transference, since the model is only able to work in the environmental domain in which the model was parameterized. To this end, the methodology proposed by [102] was applied, i.e. the multivariate environment similarity surfaces analysis (MESS), which measures the similarity of any given locality (in past scenarios) to a reference set of points (climatic conditions at present), with respect to the predictor variables chosen. The MESS applied to the past scenarios allowed us to identify the sites where at least one variable had a value that is outside the range shown by the reference set. Only predictions for those territories identified by MESS as within the climatic domain of the model were used for integration with genetic and paleontological data.

In addition to GLM predictions and following the same analytical procedure, several different modelling techniques widely used in species distribution modelling were also applied [namely generalized additive model (GAM), generalized boosting model (GBM), classification tree analysis (CTA), artificial neural network (ANN), BIOCLIM and flexible discriminant analysis (FDA), and an ensemble of their forecasts] to assess the consistency of the predictions obtained from GLM. Analyses were carried out with biomod2 using default specifications for each technique (for further details see [103]). Results showed that GBM and CTA performed adequately according to discrimination measures, while GAM, ANN, SRE and FDA did not achieve the threshold of true kill statistic (TSS) > 0.6 and relative operating characteristic (ROC) > 0.7. Only techniques with TSS > 0.6 and ROC > 0.7 were considered for the ensemble and the predictive performance of the ensemble was similar to that achieved by other techniques (see S10 Table). Overall, logistic regression modelling provided consistent results for the Mid-Holocene, the LGM and the interglacial period when compared with those obtained for GBM, CTA and the ensemble. As the dimensionality of the models [104] and their complexity [105] limit their capability to be projected, in our study we opted to show results from logistic regression as a simpler, well-known technique able to produce robust inference [106]. All statistical analyses were carried out in R 2.15.2 [107].

### Archaeological/paleontological data recompilation

Published red deer fossil records were recompiled, mainly from [25] and [15], to address the paleo-distribution of the red deer throughout the archaeological/paleontological sites in Europe during the Pleistocene. Radiocarbon dates were then calibrated in OXCAL 4.2 (https://c14.arch.ox.ac.uk/oxcal/OxCal.html) using the calibration curve Int-Cal13 [108] and the results are shown as median calibrated dates (with 95% confidence intervals). The dates were further organized according to the favorable areas predicted by the 22 ky BP species distribution model. Thus, the dates were divided in nine groups: central-north Europe (CN-EU), east Europe (E-EU), south-west France (SW-FR), south-east France (SE-FR), British Isles (BI), Italy (IT), west Iberian Peninsula (W-IB), north Iberian Peninsula (N-IB) and south-east Iberian Peninsula (SE-IB).

## Results

### Nuclear genetic diversity and population structure

An overall south-north pattern of decreasing nuclear diversity was observed among European populations, although a wide range of values was obtained, including in the Iberian Peninsula where there were signs of genetic bottlenecks and inbreeding for several populations (**Table 1**).

**Table 1:** Population genetic diversity parameters of the red deer populations calculated using 34 microsatellites specifically developed for this species and mitochondrial D-loop fragment (670 bp). Population codes are described as in **Fig 1**.

Population		Autosomal microsatellites								Mitochondrial D-Loop fragment						
		*n*	*N*_*a*_	*AR*	*H*_*o*_	*H*_*e*_	PA	*F*_*IS*_	*TPM*	*n*	*N*_*h*_	*h*	*π*	*PH*	*Tajima's D*	*Fu's Fs*
**IP**	**ASR**	31	4.6	2.9	0.55	0.56	0	0.02	0.185	31	6	0.79	0.84	0	1.68	4.91
	**CTR**	37	4.4	2.8	0.51	0.53	0	0.04	0.038*	36	4	0.74	0.78	0	2.49	8.32
	**HUR**	10	3.4	2.6	0.44	0.46	0	0.11	0.664	6	2	0.33	0.10	0	-1.13	0.95
	**BER**	19	4.0	2.8	0.51	0.52	0	0.05	0.381	20	5	0.66	0.74	1	0.13	3.94
	**PMC**	12	3.7	2.8	0.51	0.51	0	0.03	0.368	12	3	0.53	0.49	0	0.92	3.85
	**BUR**	4	2.4	2.4	0.57	0.42	0	-0.08	0.082	4	2	0.50	0.15	0	-0.71	1.10
	**CFR**	56	3.4	2.4	0.43	0.44	1	0.11*	0.052	54	3	0.59	0.57	1	2.53	9.43
	**PNA**	38	4.4	2.8	0.54	0.54	0	0.00	0.026*	37	5	0.71	0.51	1	0.89	3.90
	**SLR**	26	4.3	2.8	0.52	0.52	2	0.00	0.520	26	4	0.61	0.48	1	0.34	4.09
	**PNB**	23	3.3	2.5	0.47	0.48	0	0.04	0.053	22	2	0.17	0.03	2	-0.64	-0.18
	**PNM**	25	3.7	2.6	0.45	0.47	0	0.08*	0.266	24	1	0.00	0.00	0	0.00	-1.03
	**MT1**	20	4.1	2.7	0.52	0.51	0	-0.02	0.639	21	5	0.72	0.52	0	0.13	2.55
	**MT2**	27	4.1	2.8	0.50	0.53	1	0.06	0.167	27	3	0.45	0.50	0	1.36	6.27
	**PNC**	24	4.4	2.9	0.53	0.53	1	0.02	0.223	22	5	0.65	0.63	1	1.39	3.52
	**MT3**	29	4.5	2.9	0.52	0.53	1	0.04	0.400	28	3	0.68	0.52	0	2.12	6.62
	**MT4**	34	4.4	2.8	0.52	0.53	1	0.04	0.095	34	4	0.68	0.44	0	1.04	4.39
	**MT5**	27	4.4	2.9	0.57	0.56	0	0.00	0.001*	27	5	0.66	0.33	2	0.19	1.47
	**MT6**	27	4.5	3.0	0.54	0.56	1	0.05	0.022*	27	4	0.49	0.31	0	-0.33	2.43
	**QMS**	44	4.5	2.9	0.57	0.56	0	0.00	0.011*	43	5	0.44	0.30	0	-0.65	1.84
	**MT7**	53	4.6	2.9	0.54	0.54	0	0.01	0.013*	53	4	0.75	0.57	0	2.51	7.22
	**SM1**	26	4.4	2.9	0.54	0.53	1	-0.02	0.277	25	4	0.68	0.43	0	-0.04	3.54
	**SM2**	21	4.0	2.8	0.54	0.54	0	0.05	0.004*	19	2	0.53	0.55	0	2.80	8.38
	**SM3**	25	4.1	2.8	0.50	0.51	1	0.06	0.054	24	5	0.73	0.54	0	1.19	3.09
	**SM4**	31	4.6	2.9	0.51	0.53	0	0.06*	0.324	28	7	0.78	0.57	0	1.52	1.55
	**SM5**	22	4.0	2.8	0.51	0.51	0	0.04	0.160	20	4	0.60	0.27	0	-1.00	1.48
	**SM6**	18	3.9	2.7	0.52	0.51	1	0.06	0.040*	19	3	0.29	0.22	1	-1.22	2.17
	**MBR**	16	3.4	2.6	0.49	0.47	0	0.13	0.013*	15	2	0.42	0.69	0	1.40	9.40
	**PNP**	23	4.6	2.9	0.51	0.52	0	0.03	0.426	25	9	0.87	0.94	1	0.69	1.64
	**PND**	30	3.1	2.4	0.41	0.43	1	0.18*	0.014*	33	5	0.33	0.37	2	-1.07	2.26
	**PNS**	19	3.8	2.6	0.46	0.47	0	0.09*	0.465	17	3	0.58	0.70	1	1.18	6.63
**EU**	**EN**	17	3.2	2.5	0.52	0.47	1	-0.05	0.019*	17	7	0.83	1.55	4	-1.65*	4.25
	**FR**	33	4.5	3.0	0.52	0.51	1	0.02	0.263	31	2	0.18	<0.00	2	-0.43	4.15
	**SW**	23	4.9	2.9	0.50	0.53	5	0.12	0.797	23	7	0.81	0.02	3	0.54	8,08
	**SE**	5	2.0	2.0	0.30	0.30	1	0.37	0.384	5	1	0.00	<0.00	0	0.00	0.00
	**CZ**	10	4.2	3.2	0.57	0.56	3	0.06	0.479	10	3	0.73	0.01	2	1.77	4.44
	**HU**	10	4.0	3.0	0.53	0.53	4	0.12	0.170	10	2	0.20	<0.00	1	-1.84*	3.34
	**IT**	12	4.7	3.2	0.53	0.56	5	0.20	0.876	10	6	0.87	0.02	2	0.88	3.56
	**NO**	5	2.4	2.4	0.33	0.37	1	0.25	0.432	5	2	0.60	<0.00	2	1.64	3.02

*IP–*Iberian Peninsula; *EU–*Europe; *n*–number of samples included in each analysis; *N*_*a*_—number of alleles; *AR*—allelic richness; *H*_*o*_*/H*_*e*_ observed and expected heterozygosity; *PA*—private alleles; *F*_*IS*_—coefficient of inbreeding; *TPM*—bottleneck, *P-value* of heterozygosity excess test; *N*_*h*_—number of haplotypes; *h*—haplotype diversity; *π* - nucleotide diversity in %; *PH*—private haplotypes.

* *P* < 0.05.

**Fig 3:**
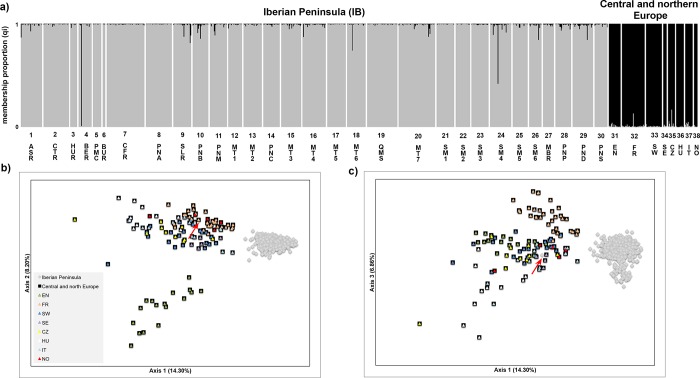
Population genetic structure of the Iberian and European red deer using microsatellite data. a) Bayesian clustering analyses conducted in STRUCTURE and considering the best Δ*K* value (Δ*K* = 2). Individual membership proportion to each cluster is indicated in grey for the Iberian populations and black for the central and northern European populations (see details S1 Fig); b) and c) Plots showing the results of the Factorial Correspondence Analysis–b) components 1 and 2, c) components 1 and 3. A red arrow highlights the individual identified (in BER population) as belonging to the European cluster. Population codes are described as in **Fig 1**.

Population genetic structure analyses clearly differentiated the Iberian from the other European populations. In STRUCTURE analysis (all populations), although the highest likelihood was achieved for *K* = 13, the maximized Δ*K* value was reached for *K =* 2, segregating the Iberian from the other European populations (**Fig 3A;** see also S1 Fig). This segregation was also found in FCA analysis (**3B and 3C**) and when the levels of genetic differentiation among populations measured as *F*_*ST*_ were assessed and represented by neighbor-joining trees (see S2 Fig and S11 Table). Therefore, considering two clusters as the best supported partition at European level, the Iberian populations studied showed relatively low levels of introgression from European populations, with the exception of the BER population where one individual was truly identified as belonging to the European cluster in both analyses (**Fig 3A, 3B and 3C**). However, other individuals in different Iberian populations depicted a membership proportion (*qi*) to European populations higher than 5% in STRUCTURE analysis. Although these individuals (n = 24) were allocated to the Iberian cluster in FCA, they were further excluded from STRUCTURE analysis at the Iberian level, together with one individual from BER and the specimens that harbored haplotype 11 (n = 50; see S12 Table). Since two individuals were excluded on the grounds of both the nuclear and the mitochondrial data, a total of 73 individuals were not included when analyzing the data at the Iberian level.

**Fig 4:**
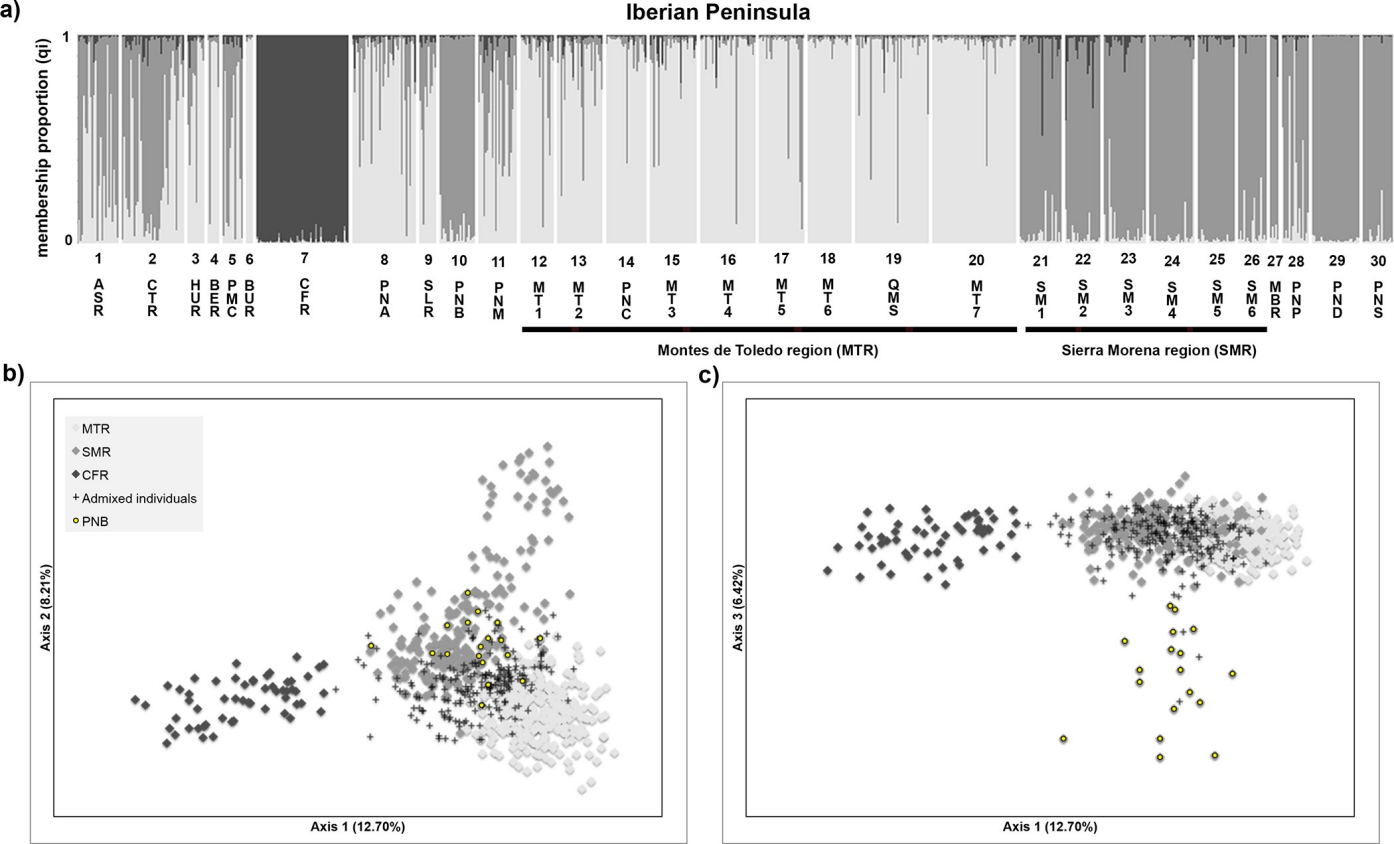
Population genetic structure in the Iberian red deer using microsatellite data. a) Bayesian clustering analyses conducted in STRUCTURE and considering the highest Δ*K* value (Δ*K* = 3). Individual membership proportion to each cluster is indicated in light grey for the Montes de Toledo cluster, grey for the Sierra Morena cluster and dark grey for the Fraga/Caspe cluster (see details S1 Fig); b) and c) Plots showing the results of the Factorial Correspondence Analysis. The population from Parque Natural Tejo Internacional (PNB) was distinctive as well as the three aforementioned clusters–b) components 1 and 2, c) components 1 and 3. Population codes are described as in **Fig 1**. These analyses excluded the individuals sampled in the Iberian Peninsula that showed a membership proportion lower than 95% for the Iberian cluster in the European analysis and those individuals that had the mtDNA haplotype 11 (n = 73, see details S12 Table).

Thus, at the level of the Iberian Peninsula, STRUCTURE analysis was conducted using 724 individuals. Although the highest likelihood was achieved for *K* = 11, the maximized Δ*K* value was reached for *K =* 3 (**Fig 4A;** see also S1 Fig), segregating populations from Caspe/Fraga region (CFR), Montes de Toledo region (MTR) and Sierra Morena region (SMR). However, most of the Iberian red deer populations, namely from northern Iberia, showed a high admixture pattern between the clusters MTR and SMR. FCA showed a similar genetic structure among the Iberian populations as STRUCTURE (**Fig 4B and 4C**). While individuals from the CFR cluster were clearly separated from the remaining populations, the sub-structuring of populations from the MTR and SMR clusters was not so evident due to great admixture observed. Additionally, FCA distinguished the PNB population from the SMR cluster. Among all the Iberian populations a moderate genetic differentiation was apparent, with a global *F*_*ST*_ of 7.7% (95% confidence limits: 6.9–8.5%). The maximum pairwise *F*_*ST*_ value observed was 23% (PND-CFR), with a positive significant correlation between autosomal genetic distances (*F*_*ST*_/(1-*F*_*ST*_)) and geographic distances (log km) in the Mantel test (r^2^ = 0.35, *P* < 0.001) (see S3 Fig).

Concerning the central and northern European populations, in the STRUCTURE analysis the highest likelihood was reached for *K* = 8, although very similar Δ*K* values (maximized) were achieved for *K =* 3 and *K* = 8 (see S1 and S4 Figs). While the English (EN) and French (FR) populations are separate from the remaining populations for *K* = 3, almost all analyzed populations form specific clusters when considering *K* = 8, with the exception of the Swiss (SW) population that segregates into two clusters and the Italian (IT) population that showed an admixture pattern between central and northern European populations. FCA analysis showed a consistent genetic structure relative to that obtained in the STRUCTURE analysis for *K* = 3 with EN and FR populations separated from the remaining populations (**Fig 3B and 3C**).

### Mitochondrial genetic diversity and phylogeography of the European red deer

**Fig 5:**
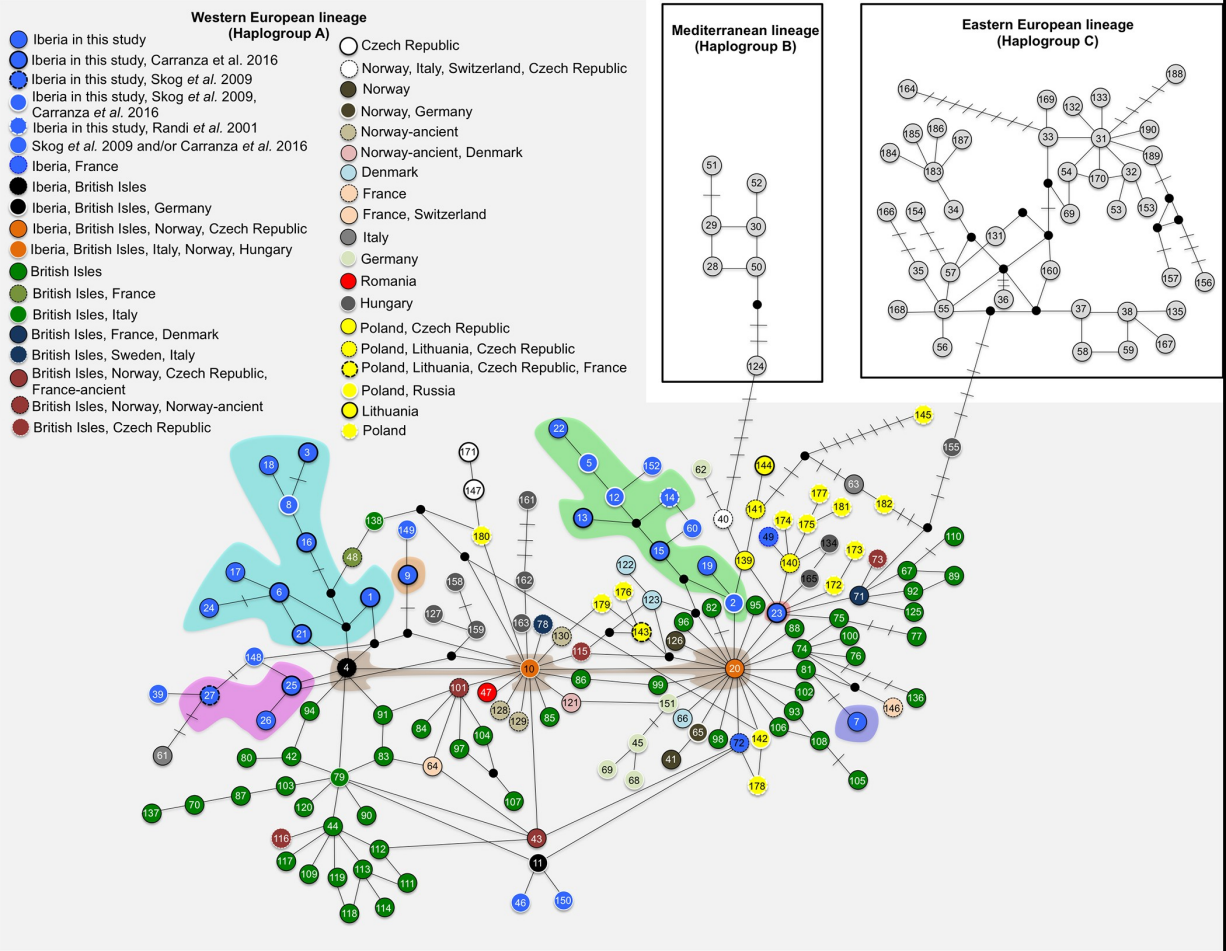
Median joining network showing the evolutionary relationship of mtDNA D-loop haplotypes found both in this study and those published in GenBank (329 bp fragment size) for the red deer in Europe. A bar in each solid line represents a mutational step; small black circles show undetected/extinct intermediate haplotype states; color codes within the circles are depicted for Western European lineage (haplogroup A) and represent the country where the haplotypes were found. The Iberian sub-lineages are grouped by colored shading following **Fig 6**. GenBank accession numbers of the 509 original haplotypes used in this analysis, as well as their correspondent haplotypes for 329 bp (represented by numbers within circles) are given in S1 Table.

A south-north pattern of decreasing mitochondrial diversity was inferred across Europe, although a wide range of values was found across European populations (**Table 1**). Signs of population expansion or selection were observed for the EN and HU populations (**Table 1**). Three main European mtDNA lineages can clearly be identified in the network using the 329 bp D-loop fragment (**Fig 5**; n = 190 haplotypes, see details in S1 Table). The Western European haplotypes (n = 144, haplogroup A) dispersed mainly across western Europe, but also in the center and northeast of the continent. The Eastern European haplotypes (n = 39, haplogroup C) occurred across eastern Europe, while the Mediterranean haplotypes (n = 7, Haplogroup B) occurred in northern Africa and Sardinia. These three mtDNA lineages were also evident when considered the 670 bp D-loop fragment (S5 Fig), although three additional haplotypes were found (Hap06’, Hap27’ and Hap37’).

In the Western European lineage (haplogroup A), three mtDNA haplotypes central to the phylogenetic network were identified (4, 10 and 20), from which various haplotypes and distinct evolutionary sub-lineages emerged. Apart from Iberia, these central haplotypes were also found in the British Isles (4, 10 and 20), Norway (10 and 20), Hungary and Italy (10) (**Fig 5**). The regions with the highest number of haplotypes were the Iberian Peninsula and the British Isles. Nevertheless, most haplotypes detected in these two regions were not shared, with the exception of the central haplotypes and the haplotype 11 (see S1 Table). The haplotype 49 identified in one specimen from the El Berguedà (BER) population corresponds to the same individual indicated as belonging to the European cluster in STRUCTURE and FCA analyses. Therefore, the specimens that carried haplotypes 11 and 49 were further excluded from phylogeographic analysis at the Iberian level.

**Fig 6:**
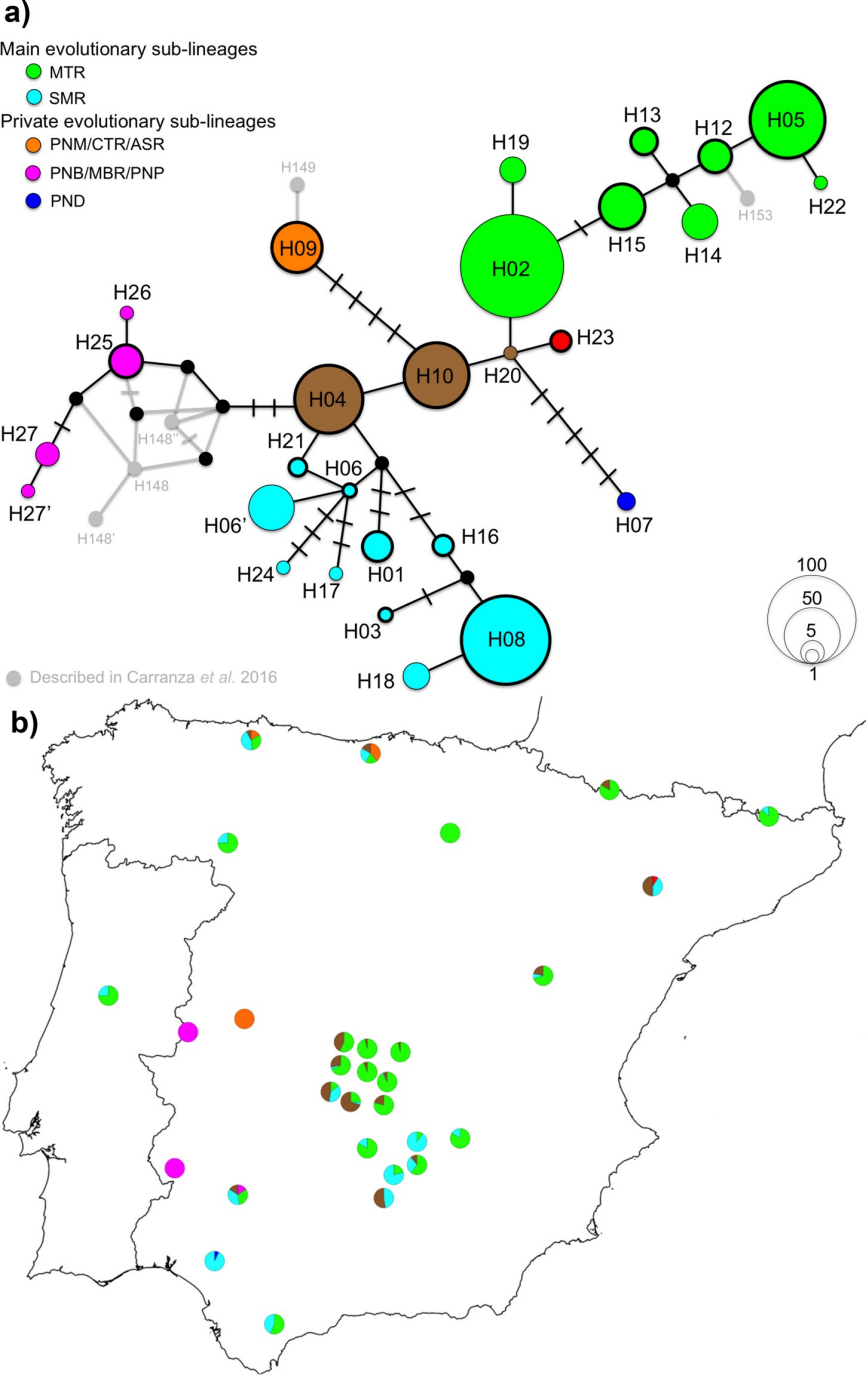
Gene genealogy and phylogenetic relationships of the red deer in the Iberian Peninsula using mtDNA D-loop fragment (650 bp). a) Median joining network showing the evolutionary relationship and the frequency of the Iberian haplotypes reported in this study. Haplotypes described distinctively in Carranza *et al*. [33] are depicted as small grey circles, while haplotypes reported in both studies are highlighted as circle thick line. Colour codes represent the various evolutionary sub-lineages reported in this study, including the central haplotypes, as represented in **Fig 5**. A bar in each solid line represents a mutational step; small black circles show undetected/extinct intermediate haplotype states. b) Distribution pattern of the evolutionary sub-lineages across Iberia. Color codes in the pie graphics represent the proportion of each sub-lineage by population.

The analysis of the 670 bp of the mtDNA D-loop identified 28 haplotypes among all the Iberian populations, which were maintained at 650 bp fragment size, but led to loss of two haplotypes (H06’ and H27’) when reduced to 329 bp (**Fig 6**, see details S1 Table). From the 20 haplotypes described across Iberia in the literature [33], we identified 15 and discovered 13 new haplotypes (650bp). The three mtDNA haplotypes central to the phylogenetic network previously described at the European level (4, 10 and 20) were also identified in the Iberian Peninsula. Several evolutionary sub-lineages emerged in Iberia from the central haplotypes. Whereas widespread across Iberia, the two main sub-lineages (green and blue in **Fig 6**) show some geographical structure: i) the green sub-lineage is present mainly in the central region (*Montes de Toledo*) and northern parts of Iberia; ii) the blue sub-lineage is observed in the southern region (*Sierra Morena*) of Iberia. The other sub-lineages were restricted to certain areas in Iberia (**Fig 6**). On the other hand, the central haplotypes (4, 10 and 20, in brown) are distributed mostly in the central and north-eastern populations.

### Population demography and divergence

Among the tested scenarios of population divergence using DIYABC (**Fig 2**), the one of simultaneous divergence of populations at t3 (Scenario 1) had the highest posterior probability at both European and Iberian levels (**Table 2**). Analyses to estimate the accuracy in scenario choice indicate that Type I and Type II errors for the best-supported scenario were moderately low for both analyses. Principal component analyses showed that summary statistics calculated for the posterior simulated datasets for the best-supported scenario explained the observed data well. Relative medians of absolute error values were moderately low for all parameters with the exception of t1 in Iberia analyses, indicating that estimates of posterior parameters are reliable (see S13 Table). Assuming an average generation time of 8.33 years for red deer [109], our results suggest that divergence among European populations (IB, FR, EN, NO, SE, CZ and HU) occurred between 5.1 and 21.1 ky BP (95% confidence interval, average 11.5 ky BP), and between 1.6 and 10.1 ky BP for Iberian populations (MTR, PNB, SMR and CFR; 95% confidence interval, average 5.0 ky BP).

**Table 2:** Posterior probability estimates and 95% confidence intervals (CI) for the five scenarios of divergence tested using the microsatellite data from European and Iberian populations of red deer, and using the logistic regression approach for approximate Bayesian computation (ABC) analyses. Type I and type II errors for the best-supported scenario are depicted.

	European					Iberian Peninsula			
Scenario	Posterior probability	95% CI	Type I error	Type II error	Scenario	Posterior probability	95% CI	Type I error	Type II error
**1**	0.651	0.584–0.718	0.097	0.109	1	0.860	0.843–0.876	0.083	0.259
**2**	0.206	0.154–0.257			2	0.075	0.064–0.086		
**3**	0.016	0.000–0.033			3	0.001	0.000–0.002		
**4**	0.105	0.069–0.140			4	0.061	0.051–0.069		
**5**	0.023	0.006–0.039			5	0.003	0.001–0.004		

**Fig 7:**
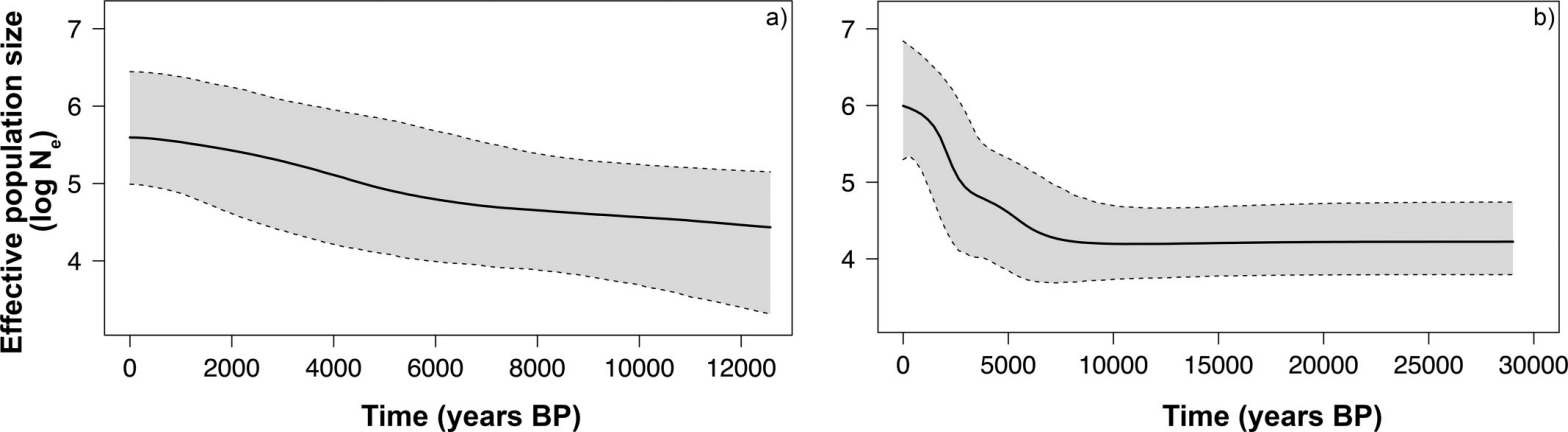
Bayesian Skyline Plots of the effective population size of red deer through time estimated from post-burn-in generations from two MCMC simulations, based on mtDNA data. The black line is the logarithm of median effective population size, log N_e_. The grey area bordered by dashed lines represent the 95% highest posterior density limits. a) Iberian haplotypes found in this study. b) British Isles haplotypes reported by Peréz-Espona *et al*. [71] and McDevitt *et al*. [68].

Using mitochondrial data and a within-species calibration model, the time to the most recent common ancestor (TMRCA) was inferred from BEAST for the three main European lineages (haplogroup A, B and C) and varies between 81.5 and 187.2 ky BP, with an average of 131.1 ky BP (**Table 3**). In addition, the TMRCA was estimated from all the Iberian haplotypes, varying between 12.1 and 38.4 ky BP, with an average of 24.2 ky BP (**Table 3**). Within the Iberian Peninsula, the putative historical demographic changes of red deer populations were also inferred using the Bayesian Skyline Plot approach. This analysis predicts an increasing population size over the last 12 ky for the Iberian populations (**Fig 7A**). When considering the populations from British Isles, one putative cryptic refugium for the species, the Bayesian Skyline Plot hindcasted a huge increase of population size approximately starting 7–8 ky BP (**Fig 7B)**.

**Table 3:** Bayesian coalescent estimates of divergence times (thousands of years before present—ky BP) among red deer mtDNA D-loop haplotypes (670 bp) at the Iberian Peninsula level and among the European lineages (haplogroups A, B and C; 329 bp) at the European level, inferred using an evolutionary rate estimated in this study*. Estimates from between-species fossil calibration method used in Skog *et al*. [19] are also shown.

Analysis	mtDNA D-loop	This study				Skog *et al*. [19]			
		Average	95% HPD lower	95% HPD upper	ESS	Average	95% HPD lower	95% HPD upper	ESS
**Iberian level**	All haplotypes	24.2	12.1	38.4	3.289				
	(H7)/(H04/10/20)	20.7	6.9	35.0	1.612				
	(H09)/(H04/10/20)	20.0	8.4	33.5	1.933				
	(H26/27/27’/28)/ (H04/10/20)	19.3	6.9	33.4	1.399				
**European level**	Haplogroup A/B/C	131.1	81.5	187.2	4.006	-	-	-	-
	Haplogroup A/B	128.3	78.6	185.7	4.310	303.7	209.1	411.0	7.123
	Haplogroup A/C	131.1	81.5	187.2	4.006	272.3	175.3	384.7	4.384
	Haplogroup B/C	131.0	81.4	187.2	3.995	295.2	207.1	398.6	6.613

* Calculations were done using a mutation rate of 3.849x10^-7^ nucleotide substitutions per site per year for the D-loop region (see details in Materials and Methods), while in Skog *et al*. [19] a mutation rate of 1.03x10^-7^ was used. At the Iberian level, the divergence time between the private evolutionary sub-lineages (H7—dark blue; H09 –orange; and H26/27/27’/28– pink; color codes as **Fig 6**) and mtDNA haplotypes central to the phylogenetic network (H4/10/20 –brown color **Fig 6**) were obtained. The average and 95% highest posterior density interval (HDP) together with effective samples sizes (ESS) are indicated.

### Species distribution models

**Fig 8:**
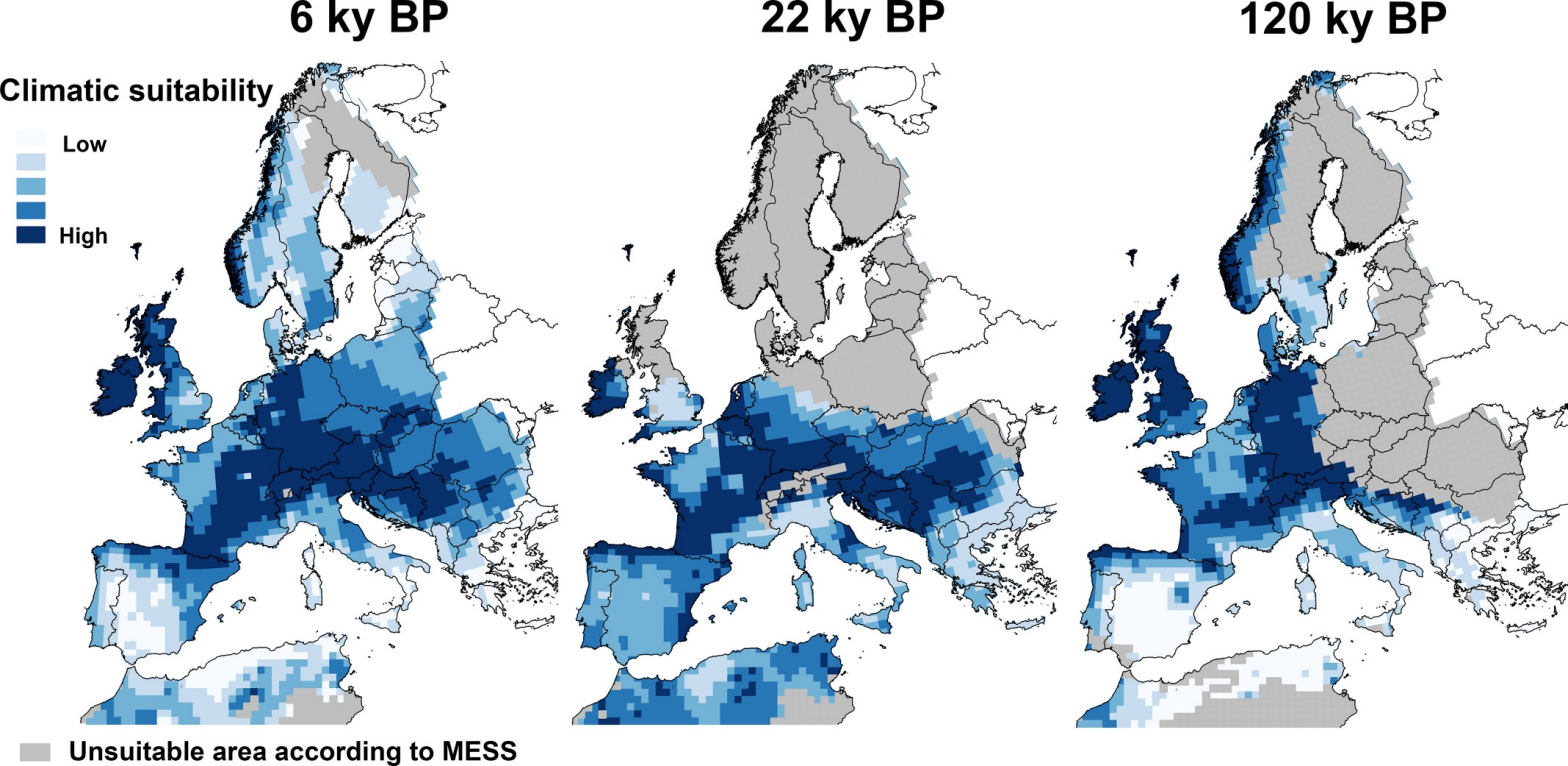
Climatic suitability for occurrence of red deer at 6 ky BP, 22 ky BP and 120 ky BP—according to the climatic niche for the species determined by GLM.

GLM achieved a reasonable predictive performance when it was evaluated on the validation dataset, both in terms of discrimination (ROC = 0.73) and reliability (H-L: χ^2^ = 6.58, df = 9, *P* = 0.582; see S6 Fig). The predicted red deer climatic suitability areas for the 6 ky BP, 22 ky BP and 120 ky BP periods are shown in **Fig 8**. At 120 ky BP, the predictions suggest that central Europe in general had a high climatic suitability for red deer, while unsuitable areas were hindcasted in north-eastern Europe by MESS. The predictions for 22 ky BP give highest climatic suitability values for some regions of the Iberian, Italian and Balkan Peninsulas, but also across central, eastern and western Europe. Although this pattern across central Europe was not so evident for the GBM, CTA and TSS model techniques compared with logistic regression (see S7 Fig), a clear high suitability area for red deer was suggested in southern France and Ireland in all models. Within Iberia, areas of high climatic suitability were predicted in north, east and west. In the case of the Mid-Holocene (6 ky BP), the predictions suggest that central and western Europe had high climatic suitability, while a fragmented pattern of climatic suitability was predicted for the Iberian Peninsula and North Africa. Areas of high climatic suitability were dispersed across Iberia, mainly in the north, east and west of the Peninsula, with a large area in central Iberia with low/absent climatic suitability (**Fig 8**).

#### Archaeological/paleontological data

**Fig 9:**
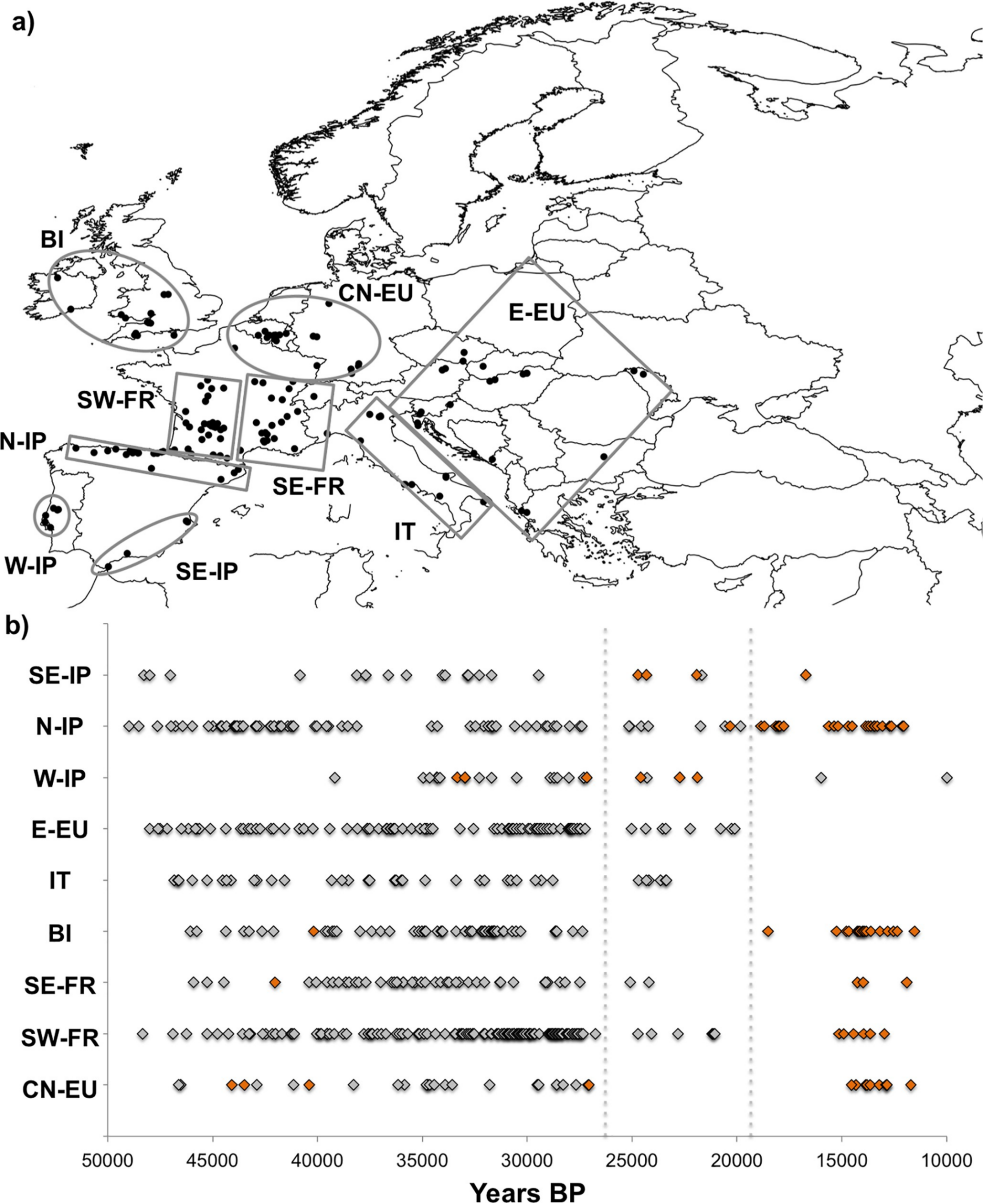
The red deer fossil records dated throughout the last 50 ky BP in Europe. a) the sampling locations were organized according to the favorable areas predicted by the 22 ky BP species distribution model: central-northern Europe, CN-EU; south-west France, SW-FR; south-east France, SE-FR; British Isles, BI; Italy, IT; East Europe, E-EU; west Iberian Peninsula, W-IP; north Iberian Peninsula, N-IP; south-east Iberian Peninsula, SE-IP; b) plot showing the presence of fossil red deer records directly (orange) or indirectly (grey) dated for each area identified above. Dotted lines represent the LGM interval (26.5 to 19 Ky BP) [110].

The geographic distribution range of the red deer fossil records dated throughout the last 50 ky BP in Europe is shown in **Fig 9A**. In general, the locations of dated fossil records within the LGM overlaps with climatic suitable areas predicted for red deer by the 22 ky BP (LGM) species distribution model, reinforcing the accuracy of spatial distribution modelling. From the archaeological data it is evident that red deer had a limited geographical distribution in central-northern Europe (CN-EU) and the British Isles (BI) during the LGM (**Fig 9A and 9B**). However, in addition to the Mediterranean peninsulas (south-east Iberian Peninsula, SE-IP; north Iberian Peninsula, N-IP; west Iberian Peninsula, W-IP; east Europe, E-EU; and Italy, IT), this species was also recorded in south-west France (SW-FR) and south-east France (SE-FR) during the LGM (see S8 Fig).

## Discussion

Clarifying in which regions species persisted during glaciations, as well as the timing and mode of postglacial expansions, is essential to understand evolutionary processes such as adaptation, speciation and extinction [14,111]. Species with a wide distribution range and a strong association with human activities, like the red deer, constitute an exciting and challenging case for understanding such processes, whether at a continental, regional or local scale. Here we used a multidisciplinary approach for inferring the evolutionary and demographic history of the red deer in Europe, particularly in the Iberian Peninsula, by integrating molecular and paleontological data with predictive species distribution modelling.

### Phylogeography of the red deer across Europe

**Fig 10:**
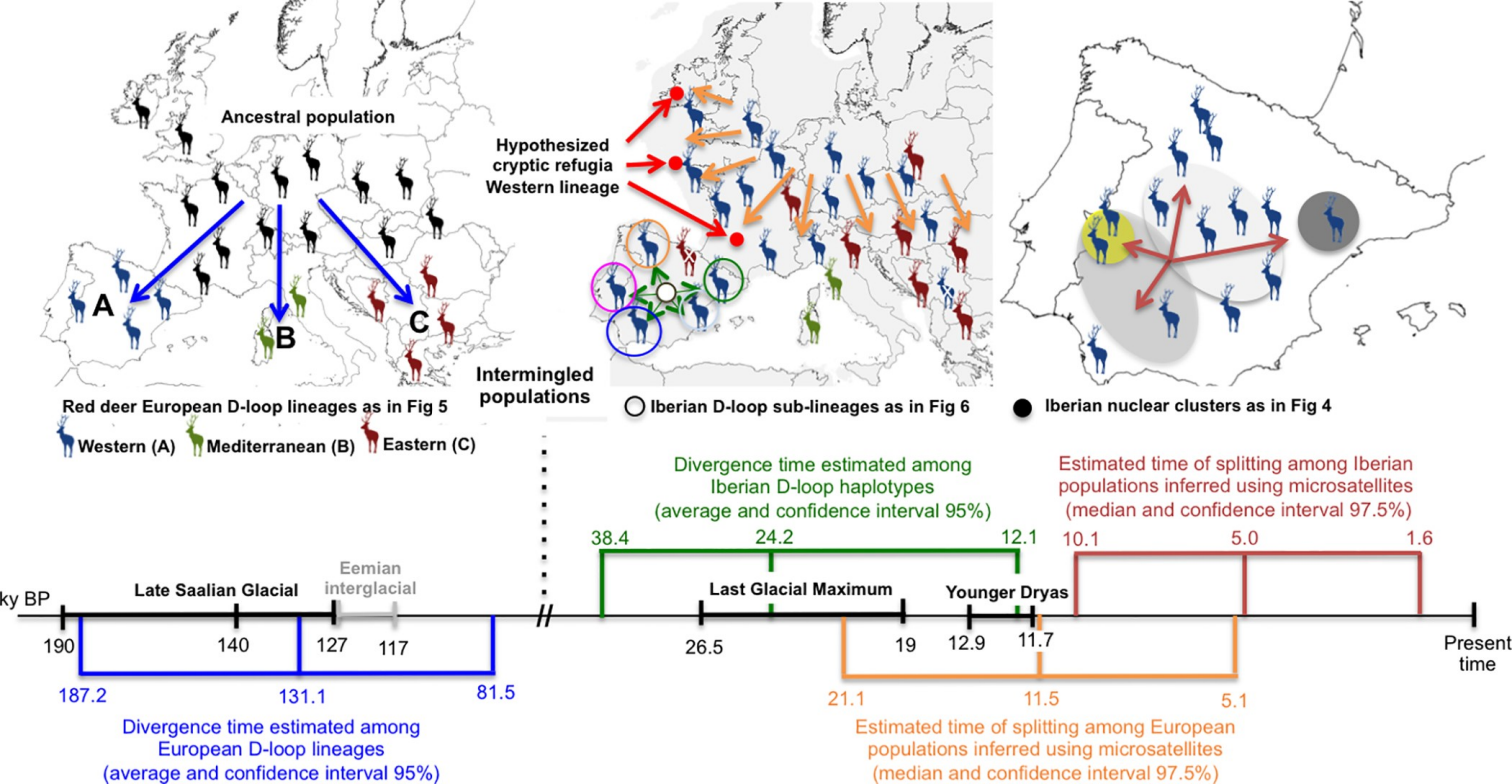
Timeframe of the evolutionary and demographic history of red deer in Europe proposed in this study, since 190 thousand years before present (ky BP) to the present time. The colored arrows in the graphics correspond to the biogeographical events summarized in the time-line. The divergence time inferred using the nuclear and mitochondrial data are also highlighted at the European and Iberian levels.

Mitochondrial D-loop sequences confirmed the three main red deer lineages previously reported by Ludt et al. [18] and Skog et al. [19] across Europe/Northern Africa: Western European lineage (haplogroup A); Mediterranean lineage (haplogroup B); and Eastern European lineage (haplogroup C). Isolation in multiple refugia in combination with several independent pre-glacial immigration routes from Asia was hypothesized by the previous authors [18,19] as the main reasons for the deep divergence among these three lineages. However, our results support a more recent divergence than suggested by Skog et al. [19] (**Table 3**). In our study the estimated divergence (average) among lineages was 131.1 ky BP (CI_95%_ 81.5–187.2), corresponding to a specific geological event–the Saalian Glacial Period (**Fig 10**). The divergence of the Western European lineage may be related to a partial discontinuity in red deer habitat suitability evident at 120 ky BP between western and eastern Europe (**Fig 8**), i.e. during the Eemian interglacial age (127–117 ky BP) [112] and presumably also present during the Saalian glacial period [113] that preceded it. During the Saalian glaciation, with its maximum at about 140 ky BP (Late Saalian, 190–127 ky BP), the Eurasian ice sheet reached further southward and eastward than during the LGM [114]. Therefore, most likely, isolation of red deer populations at the end of the Saalian glaciation promoted the differentiation of the mtDNA lineages. This division into Western and Eastern lineages is also evident from ancient fossil records throughout Europe, but with some exceptions to the general pattern [15,37]. Meiri et al. [15] suggest that the Eastern European haplotypes found in pre-LGM specimens from Spain, England and Belgium could be indicative of less prominent phylogeographical divisions (**Fig 10**). A similar panmictic distribution pattern was proposed by Rey-Iglesia et al. [37], in which authors have found evidence of Eastern European red deer haplotypes in pre-LGM times in Spain. The similarity found between some contemporary Iberian haplotypes and ancient pre-LGM specimens located in the Caucasus also concurs with this scenario of intermingled haplotypes subsequent to the initial divergence of the Eastern and Western lineages (S3 Table). Therefore, it appears that during the LGM, when there was a reduction in the areas of climatic suitability (**Fig 8**), the isolation and divergence between European lineages was reinforced, leading to the extinction of the Eastern European lineage from western Europe and the Western European lineage from eastern Europe (**Fig 10**). The best-supported scenario of divergence among the European populations is consistent with this isolation and further haplotype divergence since the LGM. The Mediterranean lineage (haplogroup B) appears to have diverged at the same time as the Western and Eastern European lineages. Although Skog et al. [19] found Mediterranean haplotypes in the Iberian Peninsula, our data suggest that the Mediterranean lineage is absent from the contemporary natural Iberian populations, which is also corroborated by its absence in ancient samples [15,37] and other contemporary Iberian populations [36]. A recent study using ancient DNA of radiocarbon-dated subfossils from Tyrrhenian island and mainland Italy identified the Italian Peninsula as the ultimate origin of the Mediterranean lineage and thus the Tyrrhenian and North African red deer [115].

#### The Western European red deer

Notwithstanding the intensive human-mediated movements of red deer across Europe [26,38], the distribution pattern of the Western European lineage (haplogroup A) is geographically coherent. Individuals from this lineage have mainly been found in western Europe, but they have also been described in the center and northeast of the continent. However, a previous wide-ranging study including populations from eastern Europe suggests that the Western European haplotypes found in Romania and Hungary have largely resulted from recent translocations, even though the Eastern European haplotypes present in these countries may reflect the natural northward expansion from southern refugia after the LGM [84]. Therefore, Western European haplotypes appear to have naturally dispersed from the Iberian Peninsula up to northern latitudes in Europe. Despite this widespread distribution and large haplotype variability, only the mtDNA haplotypes central to the phylogenetic network are shared between Iberia and central and northern European populations. Summarizing, we suggest a pre-LGM connection among red deer populations in Europe, as predicted for species distribution models at 120 ky BP in central Europe, followed by a separation between Iberian and central/northern European populations after the LGM. This hypothesis is also supported by the clear differentiation at nuclear markers observed between Iberian and the central/northern European populations, as well as by the timing of divergence estimated among them.

### The location of LGM refugia and their contribution to postglacial re-colonization of red deer in western Europe

The combination of mitochondrial and nuclear data, species distribution models, and fossil records, are consistent with cryptic refugia for the red deer in central and/or northern Europe, northwards of the traditional refugia in the southern Mediterranean peninsulas (**Fig 10**). Although these peninsulas were hindcasted with high climatic suitability, the same suitability was found for southern France and southern Ireland. Although the continental shelf between France and the British isles was not included in the species distribution models, owing to absence of bioclimatic variables for this region during the three temporal scales addressed in this study (6, 22 and 120 ky BP), recent predictions for this continental shelf during LGM showed highly suitable areas for both plant and animal species [116,117].

Therefore, the high levels of mitochondrial diversity observed in the British Isles together with the predictions obtained for species distribution models during LGM are consistent with a cryptic refugium in southern Ireland or in surrounding areas of continental shelf. In addition to southern Ireland, predicted as being suitable for many species during the LGM [118], recent analysis of ancient red deer specimens from Scotland, Ireland, and the Scottish isles Outer Hebrides and Inner Hebrides have found evidence that red deer from Outer Hebrides and Orkney Isles are unlikely to derive from mainland Scotland. One of the hypotheses raised by the authors is the possibility of red deer from those isles deriving from descendants of a deer population that inhabited a northern refugium during the LGM [118]. However, the absence of fossil records from the LGM in the British Isles (**Fig 9**; see also S8 Fig) makes this hypothesis unlikely, even though the surrounded areas of continental self remain unexplored for fossil remains. Furthermore, the high levels of mitochondrial diversity in red deer in the British Isles may also reflect the intensity of sampling for mitochondrial studies [67,68,71] and/or massive re-introductions from the rest of western Europe [80]. Moreover, a demographic expansion of red deer populations in the British Isles was only estimated around 7–4 ky BP, which is most simply explained by the impact on red deer populations of the transition from pre-Neolithic hunter-gatherer societies to Neolithic farming, known as the Neolithic revolution, which was one of the most pronounced cultural changes in European prehistory [119].

The alternative cryptic refugium for the red deer in western Europe is located in southern France due to the high habitat suitability and the presence of fossil records from the LGM (**Fig 8** and see S8 Fig). Data supporting cryptic refugia out of southern peninsulas have been proposed for various other plant and animal species in central and northern Europe [7,11,12,120–124] putting in question the traditional vision of the pre-eminence of southern Mediterranean refugia on European re-colonization history [6,7,9,14,122].

But, what is the role of central/northern LGM refugia in postglacial re-colonization of the red deer in western Europe? All present evidence suggests that the postglacial source of the current and widespread central and northern populations was located in the northern edge rather than the traditional southern refugia (Iberian Peninsula), as previously thought. This hypothesis is supported by the robust genetic differentiation observed between Iberian and European populations in both the nuclear and mitochondrial data (**Figs 3** and **5**). Moreover, the divergence timing (confidence interval) predicted among European populations is consistent with isolation after the LGM. This might be explained by the considerable barrier to gene flow between the southern and northern populations produced by the Pyrenees [29,31,125]. Thus, our hypothesis is that during the LGM red deer populations on both sides of Pyrenees suffered a population contraction, which led to a loss of Western European lineage diversity [15,37], leaving mostly the mtDNA haplotypes central to the phylogenetic network that are currently dispersed throughout Europe. At that time (the LGM), populations may have been connected by corridors on both sides (east and west) of the Pyrenees [28] due to lower sea-levels [126]. Nevertheless, when the ice sheets retreated, postglacial expansion apparently took place in opposite directions. Populations from south-western of France, southern Ireland or a region between them could rapidly expand to central and northern Europe because there was a connection between the British Isles and mainland Europe [126] (**Fig 10**). The small haplotype diversity observed in the central and northern European countries, excluding the British Isles, is consistent with this rapid expansion after the LGM [17,19], while the high mtDNA diversity currently found in the British Isles could be a result of the sampling bias/reintroductions mentioned above or a local refugium (in or surrounding) of this species during the LGM [26]. Sampling of ancient red deer specimens in the continental shelf and southern France would help to elucidate the precise location of cryptic refugia, since current populations in France mostly derive from restocking events after World War II, as a consequence of almost complete extirpation of populations over the middle of the nineteenth century [127].

Regarding the Iberian populations, the results of the spatial distribution model at 22 ky BP support a fragmented distribution of populations across Iberia during the LGM, with areas of high climate suitability in the north, east and southwest of the peninsula intermingled with areas of low suitability in the central region (**Fig 8**). This pattern is also consistent with the geographic distribution of fossil records during the LGM (see S8 Fig), and with the confidence intervals for the divergence times estimated among the Iberian sub-lineages (**Table 3**). After the LGM, populations might have evolved separately leading to the currently observed divergence of mitochondrial lineages. The fragmented pattern hindcasted at 6 ky BP for the red deer distribution and the time of divergence predicted among Iberian populations using microsatellite data (~5 ky BP) are also consistent with this past isolation of populations and the consequent divergent evolution (**Fig 10**).

### Mediterranean refugia: Understanding the evolutionary history of red deer

Strong genetic differentiation was observed between the Iberian and central/northern European populations, both at nuclear and mitochondrial levels. Some introgression from the central European populations was detected in the Iberian red deer populations analyzed (SLR, ASR, MT6, PNSAPA and PNM). This introgression might be explained by past human translocations from the central and northern European countries into Portugal and Spain [41,128].

The fact that the highest proportions of mtDNA haplotypes central to the phylogenetic network was present in central and north-eastern Iberia, including the unmanaged population in the Ebro Valley (CFR) suggests that the colonization process of the Iberian Peninsula might have occurred through the Pyrenees [18], rather than by the alternative hypothesis of red deer radiation via North Africa proposed by Geist [129] based on morphological characteristics. The hypothesis of a mainland central European source is also supported by the absence of Mediterranean lineage haplotypes [19] in the natural Iberian populations. Furthermore, according to our models, the central north-east of Iberia was the region with highest levels of habitat suitability at 120 ky BP (**Fig 8**).

Five mitochondrial (D-loop) sub-lineages can be identified in the Iberian Peninsula. The estimated TMRCA between these evolutionary sub-lineages and central haplotypes suggests a timing of divergence that overlaps with the LGM (**Table 3**). The isolation in multiple regions across Iberia during the LGM is also supported by the fragmented pattern predicted from spatial distribution models (**Fig 8**) and dated fossil records (**Fig 9**; see S8 Fig). As for many other species in Iberia, the red deer appears to have evolved divergently after the LGM, supporting the ‘refugia within refugia’ model [27]. The two sub-lineages, Montes de Toledo and Sierra Morena (green and blue in **Fig 6**, respectively) show high haplotype diversity and widespread distribution, while the other three sub-lineages (pink, dark blue and orange in **Fig 6**) display low haplotype diversity and have restricted distribution across Iberia, even when incorporating published haplotypes described recently in Iberia (**Fig 6**). These findings can be explained by historical demographic episodes described in Iberia over the last century [40,41,130]. The best-preserved areas of central Spain, in terms of habitat and the conservation of red deer (where large populations have persisted for decades—Montes de Toledo region (MTR) and Sierra Morena region (SMR) [39], have maintained high levels of haplotype diversity in each sub-lineage, while the reduced number of individuals after population contraction might explain the lack of/lower haplotype diversity observed in the other mitochondrial sub-lineages (**Fig 6**). The fact that the majority of the re-introduced animals during the seventies came from the Montes de Toledo and Sierra Morena mountain areas [40] may also explain the lack of a clear geographical pattern observed in these two sub-lineages. Comparing the geographical structure of mitochondrial with nuclear data, there is a clear correspondence between the Montes de Toledo and Sierra Morena sub-lineages and the two major clusters observed in STRUCTURE and FCA analyses (MTR and SMR) (**Fig 10**). Accordingly, the individuals from the PNB population, genetically differentiated in the FCA from the Sierra Morena cluster, represent most of specimens of the pink sub-lineage (**Fig 6**). Just one haplotype was found in the remnant two sub-lineages (dark blue and orange in **Fig 6**), which reinforced the lack of genetic signal in nuclear data.

Concerning the nuclear data, and despite the intensive restocking of animals since the 1950s [40], a clear pattern of genetic differentiation among populations is observed, with moderate evidence of isolation-by-distance (see S3 Fig). The three clusters (the Sierra Morena, Montes de Toledo and Caspe/Fraga) suggested by the STRUCTURE analysis, and the additional cluster identified in the FCA (the PNB cluster) make geographical sense and appear to have evolved divergently over the last 5 ky BP (CI_95%_1.6–10.1 ky BP). Areas of high habitat suitability during this period were dispersed across Iberia, with a large unsuitable area for red deer in central Iberia, consistent with isolation and divergent evolution (**Fig 8**). Montes de Toledo and Sierra Morena populations showed the highest effective population size estimated throughout Iberia, emphasizing the importance of these two areas as evolutionary units for the conservation of Iberian red deer genetic diversity. Furthermore, as a divergent evolutionary sub-lineage and distinct nuclear cluster, the PNB region is the other candidate evolutionary unit to preserve. Nonetheless, populations that harbor distinct haplotype sub-lineages, such as PNM and PND, should also be preserved in order to ensure haplotype diversity in the future.

### Implications for red deer conservation and management

Intensive management of red deer populations has occurred during the last century, both in the Iberian Peninsula and throughout Europe [17,38,40]. Nevertheless, we have evidence that the past demographic history of red deer populations, predominantly during the Late Pleistocene, may be the main factor responsible for the current phylogeographic pattern. The presence of a putative red deer refugium north of the traditional southern Mediterranean peninsulas during the LGM, and the clear genetic differentiation between Iberian and northern populations, indicates that, at least since the LGM, these populations have evolved and diverged in different ways. Both the divergence of thousands of years (promoted by isolation) and the probable local adaptation to environmental and climatic conditions has contributed to the evolution of several evolutionary units within this species in western Europe, which should be taken into consideration for red deer conservation [131]. This is important because translocations with poorly adapted individuals can disrupt the genetic integrity of natural populations and lead to a loss of local adaptations [132,133]. Moreover, the evolutionary units we describe in the Iberian Peninsula do not match the traditional taxonomic classification of red deer into a unique subspecies (see review in [17]), which may further hamper conservation strategies. The high range of genetic diversities observed among the studied populations (**Table 3**) may also reflect distinct management practices. High levels of inbreeding and low genetic diversity have been associated with different fitness-related traits in red deer such as trophies for hunting [134], reproductive success [135,136] and the capacity to overcome disease progression [42,137]. All these factors could compromise population viability in the near future [138]. Therefore, policymakers and landowners should make their management decisions based on the knowledge of genetic variation. This would favor the maintenance of local genetic diversity and avoid the effects of inbreeding, and thus ensure the future performance of populations under scenarios of climatic change or the emergence of new pathogens.

## Supporting information

S1 Table Mitochondrial D-loop phylogeography. The red deer mitochondrial D-loop haplotypes (329 bp) included in the phylogeographic analysis at a European level.
(XLSX)

S2 Table Mitochondrial D-loop phylogeography. Mitochondrial D-loop similarity between the red deer haplotypes found in the present study and those reported by Niedziałkowska *et al*. [84]. For this comparison a 248 bp fragment size was considered.
(DOCX)

S3 Table Mitochondrial D-loop phylogeography. Mitochondrial D-Loop similarity between the red deer haplotypes found in the present study and those reported by Meiri *et al*. [15]. For this comparison a 316 bp fragment size was considered, which after excluding nucleotide sites with gaps and missing data resulted in a total of 180 nucleotide sites analysed.
(DOCX)

S4 Table Mitochondrial D-loop phylogeography. Mitochondrial D-Loop similarity between the red deer haplotypes found in the present study and those reported by Rey-Iglesia *et al*. [37]. For this comparison a 670 bp fragment size was considered, which after excluding nucleotide sites with gaps and missing data resulted in a total of 449 nucleotide sites analysed.
(DOCX)

S5 Table Mitochondrial D-loop phylogeography. Mitochondrial D-Loop similarity between the red deer haplotypes found in the present study and those reported by Stanton *et al*. [26]. For this comparison a 328 bp fragment size was considered, which after excluding nucleotide sites with gaps and missing data resulted in a total of 264 nucleotide sites analysed.
(DOCX)

S6 Table Mitochondrial D-loop evolutionary rate. Ancient DNA mitochondrial D-loop sequences used for the inference of within-species evolutionary rate.
(DOCX)

S7 Table Mitochondrial D-loop evolutionary rate. Bayesian MCMC analysis performed to estimate the evolutionary rate of the D-loop mitochondrial fragment studied, using calibrated fossil ages of red deer from Europe (see S6 Table). Results of independent runs were combined using TRACER, version 1.5 [87], the final result was given based on average Log_10_ of the Bayes factor between models.
(DOCX)

S8 Table Species distribution modelling. The bioclimatic variables used to study the climatic requirements of *Cervus elaphus* distribution throughout western Europe and North Africa. A ‘quarter’ refers to a fraction of the year (i.e. three months). The same variables are available for present (1950–2000), Mid-Holocene (6 ky BP), Last Glacial Maximum (22 ky BP) and Last Interglacial period (120 ky BP).
(DOCX)

S9 Table Species distribution modelling. Results of the generalized linear model (GLM) model developed for the current distribution of *Cervus elaphus* in western Europe and North Africa. Predictors are listed following the order of inclusion in a stepwise procedure (the first one on top). The coefficient and its standard error (SE) and z-value test statistic values with significance levels are shown.
(DOCX)

S10 Table Species distribution modelling. Results of the generalized additive model (GAM), generalized boosting model (GBM), classification tree analysis (CTA), artificial neural network (ANN), flexible discriminant analysis (FDA) and, in addition, an ensemble of their forecasts, developed on the current distribution of *Cervus elaphus* in western Europe and North Africa.
(DOCX)

S11 Table Pairwise *F*_*ST*_ values for both microsatellite (above diagonal) and mitochondrial (below diagonal) datasets for the red deer populations studied. Population codes are described as in **Fig 1** of the main manuscript.
(DOCX)

S12 Table Population structure in the Iberian red deer. Number of individuals excluded from the Bayesian STRUCTURE analysis at the Iberian level, using microsatellite data. Exclusion was based on the individuals that had a membership proportion (qi) from the Iberian cluster lower than 95% in the main STRUCTURE analysis (all European populations) and in the European mtDNA phylogeographic analysis. Two individuals fulfilled both requisites.
(DOCX)

S13 Table Population demography and divergence inferred by the program DIYABC using microsatellite data. Posterior parameter estimates (median and 95% confidence intervals) for the best-supported scenario calculated using 1% of simulated datasets closest to the observed values. Simulations and approximate Bayesian computation analyses were performed including only nuclear makers and considering both nuclear and mitochondrial markers. Relative median absolute errors (RMAE) based on 500 pseudo-observed datasets are also given for each parameter. N1, N2, etc.—Effective population size of extant populations; t1—estimated date of lineage splitting in years (assuming a generation time of 8.33 years [104]); **μ**, mutation rate. Population codes are described as in **Fig 1** of the main manuscript.
(DOCX)

S1 Fig Analysis of population structure. Plots showing the results for both the Bayesian clustering analyses conducted in STRUCTURE software (see **Figs 3A and 4A** and S4 Fig) and the highest Δ*K* value obtained following Evanno *et al*. [59] procedures.
(DOCX)

S2 Fig European red deer differentiation inferred using nuclear and mitochondrial markers. Neighbor-joining trees representing the genetic differentiation among populations measured as pairwise *F*_*ST*_ for both microsatellite (left) and mtDNA (right) datasets. Population codes are described in **Fig 1** of the main manuscript.
(DOCX)

S3 Fig Isolation-by-distance in the Iberian red deer populations. Plots showing the relationship between genetic distance [pairwise *F*_*ST*_/(1-*F*_*ST*_)] and geographic distance (log km) between the Iberian red deer populations quantified for both microsatellites and mitochondrial datasets (isolation-by-distance).
(DOCX)

S4 Fig Population structure of the European red deer populations. Bayesian clustering analyses performed in STRUCTURE [56] on microsatellite data from the central and north European red deer populations, considering the best Δ*K* values obtained following Evanno *et al*. [59] procedures (see also S1 Fig). Proportional membership to each cluster is indicated for *K* = 3 and *K* = 8. Population codes are described as in **Fig 1** of the main manuscript.
(DOCX)

S5 Fig Mitochondrial D-loop phylogeography of red deer. Median joining network showing the evolutionary relationship of D-loop haplotypes found in this study (670 bp fragment size) for the red deer in Europe. Numbers within circles correspond to haplotype name described in the main manuscript for the 329 bp haplotypes (see details in S1 Table). A bar on each solid line represents a mutational step; small black circles show undetected/extinct intermediate haplotype states; color codes within the circles are depicted for Western European lineage (haplogroup A) and represent the country where the haplotypes were found. The Iberian sub-lineages are grouped by colored shading following **Fig 6**.
(DOCX)

S6 Fig Species distribution modelling. Calibration plot showing the relationship between the predicted probability for red deer occurrence according to its climatic niche and the observed frequency of the species in the validation dataset. Open symbols indicate bins with < 15 localities, in which the frequency observed should be considered with caution [139].
(DOCX)

S7 Fig Species distribution modelling. Climatic suitability for the occurrence of *Cervus elaphus* in western Europe and North Africa during the Mid-Holocene (6 ky BP), Last Glacial Maximum (LGM, 22 ky BP) and interglacial period (120 ky BP) represented for the generalized boosting model (GBM), classification tree analysis (CTA) and the ensemble of their forecasts, according to the statistical model shown in S10 Table.
(DOCX)

S8 Fig Archaeological data and species distribution model for red deer during the LGM. Map showing the geographic distribution of the red deer fossil records dated within the period of Last Glacial Maximum (**Fig 9**) and the climatic suitability for occurrence of red deer at 22 kyBP (**Fig 8**), predicted according to the climatic niche for the species determined by a generalized linear model (GLM) model.
(DOCX)
