# Supplementary material for: Red deer in Iberia: Molecular ecological studies in a southern refugium and inferences on European postglacial colonization history
Source: PLoS One. 2019 Jan 8;14(1):e0210282. doi: 10.1371/journal.pone.0210282 (PMC6324796; doi:10.1371/journal.pone.0210282)
Supplement: S2 Table — Mitochondrial D-loop similarity between the red deer haplotypes found in the present study and those reported by Niedziałkowska et al. [84]. For this comparison a 248 bp fragment size was considered. (DOCX) [file pone.0210282.s002.docx]

**S2 Table:** Mitochondrial D-loop similarity between the red deer haplotypes found in the present study and those reported by Niedziałkowska *et al.* [84]. For this comparison a 248 bp fragment size was considered.

| **Haplotype** | **Reference** | **Name** | **GENBANK accession number** | **Location** |
| --- | --- | --- | --- | --- |
| H01 | In this study | Hap01 |  | Iberian Peninsula |
| H02 | In this study | Hap02 |  | Iberian Peninsula |
| H03 | In this study | Hap03 |  | Iberian Peninsula |
| H04 | In this study | Hap04 |  | Iberian Peninsula |
| H04 | In this study | Hap10 |  | Iberian Peninsula, Italy |
| H04 | In this study | Hap25 |  | Iberian Peninsula |
| H04 | In this study | Hap43 |  | Czech Republic |
| H05 | In this study | Hap05 |  | Iberian Peninsula |
| H05 | In this study | Hap22 |  | Iberian Peninsula |
| H06 | In this study | Hap06 |  | Iberian Peninsula |
| H06 | In this study | Hap06´ |  | Iberian Peninsula |
| H07 | In this study | Hap07 |  | Iberian Peninsula |
| H08 | In this study | Hap08 |  | Iberian Peninsula |
| H09 | In this study | Hap09 |  | Iberian Peninsula |
| H10 | In this study | Hap11 |  | Iberian Peninsula |
| H11 | In this study | Hap12 |  | Iberian Peninsula |
| H12 | In this study | Hap13 |  | Iberian Peninsula |
| H13 | In this study | Hap14 |  | Iberian Peninsula |
| H14 | In this study | Hap15 |  | Iberian Peninsula |
| H15 | In this study | Hap16 |  | Iberian Peninsula |
| H16 | In this study | Hap17 |  | Iberian Peninsula |
| H17 | In this study | Hap18 |  | Iberian Peninsula |
| H18 | In this study | Hap19 |  | Iberian Peninsula |
| H19 | Niedziałkowska et al. 2011 | 342GolMNBialow | HQ290007 | Poland |
| H19 | In this study | Hap20 |  | Iberian Peninsula |
| H19 | In this study | Hap26 |  | Iberian Peninsula |
| H20 | In this study | Hap21 |  | Iberian Peninsula |
| H21 | Niedziałkowska et al. 2011 | 618SokMNBialow | HQ290014 | Poland, Germany |
| H21 | In this study | Hap23 |  | Iberian Peninsula |
| H22 | In this study | Hap24 |  | Iberian Peninsula |
| H23 | In this study | Hap27 |  | Iberian Peninsula |
| H23 | In this study | Hap27´ |  | Iberian Peninsula |
| H24 | In this study | Hap28 |  | Iberian Peninsula |
| H25 | In this study | Hap29 |  | England |
| H26 | In this study | Hap30 |  | England |
| H27 | In this study | Hap31 |  | England |
| H27 | In this study | Hap47 |  | Norway |
| H28 | In this study | Hap32 |  | England, Sweden, Italy |
| H29 | In this study | Hap33 |  | England |
| H30 | In this study | Hap34 |  | Switzerland, Italy |
| H31 | Niedziałkowska et al. 2011 | 545LitMNBialow | HQ290022 | Polish Carpathians |
| H31 | In this study | Hap35 |  | France |
| H32 | In this study | Hap36 |  | France |
| H33 | In this study | Hap37 |  | Switzerland, Czech Republic |
| H33 | In this study | Hap39 |  | Switzerland |
| H33 | Niedziałkowska et al. 2011 | Hp5LauFZKiel | HQ290070 | Germany |
| H34 | Niedziałkowska et al. 2011 | 724OlesMNBialow | HQ290015 | Poland |
| H34 | In this study | Hap38 |  | Switzerland, Hungary |
| H35 | Niedziałkowska et al. 2011 | 732GermMNBialow | HQ290021 | Germany |
| H35 | In this study | Hap40 |  | Switzerland |
| H36 | Niedziałkowska et al. 2011 | 639GomMNBialow | HQ290016 | Belarus |
| H36 | In this study | Hap41 |  | Switzerland |
| H37 | Niedziałkowska et al. 2011 | 620PoddMNBialow | HQ290017 | Poland, Polish Carpathians |
| H37 | In this study | Hap42 |  | Hungary |
| H37 | In this study | Hap45 |  | Italy |
| H38 | In this study | Hap44 |  | Czech Republic |
| H39 | In this study | Hap46 |  | Italy |
| H40 | In this study | Hap48 |  | Norway |
| H41 | Niedziałkowska et al. 2011 | 47GolMNBialow | HQ290005 | Poland, Germany |
| H42 | Niedziałkowska et al. 2011 | 344GolMNBialow | HQ290006 | Poland, Germany |
| H43 | Niedziałkowska et al. 2011 | 326GolMNBialow | HQ290008 | Poland, Belarus, Lithuania, Germany |
| H44 | Niedziałkowska et al. 2011 | 230BialMNBialow | HQ290009 | Poland, Belarus |
| H45 | Niedziałkowska et al. 2011 | 95BorkiMNBialow | HQ290010 | Poland |
| H46 | Niedziałkowska et al. 2011 | 655UkrMNBialow | HQ290011 | Ukraine, Polish Carpathians |
| H47 | Niedziałkowska et al. 2011 | 623BrynMNBialow | HQ290012 | Poland, Germany |
| H48 | Niedziałkowska et al. 2011 | 646RusMNBialow | HQ290013 | Belarus, Lithuania, Russia |
| H49 | Niedziałkowska et al. 2011 | 748GermMNBialow | HQ290018 | Germany |
| H50 | Niedziałkowska et al. 2011 | 636VitMNBialow | HQ290019 | Belarus |
| H51 | Niedziałkowska et al. 2011 | 736GermMNBialow | HQ290020 | Germany |
| H52 | Niedziałkowska et al. 2011 | 562BiesMNBialow | HQ290023 | Polish Carpathians |
| H53 | Niedziałkowska et al. 2011 | 563BiesMNBialow | HQ290024 | Germany, Lithuania |
| H53 | Niedziałkowska et al. 2011 | Lu9FZKiel | HQ290072 | Polish Carpathians |
| H54 | Niedziałkowska et al. 2011 | MV51FZKiel | HQ290073 | Germany |
| H55 | Niedziałkowska et al. 2011 | 2DenmFZKiel | HQ290066 | Denmark |
| H56 | Niedziałkowska et al. 2011 | Hp2LauFZKiel | HQ290067 | Germany |
| H57 | Niedziałkowska et al. 2011 | Hp3LauFZKiel | HQ290068 | Germany |
| H58 | Niedziałkowska et al. 2011 | Hp4LauFZKiel | HQ290069 | Germany |
| H59 | Niedziałkowska et al. 2011 | Ba4FZKiel | HQ290071 | Polish Carpathians |
