# Supplementary material for: Red deer in Iberia: Molecular ecological studies in a southern refugium and inferences on European postglacial colonization history
Source: PLoS One. 2019 Jan 8;14(1):e0210282. doi: 10.1371/journal.pone.0210282 (PMC6324796; doi:10.1371/journal.pone.0210282)
Supplement: S3 Table — Mitochondrial D-Loop similarity between the red deer haplotypes found in the present study and those reported by Meiri et al. [15]. For this comparison a 316 bp fragment size was considered, which after excluding nucleotide sites with gaps and missing data resulted in a total of 180 nucleotide sites analysed. (DOCX) [file pone.0210282.s003.docx]

**S3 Table:** Mitochondrial D-Loop similarity between the red deer haplotypes found in the present study and those reported by Meiri *et al.* [15]. For this comparison a 316 bp fragment size was considered, which after excluding nucleotide sites with gaps and missing data resulted in a total of 180 nucleotide sites analysed.

| **Haplotype** | **Reference** | **Name** | **GENBANK accession number** | **Location** | **Age (uncal. BP)/ Stratigraphy** |
| --- | --- | --- | --- | --- | --- |
| H01 | In this study | Hap01 |  | Iberian Peninsula |  |
| H01 | In this study | Hap27 |  | Iberian Peninsula |  |
| H01 | In this study | Hap27´ |  | Iberian Peninsula |  |
| H02 | Meiri et al. 2013 | D52 | KF133970 | New Zealand, Otago | |
| H02 | Meiri et al. 2013 | D53 | KF133971 | New Zealand, Otago | |
| H02 | In this study | Hap02 |  | Iberian Peninsula |  |
| H02 | In this study | Hap11 |  | Iberian Peninsula |  |
| H02 | In this study | Hap17 |  | Iberian Peninsula |  |
| H02 | In this study | Hap20 |  | Iberian Peninsula |  |
| H02 | In this study | Hap23 |  | Iberian Peninsula |  |
| H02 | In this study | Hap26 |  | Iberian Peninsula |  |
| H02 | In this study | Hap28 |  | Iberian Peninsula |  |
| H02 | In this study | Hap29 |  | England |  |
| H02 | In this study | Hap35 |  | France |  |
| H02 | In this study | Hap36 |  | France |  |
| H02 | In this study | Hap48 |  | Norway |  |
| H02 | Meiri et al. 2013 | MM079 | KF133905 | Spain, El Mirón Cave | 11,785±55 |
| H02 | Meiri et al. 2013 | MM150 | KF133914 | England, Longstones Field | 4193±35 |
| H02 | Meiri et al. 2013 | MM165 | KF133917 | Serbia, Lepenski Vir S47 | 10,000±45 |
| H02 | Meiri et al. 2013 | MM183 | KF133919 | Scotland, Isle of Risga | 5875±65 |
| H02 | Meiri et al. 2013 | MM187 | KF133921 | Spain, El Mirón Cave | 15,610±90 |
| H02 | Meiri et al. 2013 | MM190 | KF133922 | Serbia, Lepenski Vir S46 | 7912±39 |
| H02 | Meiri et al. 2013 | MM192 | KF133923 | Caucasus, Mezmaiska Cave | >44,700 |
| H02 | Meiri et al. 2013 | MM198 | KF133924 | England, Ossoms Cave | 12,310±50 |
| H02 | Meiri et al. 2013 | MM214 | KF133926 | Caucasus, Mezmaiska Cave | 50,100±4000 |
| H02 | Meiri et al. 2013 | MM224 | KF133927 | Caucasus, Mezmaiska Cave | >50,200 |
| H02 | Meiri et al. 2013 | MM335 | KF133949 | Belarus, Belovezhskaya Pushcha Reserve | 1960 |
| H02 | Meiri et al. 2013 | MM337 | KF133950 | Russia, Karelia | 1991 |
| H02 | Meiri et al. 2013 | MM352 | KF133933 | Caucasus, Mezmaiska Cave | |
| H02 | Meiri et al. 2013 | MM455 | KF133937 | England, Kent's Cavern | pre-LGM: between ca. 60,000 and 25,000 cal. yr BP |
| H02 | Meiri et al. 2013 | MM702 | KF133947 | Czech Republic, Homolka Pit 31a | |
| H02 | Meiri et al. 2013 | MM718 | KF133960 | Spain, Castilla-La Mancha | |
| H02 | Meiri et al. 2013 | MM719 | KF133951 | Germany, Hamburg | 1949 |
| H02 | Meiri et al. 2013 | MM732 | KF133968 | Scotland, Inverness- Shire Glenquoich | 1971 |
| H02 | Meiri et al. 2013 | MM733 | KF133969 | Scotland, Balmoral | 1962 |
| H02 | Meiri et al. 2013 | MM762 | KF133946 | Norway, Etne | 1996 |
| H02 | Meiri et al. 2013 | MM763 | KF133955 | Norway, Etne | 1996 |
| H02 | Meiri et al. 2013 | MM764 | KF133956 | Norway, Etne | 1996 |
| H02 | Meiri et al. 2013 | MM765 | KF133957 | Norway, Sveio | 1996 |
| H02 | Meiri et al. 2013 | MM770 | KF133954 | Sweden, Skane | 1882/8/09 |
| H02 | Meiri et al. 2013 | MM781 | KF133961 | Italy, Mesola Wood (Gran Bosco della Mesola) | |
| H02 | Meiri et al. 2013 | MM782 | KF133962 | Italy, Mesola Wood (Gran Bosco della Mesola) | |
| H03 | In this study | Hap03 |  | Iberian Peninsula |  |
| H03 | In this study | Hap08 |  | Iberian Peninsula |  |
| H03 | In this study | Hap18 |  | Iberian Peninsula |  |
| H04 | In this study | Hap04 |  | Iberian Peninsula |  |
| H04 | In this study | Hap06 |  | Iberian Peninsula |  |
| H04 | In this study | Hap06´ |  | Iberian Peninsula |  |
| H04 | In this study | Hap10 |  | Iberian Peninsula, Italy | |
| H04 | In this study | Hap21 |  | Iberian Peninsula |  |
| H04 | In this study | Hap24 |  | Iberian Peninsula |  |
| H04 | In this study | Hap25 |  | Iberian Peninsula |  |
| H04 | In this study | Hap32 |  | England, Sweden, Italy | |
| H04 | In this study | Hap33 |  | England |  |
| H04 | In this study | Hap40 |  | Switzerland |  |
| H04 | In this study | Hap43 |  | Czech Republic |  |
| H04 | In this study | Hap47 |  | Norway |  |
| H04 | Meiri et al. 2013 | MM080 | KF133906 | Spain, El Mirón Cave | 11,205±55 |
| H04 | Meiri et al. 2013 | MM131 | KF133911 | England, Elder Bush Cave | 10,600±110 |
| H04 | Meiri et al. 2013 | MM139 | KF133913 | France, Prissé-la-Charrière | 5460±45 |
| H04 | Meiri et al. 2013 | MM186 | KF133920 | Scotland, Shewalton | 5840±80 |
| H04 | Meiri et al. 2013 | MM235 | KF133928 | Belgium, Trou Al'Wesse Couche 4a | Neolithic |
| H04 | Meiri et al. 2013 | MM239 | KF133929 | Belgium, Trou Al'Wesse Couche 4b | Mesolithic |
| H04 | Meiri et al. 2013 | MM251 | KF133931 | England, Chelm's Combe, Cheddar | 10,910±110 |
| H04 | Meiri et al. 2013 | MM464 | KF133939 | Spain, Mazaculos Shelter | Neolithic |
| H04 | Meiri et al. 2013 | MM468 | KF133943 | Spain, Mazaculos Shelter | Neolithic |
| H04 | Meiri et al. 2013 | MM475 | KF133942 | Spain, Mazaculos Shelter | Austurian |
| H04 | Meiri et al. 2013 | MM477 | KF133944 | Spain, Mazaculos Shelter | Austurian |
| H04 | Meiri et al. 2013 | MM493 | KF133945 | Spain, Mazaculos Shelter | Austurian |
| H04 | Meiri et al. 2013 | MM721 | KF133958 | Spain, Coto Doñana Huelua | 1895, 1908 |
| H04 | Meiri et al. 2013 | MM731 | KF133959 | Spain, Coto Doñana Huelua | 1895, 1908 |
| H05 | In this study | Hap05 |  | Iberian Peninsula |  |
| H05 | In this study | Hap12 |  | Iberian Peninsula |  |
| H05 | In this study | Hap22 |  | Iberian Peninsula |  |
| H06 | In this study | Hap07 |  | Iberian Peninsula |  |
| H06 | In this study | Hap37 |  | Switzerland, Czech Republic | |
| H06 | Meiri et al. 2013 | MM461 | KF133938 | Spain, Mazaculos Shelter | Neolithic |
| H06 | Meiri et al. 2013 | MM472 | KF133940 | Spain, Mazaculos Shelter | Neolithic |
| H07 | In this study | Hap09 |  | Iberian Peninsula |  |
| H08 | In this study | Hap13 |  | Iberian Peninsula |  |
| H09 | In this study | Hap14 |  | Iberian Peninsula |  |
| H09 | In this study | Hap15 |  | Iberian Peninsula |  |
| H10 | In this study | Hap16 |  | Iberian Peninsula |  |
| H11 | In this study | Hap19 |  | Iberian Peninsula |  |
| H12 | In this study | Hap30 |  | England |  |
| H13 | In this study | Hap31 |  | England |  |
| H14 | In this study | Hap34 |  | Switzerland, Italy |  |
| H14 | In this study | Hap46 |  | Italy |  |
| H15 | In this study | Hap38 |  | Switzerland, Hungary | |
| H15 | In this study | Hap42 |  | Hungary |  |
| H15 | In this study | Hap45 |  | Italy |  |
| H15 | Meiri et al. 2013 | MM153 | KF133915 | Serbia, Rudna Glava S7 | 7198±36 |
| H15 | Meiri et al. 2013 | MM331 | KF133967 | Georgia, Borzon | 1867 |
| H15 | Meiri et al. 2013 | MM379 | KF133934 | Urals | >45,500 |
| H15 | Meiri et al. 2013 | MM754 | KF133952 | Italy, Southern Apennine Mountains, Molise | |
| H15 | Meiri et al. 2013 | MM755 | KF133953 | Italy, Southern Apennine Mountains, Molise | |
| H16 | In this study | Hap39 |  | Switzerland |  |
| H17 | In this study | Hap41 |  | Switzerland |  |
| H18 | In this study | Hap44 |  | Czech Republic |  |
| H19 | Meiri et al. 2013 | MM007 | KF133903 | Spain, El Mirón Cave | Lower Magdalenian |
| H20 | Meiri et al. 2013 | MM020 | KF133904 | Caucasus, North Ossetia, Cave Lasok | 32,040±180 |
| H21 | Meiri et al. 2013 | MM082 | KF133907 | Spain, El Mirón Cave | 14,930±70 |
| H21 | Meiri et al. 2013 | MM093 | KF133908 | Spain, El Mirón Cave | 14,760±70, 14,795±75 |
| H22 | Meiri et al. 2013 | MM095 | KF133909 | Spain, El Mirón Cave | Magdalenian |
| H23 | Meiri et al. 2013 | MM098 | KF133910 | Spain, El Mirón Cave | 15,430±75 |
| H24 | Meiri et al. 2013 | MM135 | KF133912 | France, Le Bois Ragot | 12,585±75 |
| H25 | Meiri et al. 2013 | MM154 | KF133916 | England, Longstones Field | 4216±36 |
| H26 | Meiri et al. 2013 | MM175 | KF133918 | England, Hyena Den, Wookey Hole | 11,320±120 |
| H27 | Meiri et al. 2013 | MM210 | KF133925 | North Sea | 8870±50 |
| H28 | Meiri et al. 2013 | MM245 | KF133930 | Belgium, Trou Al'Wesse Couche 15 | 40,200±1300 |
| H29 | Meiri et al. 2013 | MM272 | KF133932 | Urals | 44,650±650 |
| H29 | Meiri et al. 2013 | MM473 | KF133941 | Germany, Rosenbeck | |
| H30 | Meiri et al. 2013 | MM408 | KF133935 | Jordan, Tell Hesban | 864±28 |
| H31 | Meiri et al. 2013 | MM415 | KF133936 | Jordan, Tell Hesban | |
| H32 | Meiri et al. 2013 | MM313 | KF133963 | Russia, Voronezh | 1958 |
| H32 | Meiri et al. 2013 | MM334 | KF133948 | Russia, Voronezh | 1958 |
| H33 | Meiri et al. 2013 | MM315 | KF133964 | Russia, Krasnodarsky Krai, Caucasus | 1909, 1972 |
| H33 | Meiri et al. 2013 | MM317 | KF133965 | Russia, Krasnodarsky Krai, Caucasus | 1909, 1972 |
| H34 | Meiri et al. 2013 | MM766 | KF133966 | Turkey, Central Anatolia | 1994 |
