# Supplementary material for: Red deer in Iberia: Molecular ecological studies in a southern refugium and inferences on European postglacial colonization history
Source: PLoS One. 2019 Jan 8;14(1):e0210282. doi: 10.1371/journal.pone.0210282 (PMC6324796; doi:10.1371/journal.pone.0210282)
Supplement: S4 Table — Mitochondrial D-Loop similarity between the red deer haplotypes found in the present study and those reported by Rey-Iglesia et al. [37]. For this comparison a 670 bp fragment size was considered, which after excluding nucleotide sites with gaps and missing data resulted in a total of 449 nucleotide sites analysed. (DOCX) [file pone.0210282.s004.docx]

**S4 Table:** Mitochondrial D-Loop similarity between the red deer haplotypes found in the present study and those reported by Rey-Iglesia *et al.* [37]. For this comparison a 670 bp fragment size was considered, which after excluding nucleotide sites with gaps and missing data resulted in a total of 449 nucleotide sites analysed.

| **Haplotype** | **Reference** | **Name** | **GENBANK accession number** | **Location** | **Age**  **(^14^C years BP) Stratigraphy** |
| --- | --- | --- | --- | --- | --- |
| H01 | In this study | Hap01 |  | Iberian Peninsula |  |
| H02 | In this study | Hap02 |  | Iberian Peninsula |  |
| H02 | In this study | Hap19 |  | Iberian Peninsula |  |
| H03 | In this study | Hap03 |  | Iberian Peninsula |  |
| H04 | In this study | Hap04 |  | Iberian Peninsula |  |
| H04 | In this study | Hap21 |  | Iberian Peninsula |  |
| H04 | In this study | Hap31 |  | England |  |
| H05 | In this study | Hap05 |  | Iberian Peninsula |  |
| H06 | In this study | Hap06 |  | Iberian Peninsula |  |
| H07 | In this study | Hap07 |  | Iberian Peninsula |  |
| H08 | In this study | Hap06´ |  | Iberian Peninsula |  |
| H09 | In this study | Hap08 |  | Iberian Peninsula |  |
| H09 | In this study | Hap18 |  | Iberian Peninsula |  |
| H10 | In this study | Hap09 |  | Iberian Peninsula |  |
| H10 | In this study | Hap47 |  | Norway |  |
| H10 | Rey-Iglesia et al. 2017 | ZMUC2 | MF872247 | Denmark | 4031±30 |
| H11 | In this study | Hap10 |  | Iberian Peninsula, Italy |  |
| H12 | In this study | Hap11 |  | Iberian Peninsula |  |
| H13 | In this study | Hap12 |  | Iberian Peninsula |  |
| H14 | In this study | Hap13 |  | Iberian Peninsula |  |
| H15 | In this study | Hap14 |  | Iberian Peninsula |  |
| H16 | In this study | Hap15 |  | Iberian Peninsula |  |
| H17 | In this study | Hap16 |  | Iberian Peninsula |  |
| H18 | In this study | Hap17 |  | Iberian Peninsula |  |
| H19 | Rey-Iglesia et al. 2017 | CGG-1-014533 | MF872246 | Spain, Liñares Cave | >38.000 |
| H19 | In this study | Hap20 |  | Iberian Peninsula |  |
| H20 | In this study | Hap22 |  | Iberian Peninsula |  |
| H21 | Rey-Iglesia et al. 2017 | CGG-1-016150 | MF872240 | Spain, Liñares Cave | >38.000 |
| H21 | Rey-Iglesia et al. 2017 | CGG-1-016151 | MF872245 | Spain, Liñares Cave | >38.000 |
| H21 | In this study | Hap23 |  | Iberian Peninsula |  |
| H22 | In this study | Hap24 |  | Iberian Peninsula |  |
| H23 | In this study | Hap25 |  | Iberian Peninsula |  |
| H24 | In this study | Hap26 |  | Iberian Peninsula |  |
| H25 | In this study | Hap27 |  | Iberian Peninsula |  |
| H26 | In this study | Hap28 |  | Iberian Peninsula |  |
| H26 | Rey-Iglesia et al. 2017 | ZMUC5 | MF872248 | Denmark | 9557±50 |
| H27 | In this study | Hap27´ |  | Iberian Peninsula |  |
| H28 | In this study | Hap29 |  | England |  |
| H29 | In this study | Hap30 |  | England |  |
| H29 | In this study | Hap35 |  | France |  |
| H30 | In this study | Hap32 |  | England, Sweden, Italy |  |
| H31 | In this study | Hap33 |  | England |  |
| H32 | In this study | Hap34 |  | Switzerland, Italy |  |
| H33 | In this study | Hap36 |  | France |  |
| H34 | In this study | Hap37 |  | Switzerland, Czech Republic |  |
| H34 | In this study | Hap39 |  | Switzerland |  |
| H35 | In this study | Hap38 |  | Switzerland, Hungary |  |
| H36 | In this study | Hap40 |  | Switzerland |  |
| H37 | In this study | Hap41 |  | Switzerland |  |
| H38 | In this study | Hap42 |  | Hungary |  |
| H39 | In this study | Hap43 |  | Czech Republic |  |
| H40 | In this study | Hap44 |  | Czech Republic |  |
| H41 | In this study | Hap45 |  | Italy |  |
| H42 | In this study | Hap46 |  | Italy |  |
| H43 | In this study | Hap48 |  | Norway |  |
| H44 | Rey-Iglesia et al. 2017 | CGG-1-014525 | MF872239 | Spain, Liñares Cave | >38.000 |
| H44 | Rey-Iglesia et al. 2017 | CGG-1-014530 | MF872238 | Spain, Liñares Cave | >38.000 |
| H44 | Rey-Iglesia et al. 2017 | CGG-1-014536 | MF872241 | Spain, Liñares Cave | >38.000 |
| H45 | Rey-Iglesia et al. 2017 | CGG-1-014535 | MF872242 | Spain, Liñares Cave | >38.000 |
| H45 | Rey-Iglesia et al. 2017 | CGG-1-014537 | MF872243 | Spain, Liñares Cave | >38.000 |
| H46 | Rey-Iglesia et al. 2017 | CGG-1-014526 | MF872244 | Spain, Liñares Cave | >38.000 |
| H47 | Rey-Iglesia et al. 2017 | CGG-1-014538 | MF872249 | Spain, Liñares Cave | >38.000 |
