# Supplementary material for: Red deer in Iberia: Molecular ecological studies in a southern refugium and inferences on European postglacial colonization history
Source: PLoS One. 2019 Jan 8;14(1):e0210282. doi: 10.1371/journal.pone.0210282 (PMC6324796; doi:10.1371/journal.pone.0210282)
Supplement: S5 Table — Mitochondrial D-Loop similarity between the red deer haplotypes found in the present study and those reported by Stanton et al. [26]. For this comparison a 328 bp fragment size was considered, which after excluding nucleotide sites with gaps and missing data resulted in a total of 264 nucleotide sites analysed. (DOCX) [file pone.0210282.s005.docx]

**S5 Table:** Mitochondrial D-Loop similarity between the red deer haplotypes found in the present study and those reported by Stanton *et al.* [26]. For this comparison a 328 bp fragment size was considered, which after excluding nucleotide sites with gaps and missing data resulted in a total of 264 nucleotide sites analysed.

| **Haplotype** | **Reference** | **Name** | **GENBANK accession number** | **Location** | **Age**  **(years BP) Stratigraphy** |
| --- | --- | --- | --- | --- | --- |
| H01 | In this study | Hap01 |  | Iberian Peninsula | |
| H02 | In this study | Hap02 |  | Iberian Peninsula | |
| H02 | In this study | Hap37 |  | Switzerland, Czech Republic | |
| H02 | In this study | Hap39 |  | Switzerland |  |
| H03 | In this study | Hap03 |  | Iberian Peninsula | |
| H04 | In this study | Hap04 |  | Iberian Peninsula | |
| H05 | In this study | Hap05 |  | Iberian Peninsula | |
| H05 | In this study | Hap22 |  | Iberian Peninsula | |
| H06 | In this study | Hap06 |  | Iberian Peninsula | |
| H06 | In this study | Hap06´ |  | Iberian Peninsula | |
| H06 | In this study | Hap17 |  | Iberian Peninsula | |
| H07 | In this study | Hap07 |  | Iberian Peninsula | |
| H08 | In this study | Hap08 |  | Iberian Peninsula | |
| H08 | In this study | Hap16 |  | Iberian Peninsula | |
| H09 | In this study | Hap09 |  | Iberian Peninsula | |
| H10 | Stanton et al. 2016 | Hap016 | KU877708 | Norway | 7500-6500 |
| H10 | In this study | Hap10 |  | Iberian Peninsula, Italy | |
| H10 | In this study | Hap20 |  | Iberian Peninsula | |
| H10 | In this study | Hap23 |  | Iberian Peninsula | |
| H11 | Stanton et al. 2016 | Hap007 | KU877699 | Scotland (mainland) | 2200-1800 |
| H11 | In this study | Hap11 |  | Iberian Peninsula | |
| H11 | In this study | Hap31 |  | England |  |
| H12 | In this study | Hap12 |  | Iberian Peninsula | |
| H13 | In this study | Hap13 |  | Iberian Peninsula | |
| H14 | In this study | Hap14 |  | Iberian Peninsula | |
| H15 | In this study | Hap15 |  | Iberian Peninsula | |
| H16 | In this study | Hap18 |  | Iberian Peninsula | |
| H17 | In this study | Hap19 |  | Iberian Peninsula | |
| H18 | In this study | Hap21 |  | Iberian Peninsula | |
| H19 | In this study | Hap24 |  | Iberian Peninsula | |
| H20 | In this study | Hap25 |  | Iberian Peninsula | |
| H20 | In this study | Hap26 |  | Iberian Peninsula | |
| H21 | In this study | Hap27 |  | Iberian Peninsula | |
| H21 | In this study | Hap27´ |  | Iberian Peninsula | |
| H22 | In this study | Hap28 |  | Iberian Peninsula | |
| H23 | Stanton et al. 2016 | Hap010 | KU877702 | Scotland (mainland, Inner Hebrides) | 7500-6000; 7000-5500; |
| H23 | In this study | Hap29 |  | England |  |
| H24 | In this study | Hap30 |  | England |  |
| H24 | In this study | Hap35 |  | France |  |
| H25 | In this study | Hap32 |  | England, Sweden, Italy | |
| H26 | In this study | Hap33 |  | England |  |
| H27 | In this study | Hap34 |  | Switzerland, Italy | |
| H27 | In this study | Hap46 |  | Italy |  |
| H28 | In this study | Hap36 |  | France |  |
| H29 | In this study | Hap38 |  | Switzerland, Hungary | |
| H30 | In this study | Hap40 |  | Switzerland |  |
| H31 | In this study | Hap41 |  | Switzerland |  |
| H32 | In this study | Hap42 |  | Hungary |  |
| H32 | In this study | Hap45 |  | Italy |  |
| H33 | In this study | Hap43 |  | Czech Republic |  |
| H34 | In this study | Hap44 |  | Czech Republic |  |
| H35 | Stanton et al. 2016 | Hap012 | KU877704 | Scotland (Orkney) | 5000 |
| H35 | In this study | Hap47 |  | Norway |  |
| H36 | In this study | Hap48 |  | Norway |  |
| H37 | Stanton et al. 2016 | Hap008 | KU877700 | Scotland (Orkney) | 5200-4300 |
| H38 | Stanton et al. 2016 | Hap009 | KU877701 | Scotland (Outer Hebrides) | 1300-1100; 5500-4500 |
| H39 | Stanton et al. 2016 | Hap011 | KU877703 | Scotland (Outer Hebrides, Orkney) | 2000; 2500; 1000-700; 1300-1100; 5500-4500 |
| H39 | Stanton et al. 2016 | Hap013 | KU877705 | Scotland (Orkney) | 2700-2400 |
| H40 | Stanton et al. 2016 | Hap014 | KU877706 | Scotland (Outer Hebrides) | 2500 |
| H41 | Stanton et al. 2016 | Hap015 | KU877707 | Scotland (Outer Hebrides) | 2500 |
| H42 | Stanton et al. 2016 | Hap017 | KU877709 | Scotland (Outer Hebrides) | 1300-1100 |
| H43 | Stanton et al. 2016 | Hap018 | KU877710 | Scotland (Outer Hebrides) | 1000-700 |
| H44 | Stanton et al. 2016 | Hap019 | KU877711 | Scotland (Outer Hebrides) | 1300-1100 |
| H45 | Stanton et al. 2016 | Hap020 | KU877712 | Scotland (Outer Hebrides) | 1000-700 |
