# Supplementary material for: Red deer in Iberia: Molecular ecological studies in a southern refugium and inferences on European postglacial colonization history
Source: PLoS One. 2019 Jan 8;14(1):e0210282. doi: 10.1371/journal.pone.0210282 (PMC6324796; doi:10.1371/journal.pone.0210282)
Supplement: S6 Table — Ancient DNA mitochondrial D-loop sequences used for the inference of within-species evolutionary rate. (DOCX) [file pone.0210282.s006.docx]

**S6 Table:** Ancient DNA mitochondrial D-loop sequences used for the inference of within-species evolutionary rate.

| **Reference study** | **Accession number** | **Location** | **Element** | **Reference for AMS 14C** | **Radiocarbon lab number** | **Age (uncal. BP)/ Stratigraphy** | **Calibrated (Cal y BP)** |
| --- | --- | --- | --- | --- | --- | --- | --- |
| Meiri *et al.* 2013 | KF133907 | Spain, El Mirón Cave | Bone | Meiri *et al.* 2013 | OxA22089 | 14,930±70 | 18264 |
| Meiri *et al.* 2013 | KF133915 | Serbia, Rudna Glava S7 | Antler | Borić 2009 | OxA14623 | 7198±36 | 8002 |
| Meiri *et al.* 2013 | KF133918 | England, Hyena Den, Wookey Hole | Bone | Hedges *et al.* 1996 | OxA5700 | 11,320±120 | 13203 |
| Meiri *et al.* 2013 | KF133930 | Belgium, Trou Al'Wesse Couche 15 | Mandible | Meiri *et al.* 2013 | OxA22098 | 40,200±1300 | 44252 |
| Meiri *et al.* 2013 | KF133932 | Urals | Bone | Meiri *et al.* 2013 | OxA20922 | 44,650±650 | 47867 |
