# Supplementary material for: Red deer in Iberia: Molecular ecological studies in a southern refugium and inferences on European postglacial colonization history
Source: PLoS One. 2019 Jan 8;14(1):e0210282. doi: 10.1371/journal.pone.0210282 (PMC6324796; doi:10.1371/journal.pone.0210282)
Supplement: S7 Table — Bayesian MCMC analysis performed to estimate the evolutionary rate of the D-loop mitochondrial fragment studied, using calibrated fossil ages of red deer from Europe (see S6 Table). Results of independent runs were combined using TRACER, version 1.5 [87], the final result was given based on average Log10 of the Bayes factor between models. (DOCX) [file pone.0210282.s007.docx]

**S7 Table:** Bayesian MCMC analysis performed to estimate the evolutionary rate of the D-loop mitochondrial fragment studied, using calibrated fossil ages of red deer from Europe (see S6 Table). Results of independent runs were combined using TRACER, version 1.5 [87], the final result was given based on average Log_10_ of the Bayes factor between models.

| **Model** | **Ln P (model\|data)** | **S.E.** | **Uncorrelated lognormal-distributed relaxed clock - RUN 1** | **Uncorrelated lognormal-distributed relaxed clock - RUN 2** | **Strict molecular clock - RUN 1** | **Strict molecular clock - RUN 2** |
| --- | --- | --- | --- | --- | --- | --- |
| **Uncorrelated lognormal-distributed relaxed clock - RUN 1** | -2 074.6 | 1.45 | - | -0,60 | 3.81 | 1.44 |
| **Uncorrelated lognormal-distributed relaxed clock - RUN 2** | -2 073.2 | 1.44 | 0.60 | - | 4.40 | 2.04 |
| **Strict molecular clock - RUN 1** | -2 083.3 | 1.39 | -3. 81 | -4.40 | - | -2.37 |
| **Strict molecular clock - RUN 2** | -2 077.9 | 1.27 | -1.44 | -2.04 | 2.37 | - |
