# Supplementary material for: Red deer in Iberia: Molecular ecological studies in a southern refugium and inferences on European postglacial colonization history
Source: PLoS One. 2019 Jan 8;14(1):e0210282. doi: 10.1371/journal.pone.0210282 (PMC6324796; doi:10.1371/journal.pone.0210282)
Supplement: S8 Table — The bioclimatic variables used to study the climatic requirements of Cervus elaphus distribution throughout western Europe and North Africa. A ‘quarter’ refers to a fraction of the year (i.e. three months). The same variables are available for present (1950–2000), Mid-Holocene (6 ky BP), Last Glacial Maximum (22 ky BP) and Last Interglacial period (120 ky BP). (DOCX) [file pone.0210282.s008.docx]

**S8 Table:** The bioclimatic variables used to study the climatic requirements of *Cervus elaphus* distribution throughout western Europe and North Africa. A ‘quarter’ refers to a fraction of the year (i.e. three months). The same variables are available for present (1950-2000), Mid-Holocene (6 ky BP), Last Glacial Maximum (22 ky BP) and Last Interglacial period (120 ky BP).

| **Codes** | **Description (units)** |
| --- | --- |
| BIO1 | Annual mean temperature |
| BIO2 | Mean Diurnal Range (mean of monthly (max temp - min temp)) |
| BIO3 | Isothermality (mean diurnal range /BIO7) (*100) |
| BIO4 | Temperature Seasonality (standard deviation*100) |
| BIO5 | Max temperature of Warmest Month |
| BIO6 | Min temperature of Coldest Month |
| BIO7 | Temperature Annual Range (BIO5-BIO6) |
| BIO8 | Mean Temperature of Wettest Quarter |
| BIO9 | Mean Temperature of Driest Quarter |
| BIO10 | Mean temperature of Warmest Quarter |
| BIO11 | Mean temperature of Coldest Quarter |
| BIO12 | Annual Precipitation (mm) |
| BIO13 | Precipitation of Wettest Month (mm) |
| BIO14 | Precipitation of Driest Month (mm) |
| BIO15 | Precipitation Seasonality (coefficient of variation) |
| BIO16 | Precipitation of Wettest Quarter (mm) |
| BIO17 | Precipitation of Driest Quarter (mm) |
| BIO18 | Precipitation of Warmest Quarter |
| BIO19 | Precipitation of Coldest Quarter |
