# Supplementary material for: Red deer in Iberia: Molecular ecological studies in a southern refugium and inferences on European postglacial colonization history
Source: PLoS One. 2019 Jan 8;14(1):e0210282. doi: 10.1371/journal.pone.0210282 (PMC6324796; doi:10.1371/journal.pone.0210282)
Supplement: S9 Table — Results of the generalized linear model (GLM) model developed for the current distribution of Cervus elaphus in western Europe and North Africa. Predictors are listed following the order of inclusion in a stepwise procedure (the first one on top). The coefficient and its standard error (SE) and z-value test statistic values with significance levels are shown. (DOCX) [file pone.0210282.s009.docx]

**S9 Table:** Results of the generalized linear model (GLM) model developed for the current distribution of *Cervus elaphus* in western Europe and North Africa. Predictors are listed following the order of inclusion in a stepwise procedure (the first one on top). The coefficient and its standard error (SE) and z-value test statistic values with significance levels are shown.

| **Variables** | **Coefficient** | **SE** | **z-value** | ***P*** |
| --- | --- | --- | --- | --- |
| **Intercept** | -1.38 | 0.481 | -2.365 | * |
| **BIO14** | 0.029 | 0.003 | 9.124 | *** |
| **BIO8** | 0.011 | 0.001 | 8.763 | *** |
| **BIO4** | 0.003E-01 | 0.006E-02 | -6.347 | *** |
| **BIO2** | 0.019 | 0.003 | 5.120 | *** |
| **BIO1** | -0.005 | 0.001 | -2.825 | ** |

* < 0.05; ** < 0.01; *** <0.001
