# Supplementary material for: Red deer in Iberia: Molecular ecological studies in a southern refugium and inferences on European postglacial colonization history
Source: PLoS One. 2019 Jan 8;14(1):e0210282. doi: 10.1371/journal.pone.0210282 (PMC6324796; doi:10.1371/journal.pone.0210282)
Supplement: S10 Table — Results of the generalized additive model (GAM), generalized boosting model (GBM), classification tree analysis (CTA), artificial neural network (ANN), flexible discriminant analysis (FDA) and, in addition, an ensemble of their forecasts, developed on the current distribution of Cervus elaphus in western Europe and North Africa. (DOCX) [file pone.0210282.s010.docx]

**S10 Table:** Results of the generalized additive model (GAM), generalized boosting model (GBM), classification tree analysis (CTA), artificial neural network (ANN), flexible discriminant analysis (FDA) and, in addition, an ensemble of their forecasts, developed on the current distribution of *Cervus elaphus* in western Europe and North Africa.

| **Technique/ parameter** | **KAPPA** | **TSS** | **AUC** |
| --- | --- | --- | --- |
| GAM | 0.564 | 0.573 | 0.862 |
| **GBM** | **0.614** | **0.621** | **0.892** |
| **CTA** | **0.655** | **0.655** | **0.880** |
| ANN | 0.539 | 0.560 | 0.851 |
| SRE | 0.287 | 0.299 | 0.649 |
| FDA | 0.408 | 0.409 | 0.758 |
| **Ensemble of forecasts** | **0.649** | **0.655** | **0.900** |
