# Supplementary material for: Red deer in Iberia: Molecular ecological studies in a southern refugium and inferences on European postglacial colonization history
Source: PLoS One. 2019 Jan 8;14(1):e0210282. doi: 10.1371/journal.pone.0210282 (PMC6324796; doi:10.1371/journal.pone.0210282)
Supplement: S11 Table — Population codes are described as in Fig 1 of the main manuscript. (DOCX) [file pone.0210282.s011.docx]

**Table S11**: Pairwise *F_ST_* values for both microsatellite (above diagonal) and mitochondrial (below diagonal) datasets for the red deer populations studied. Population codes are described as in **Fig. 1** of the main manuscript.

| Population | ASR | CTR | HUR | BER | PMC | BUR | CFR | PNA | SLR | PNB | PNM | MT1 | MT2 | PNC | MT3 | MT4 | MT5 | MT6 | QMS | MT7 | SM1 | SM2 | SM3 | SM4 | SM5 | SM6 | MBR | PNP | PND | PNS | EN | FR | SW | SE | CZ | HU | IT | NO |
| --- | --- | --- | --- | --- | --- | --- | --- | --- | --- | --- | --- | --- | --- | --- | --- | --- | --- | --- | --- | --- | --- | --- | --- | --- | --- | --- | --- | --- | --- | --- | --- | --- | --- | --- | --- | --- | --- | --- |
| ASR | - | **0.02** | **0.07** | **0.04** | 0.03 | **0.08** | **0.13** | **0.03** | **0.02** | **0.08** | **0.06** | **0.03** | **0.03** | **0.03** | **0.03** | **0.03** | **0.02** | **0.02** | **0.02** | **0.03** | **0.02** | **0.02** | **0.02** | **0.02** | **0.02** | **0.05** | **0.05** | **0.02** | **0.10** | **0.04** | **0.17** | **0.10** | *0.10* | **0.20** | **0.09** | **0.13** | **0.08** | **0.13** |
| CTR | 0.05 | - | **0.07** | **0.05** | **0.03** | **0.08** | **0.12** | **0.05** | **0.05** | **0.12** | **0.08** | **0.04** | **0.03** | **0.05** | **0.04** | **0.03** | **0.04** | **0.03** | **0.03** | **0.03** | **0.04** | **0.03** | **0.03** | **0.04** | **0.03** | **0.06** | **0.07** | **0.05** | **0.12** | **0.07** | **0.19** | **0.12** | *0.12* | **0.20** | **0.11** | **0.14** | **0.09** | **0.15** |
| HUR | 0.22 | 0.26 | - | 0.09 | 0.08 | 0.12 | **0.19** | **0.08** | **0.08** | 0.17 | 0.14 | 0.08 | **0.08** | **0.06** | **0.04** | **0.07** | 0.05 | **0.06** | **0.05** | **0.05** | **0.10** | 0.08 | 0.08 | **0.08** | **0.08** | 0.11 | **0.12** | 0.08 | **0.17** | 0.12 | 0.25 | **0.13** | *0.13* | 0.27 | 0.15 | 0.15 | 0.11 | 0.18 |
| BER | 0.15 | **0.27** | 0.38 | - | **0.05** | 0.11 | **0.18** | **0.08** | **0.08** | **0.12** | **0.11** | **0.07** | **0.06** | **0.05** | **0.05** | **0.05** | **0.05** | **0.04** | **0.04** | **0.04** | **0.06** | **0.06** | **0.08** | **0.05** | **0.07** | **0.08** | **0.08** | **0.07** | **0.15** | **0.10** | **0.22** | **0.12** | *0.13* | **0.27** | **0.12** | **0.16** | **0.11** | **0.17** |
| PMC | 0.11 | 0.19 | 0.01 | 0.33 | - | 0.07 | **0.11** | **0.04** | **0.05** | **0.12** | **0.10** | **0.04** | **0.03** | **0.04** | **0.04** | **0.04** | **0.04** | **0.03** | **0.04** | **0.05** | 0.01 | 0.01 | 0.02 | 0.03 | 0.02 | **0.07** | **0.08** | **0.05** | **0.15** | **0.07** | **0.20** | **0.14** | *0.11* | 0.24 | 0.12 | 0.15 | 0.08 | 0.16 |
| BUR | 0.17 | 0.22 | -0.09 | 0.31 | -0.04 | - | **0.18** | **0.06** | 0.11 | 0.16 | 0.15 | 0.08 | **0.06** | **0.06** | **0.06** | 0.04 | 0.05 | 0.05 | **0.04** | **0.05** | **0.10** | 0.11 | **0.08** | **0.06** | 0.08 | 0.10 | 0.15 | 0.08 | **0.19** | 0.14 | **0.24** | **0.16** | *0.14* | 0.31 | 0.16 | 0.18 | 0.11 | 0.19 |
| CFR | **0.17** | **0.19** | **0.44** | **0.38** | **0.37** | **0.43** | - | **0.14** | **0.14** | **0.19** | **0.19** | **0.13** | **0.12** | **0.15** | **0.14** | **0.14** | **0.15** | **0.15** | **0.14** | **0.14** | **0.10** | **0.10** | **0.11** | **0.13** | **0.11** | **0.16** | **0.14** | **0.14** | **0.23** | **0.17** | **0.27** | **0.20** | *0.17* | **0.27** | **0.19** | **0.20** | **0.16** | **0.23** |
| PNA | 0.12 | **0.15** | 0.05 | **0.23** | 0.07 | 0.04 | **0.25** | - | **0.03** | **0.12** | **0.10** | **0.03** | **0.04** | **0.03** | **0.03** | **0.03** | **0.02** | **0.02** | **0.02** | **0.03** | **0.06** | **0.06** | **0.04** | **0.06** | **0.06** | **0.07** | **0.07** | **0.05** | **0.14** | **0.08** | **0.19** | **0.14** | *0.12* | **0.20** | **0.13** | **0.15** | **0.09** | **0.16** |
| SLR | 0.14 | **0.27** | 0.29 | 0.03 | 0.25 | 0.23 | **0.41** | **0.22** | - | **0.10** | **0.10** | **0.04** | **0.05** | **0.04** | **0.04** | **0.04** | **0.03** | **0.03** | **0.03** | **0.04** | **0.05** | **0.05** | **0.04** | **0.05** | **0.05** | **0.08** | **0.03** | **0.03** | **0.12** | **0.04** | **0.17** | **0.13** | *0.11* | **0.19** | **0.10** | **0.14** | **0.09** | 0.15 |
| PNB | **0.49** | **0.50** | **0.79** | **0.59** | **0.69** | **0.77** | **0.57** | **0.52** | **0.60** | - | **0.15** | **0.12** | **0.12** | **0.09** | **0.11** | **0.10** | **0.09** | **0.10** | **0.09** | **0.10** | **0.11** | **0.10** | **0.11** | **0.10** | **0.10** | **0.12** | **0.13** | **0.11** | **0.18** | **0.14** | **0.25** | **0.16** | *0.14* | **0.26** | **0.12** | **0.17** | **0.12** | **0.17** |
| PNM | **0.51** | **0.34** | **0.93** | **0.69** | **0.81** | **0.93** | **0.63** | **0.60** | **0.69** | **0.92** | - | **0.10** | **0.08** | **0.10** | **0.10** | **0.09** | **0.09** | **0.08** | **0.08** | **0.09** | **0.11** | **0.10** | **0.09** | **0.10** | **0.10** | **0.11** | **0.12** | **0.10** | **0.18** | **0.12** | **0.25** | **0.17** | *0.18* | 0.27 | **0.16** | **0.20** | **0.15** | **0.22** |
| MT1 | **0.23** | **0.26** | **0.41** | **0.29** | **0.36** | 0.35 | **0.35** | **0.26** | **0.33** | **0.56** | **0.66** | - | 0.01 | **0.03** | **0.02** | **0.02** | **0.02** | **0.01** | **0.02** | **0.03** | **0.06** | **0.06** | **0.06** | **0.05** | **0.05** | **0.07** | **0.07** | **0.05** | **0.11** | **0.08** | **0.19** | **0.12** | *0.11* | 0.21 | **0.11** | **0.16** | **0.08** | 0.16 |
| MT2 | **0.37** | **0.39** | **0.58** | **0.42** | **0.52** | 0.54 | **0.47** | **0.38** | **0.47** | **0.68** | **0.76** | 0.06 | - | **0.03** | **0.02** | **0.02** | **0.03** | **0.02** | **0.02** | **0.02** | **0.05** | **0.05** | **0.04** | **0.04** | **0.05** | **0.07** | **0.07** | **0.05** | **0.10** | **0.07** | **0.19** | **0.12** | *0.10* | **0.20** | **0.10** | **0.13** | **0.07** | **0.14** |
| PNC | **0.26** | **0.29** | 0.40 | **0.25** | **0.36** | 0.34 | **0.39** | **0.18** | **0.35** | **0.59** | **0.68** | 0.15 | 0.21 | - | 0.01 | **0.01** | **0.01** | **0.01** | **0.01** | **0.02** | **0.06** | **0.05** | **0.05** | **0.04** | **0.05** | **0.07** | **0.08** | **0.04** | **0.13** | **0.09** | **0.21** | **0.11** | *0.10* | **0.22** | **0.10** | **0.13** | **0.08** | 0.13 |
| MT3 | **0.21** | **0.24** | 0.25 | **0.26** | 0.22 | 0.19 | **0.38** | 0.13 | **0.28** | **0.56** | **0.64** | 0.08 | 0.11 | 0.09 | - | **0.02** | 0.01 | **0.01** | **0.02** | **0.01** | **0.06** | **0.06** | **0.04** | **0.05** | **0.05** | **0.07** | **0.06** | **0.03** | **0.11** | **0.07** | **0.19** | **0.10** | *0.09* | **0.20** | **0.10** | **0.13** | **0.07** | **0.12** |
| MT4 | **0.18** | **0.22** | 0.11 | **0.24** | 0.10 | 0.05 | **0.37** | 0.06 | **0.23** | **0.54** | **0.62** | **0.26** | **0.37** | **0.19** | 0.12 | - | 0.01 | 0,00 | 0.01 | **0.01** | **0.05** | **0.05** | **0.04** | **0.03** | **0.05** | **0.06** | **0.08** | **0.04** | **0.12** | **0.08** | **0.17** | **0.10** | *0.09* | **0.21** | **0.10** | **0.13** | **0.07** | **0.12** |
| MT5 | **0.17** | **0.21** | 0.03 | **0.26** | 0.05 | -0.02 | **0.38** | 0.05 | **0.22** | **0.57** | **0.66** | **0.30** | **0.43** | **0.26** | 0.16 | 0.01 | - | 0,00 | 0,00 | **0.01** | **0.05** | **0.05** | **0.04** | **0.04** | **0.05** | **0.06** | **0.07** | **0.03** | **0.11** | **0.08** | **0.18** | **0.11** | *0.09* | **0.20** | **0.09** | **0.12** | **0.07** | **0.12** |
| MT6 | **0.24** | **0.28** | -0.02 | **0.35** | 0.04 | -0.04 | **0.46** | 0.08 | **0.28** | **0.66** | **0.75** | **0.38** | **0.50** | **0.34** | 0.20 | 0.03 | 0,00 | - | 0,00 | **0.01** | **0.04** | **0.05** | **0.04** | **0.04** | **0.04** | **0.05** | **0.06** | **0.04** | **0.12** | **0.07** | **0.18** | **0.11** | *0.10* | **0.19** | **0.10** | **0.14** | **0.08** | **0.14** |
| QMS | **0.27** | **0.31** | -0.03 | **0.39** | 0.04 | -0.05 | **0.48** | 0.10 | **0.31** | **0.66** | **0.73** | **0.42** | **0.52** | **0.38** | **0.22** | 0.08 | 0.04 | -0.02 | - | **0.01** | **0.05** | **0.05** | **0.04** | **0.03** | **0.04** | **0.06** | **0.07** | **0.04** | **0.11** | **0.07** | **0.18** | **0.11** | *0.10* | **0.20** | **0.10** | **0.13** | **0.08** | **0.13** |
| MT7 | **0.20** | **0.23** | 0.28 | **0.21** | **0.24** | 0.22 | **0.33** | **0.13** | **0.27** | **0.48** | **0.54** | 0.14 | **0.20** | 0.05 | 0.06 | 0.06 | **0.14** | **0.20** | **0.25** | - | **0.05** | **0.05** | **0.04** | **0.04** | **0.05** | **0.06** | **0.07** | **0.05** | **0.11** | **0.08** | **0.19** | **0.11** | *0.11* | **0.21** | **0.10** | **0.14** | **0.08** | **0.14** |
| SM1 | **0.20** | **0.26** | **0.43** | **0.31** | **0.33** | 0.32 | **0.33** | **0.30** | **0.34** | **0.56** | **0.66** | **0.30** | **0.44** | **0.34** | **0.32** | **0.32** | **0.33** | **0.42** | **0.46** | **0.28** | - | 0.01 | 0.02 | 0.02 | 0.01 | **0.06** | **0.07** | **0.05** | **0.13** | **0.06** | **0.19** | **0.13** | *0.12* | **0.23** | **0.11** | **0.16** | **0.10** | **0.15** |
| SM2 | 0.17 | 0.19 | 0.49 | **0.40** | 0.40 | **0.48** | -0.02 | **0.26** | **0.43** | **0.66** | **0.76** | **0.36** | **0.51** | **0.41** | **0.39** | **0.39** | **0.40** | **0.50** | **0.52** | **0.34** | **0.34** | - | **0.02** | **0.03** | 0.01 | **0.06** | **0.06** | **0.05** | **0.14** | **0.06** | **0.20** | **0.13** | *0.12* | 0.23 | **0.11** | **0.14** | **0.09** | **0.14** |
| SM3 | 0.09 | **0.17** | **0.40** | **0.30** | **0.27** | 0.33 | **0.20** | **0.26** | **0.32** | **0.54** | **0.64** | **0.21** | **0.41** | **0.31** | **0.30** | **0.30** | **0.31** | **0.40** | **0.43** | **0.26** | 0.17 | 0.19 | - | 0.02 | 0.01 | **0.05** | **0.07** | **0.04** | **0.11** | **0.05** | **0.19** | **0.12** | *0.10* | 0.22 | **0.10** | **0.14** | **0.07** | 0.13 |
| SM4 | **0.17** | **0.21** | **0.37** | **0.28** | **0.29** | 0.23 | **0.27** | **0.25** | **0.31** | **0.51** | **0.59** | **0.22** | **0.35** | **0.28** | **0.25** | **0.27** | **0.28** | **0.37** | **0.40** | **0.23** | 0.02 | **0.28** | 0.14 | - | 0.01 | **0.03** | **0.06** | **0.03** | **0.09** | **0.04** | **0.17** | **0.11** | *0.11* | **0.21** | **0.10** | **0.13** | **0.08** | **0.12** |
| SM5 | 0.11 | **0.21** | **0.49** | **0.37** | 0.33 | 0.43 | 0.22 | **0.31** | **0.40** | **0.62** | **0.72** | **0.31** | **0.48** | **0.38** | **0.36** | **0.36** | **0.37** | **0.46** | **0.49** | **0.32** | **0.26** | 0.22 | 0.02 | **0.22** | - | **0.06** | **0.07** | **0.04** | **0.12** | **0.05** | **0.19** | **0.13** | *0.11* | **0.22** | **0.10** | **0.15** | **0.08** | **0.14** |
| SM6 | **0.43** | **0.45** | **0.7** | **0.52** | **0.61** | 0.56 | **0.52** | **0.47** | **0.54** | **0.77** | **0.87** | **0.49** | **0.62** | **0.52** | **0.50** | **0.48** | **0.51** | **0.60** | **0.62** | **0.43** | 0.29 | **0.59** | **0.44** | 0.14 | **0.55** | - | **0.08** | **0.06** | **0.11** | **0.07** | **0.22** | **0.14** | *0.12* | **0.20** | **0.12** | **0.14** | **0.11** | 0.17 |
| MBR | **0.27** | **0.39** | **0.61** | 0.09 | **0.53** | 0.56 | **0.47** | **0.41** | 0.14 | **0.72** | **0.83** | **0.42** | **0.56** | **0.46** | **0.43** | **0.42** | **0.44** | **0.54** | **0.57** | **0.38** | **0.42** | **0.52** | **0.41** | **0.38** | **0.49** | **0.65** | - | **0.03** | **0.14** | **0.03** | **0.19** | **0.15** | *0.12* | **0.22** | **0.12** | **0.17** | **0.10** | 0.16 |
| PNP | -0.01 | 0.10 | 0.21 | 0.08 | 0.14 | 0.16 | 0.15 | 0.10 | 0.10 | **0.47** | **0.56** | **0.19** | **0.34** | **0.22** | **0.18** | **0.16** | **0.16** | **0.24** | **0.27** | **0.16** | **0.17** | 0.15 | 0.10 | **0.14** | 0.14 | **0.40** | 0.18 | - | **0.10** | **0.03** | **0.17** | **0.12** | *0.11* | **0.22** | **0.10** | **0.13** | **0.08** | **0.12** |
| PND | **0.51** | **0.52** | **0.78** | **0.58** | **0.70** | **0.76** | **0.58** | **0.54** | **0.61** | **0.82** | **0.89** | **0.57** | **0.68** | **0.60** | **0.57** | **0.55** | **0.58** | **0.67** | **0.67** | **0.49** | **0.58** | **0.67** | **0.56** | **0.52** | **0.64** | **0.77** | **0.73** | **0.49** | - | **0.10** | **0.26** | **0.17** | *0.15* | **0.27** | **0.15** | **0.18** | **0.13** | **0.19** |
| PNS | **0.30** | **0.33** | **0.50** | **0.34** | **0.44** | 0.44 | **0.42** | **0.35** | **0.39** | **0.64** | **0.75** | **0.35** | **0.49** | **0.39** | **0.37** | **0.36** | **0.38** | **0.47** | **0.51** | **0.32** | **0.37** | **0.45** | **0.34** | **0.31** | **0.41** | **0.57** | **0.47** | **0.26** | **0.43** | - | **0.19** | **0.17** | *0.13* | 0.23 | **0.13** | **0.16** | **0.10** | **0.17** |
| EN | **0.21** | **0.24** | **0.39** | **0.28** | **0.34** | 0.31 | **0.34** | **0.26** | **0.32** | **0.56** | **0.68** | **0.25** | **0.41** | **0.30** | **0.28** | **0.28** | **0.29** | **0.39** | **0.43** | **0.24** | **0.28** | **0.35** | **0.25** | **0.22** | **0.32** | **0.48** | **0.40** | **0.17** | **0.58** | **0.32** | - | **0.20** | *0.18* | **0.30** | **0.14** | **0.18** | **0.13** | **0.23** |
| FR | **0.52** | **0.53** | **0.79** | **0.61** | **0.70** | **0.77** | **0.58** | **0.54** | **0.62** | **0.82** | **0.90** | **0.58** | **0.69** | **0.61** | **0.58** | **0.57** | **0.59** | **0.67** | **0.68** | **0.50** | **0.59** | **0.68** | **0.57** | **0.53** | **0.64** | **0.77** | **0.73** | **0.50** | **0.81** | **0.66** | **0.59** | - | *0.09* | **0.25** | **0.08** | **0.12** | **0.07** | **0.11** |
| SW | **0.20** | **0.23** | **0.36** | **0.25** | **0.31** | 0.29 | **0.32** | **0.24** | **0.29** | **0.50** | **0.60** | **0.23** | **0.37** | **0.28** | **0.26** | **0.26** | **0.27** | **0.36** | **0.40** | **0.23** | **0.26** | **0.33** | **0.23** | **0.21** | **0.29** | **0.44** | **0.37** | **0.16** | **0.52** | **0.30** | **0.20** | **0.53** | - | *0.18* | *0.08* | *0.08* | *0.02* | *0.11* |
| SE | **0.44** | **0.46** | 0.82 | **0.53** | **0.64** | 0.78 | **0.54** | **0.48** | **0.55** | **0.86** | **1,00** | **0.49** | **0.65** | **0.54** | **0.51** | **0.50** | **0.52** | **0.63** | **0.65** | **0.45** | **0.51** | **0.62** | **0.48** | **0.45** | **0.57** | **0.78** | **0.70** | **0.40** | **0.84** | **0.59** | 0.20 | **0.85** | **0.44** | - | 0.21 | 0.23 | 0.16 | 0.25 |
| CZ | **0.23** | **0.26** | **0.43** | **0.31** | **0.37** | 0.35 | **0.36** | **0.28** | **0.34** | **0.62** | **0.76** | **0.27** | **0.44** | **0.32** | **0.30** | **0.30** | **0.31** | **0.42** | **0.47** | **0.26** | **0.30** | **0.39** | **0.27** | **0.24** | **0.35** | **0.53** | **0.44** | **0.19** | **0.64** | **0.35** | **0.24** | **0.65** | 0.20 | 0.54 | - | 0.06 | 0.03 | 0.08 |
| HU | **0.43** | **0.45** | 0.75 | **0.52** | **0.62** | 0.71 | **0.53** | **0.47** | **0.54** | **0.82** | **0.94** | **0.49** | **0.63** | **0.52** | **0.50** | **0.49** | **0.51** | **0.61** | **0.63** | **0.43** | **0.50** | **0.60** | **0.47** | **0.44** | **0.56** | **0.74** | **0.67** | **0.39** | **0.81** | **0.58** | **0.47** | **0.81** | 0.28 | 0.87 | **0.53** | - | 0.05 | 0.12 |
| IT | 0.17 | 0.19 | 0.35 | **0.24** | **0.31** | 0.27 | 0.27 | 0.21 | **0.29** | **0.57** | **0.71** | 0.22 | **0.39** | **0.27** | **0.25** | 0.25 | **0.26** | **0.36** | **0.42** | 0.21 | 0.24 | 0.29 | 0.21 | 0.18 | **0.29** | **0.47** | **0.38** | 0.12 | **0.58** | 0.29 | 0.14 | **0.6** | 0.05 | 0.41 | **0.20** | **0.47** | - | 0.05 |
| NO | 0.27 | 0.30 | 0.54 | 0.36 | 0.44 | 0.45 | **0.41** | 0.33 | 0.40 | **0.73** | **0.89** | 0.32 | **0.51** | 0.37 | 0.35 | 0.35 | 0.36 | 0.48 | **0.52** | 0.30 | 0.35 | 0.45 | 0.31 | 0.28 | 0.40 | **0.62** | 0.52 | 0.23 | **0.73** | 0.41 | 0.29 | **0.74** | 0.26 | 0.70 | 0.32 | 0.66 | 0.24 | - |

Significant statistical p-values (*P* < 0.001, after Bonferroni correction) are depicted in bold, while the values for which was not possible to determine the statistical significance are shown in italics.
