# Supplementary material for: Red deer in Iberia: Molecular ecological studies in a southern refugium and inferences on European postglacial colonization history
Source: PLoS One. 2019 Jan 8;14(1):e0210282. doi: 10.1371/journal.pone.0210282 (PMC6324796; doi:10.1371/journal.pone.0210282)
Supplement: S12 Table — Number of individuals excluded from the Bayesian STRUCTURE analysis at the Iberian level, using microsatellite data. Exclusion was based on the individuals that had a membership proportion (qi) from the Iberian cluster lower than 95% in the main STRUCTURE analysis (all European populations) and in the European mtDNA phylogeographic analysis. Two individuals fulfilled both requisites. (DOCX) [file pone.0210282.s012.docx]

**S12 Table**: Number of red deer individuals excluded from the Bayesian STRUCTURE analysis at the Iberian level, using microsatellite data. Exclusion was based on the individuals that had a membership proportion (qi) from the Iberian cluster lower than 95% in the main STRUCTURE analysis (all European populations) and in the European mitochondrial (mtDNA) phylogeographic analysis. In the latter, the haplotype 11 and haplotypy 49 were excluded since they might have evolved outside the Iberian Peninsula. Two individuals fulfilled both requisites.

| **Population** | **STRUCTURE analysis** | **mtDNA phylogeographic analysis** |  |
| --- | --- | --- | --- |
|  | **IB <95% qi** | **Haplotype 11 and 49** |  |
| **ASR** | 2 | 6 |  |
| **CTR** | 0 | 0 |  |
| **HUR** | 0 | 0 |  |
| **BR** | 1 | 11 |  |
| **PNMSC** | 0 | 0 |  |
| **BUR** | 0 | 0 |  |
| **CPFGR** | 0 | 0 |  |
| **PNAT** | 0 | 0 |  |
| **SLR** | 3 | 14 |  |
| **PNBT** | 2 | 0 |  |
| **PNM** | 2 | 0 |  |
| **MT1** | 0 | 0 |  |
| **MT2** | 0 | 0 |  |
| **PNC** | 0 | 0 |  |
| **MT3** | 1 | 0 |  |
| **MT4** | 1 | 0 |  |
| **MT5** | 0 | 0 |  |
| **MT6** | 1 | 0 |  |
| **QM** | 0 | 0 |  |
| **MT7** | 3 | 0 |  |
| **SM1** | 0 | 1 |  |
| **SM2** | 0 | 0 |  |
| **SM3** | 0 | 0 |  |
| **SM4** | 4 | 0 |  |
| **SM5** | 0 | 0 |  |
| **SM6** | 1 | 0 |  |
| **MBR** | 0 | 11 |  |
| **PNSAPA** | 2 | 6 |  |
| **PND** | 2 | 0 |  |
| **PNSG** | 0 | 1 |  |
| **TOTAL** | 25 | 50 | 73 |
