# Supplementary material for: Red deer in Iberia: Molecular ecological studies in a southern refugium and inferences on European postglacial colonization history
Source: PLoS One. 2019 Jan 8;14(1):e0210282. doi: 10.1371/journal.pone.0210282 (PMC6324796; doi:10.1371/journal.pone.0210282)
Supplement: S13 Table — Posterior parameter estimates (median and 95% confidence intervals) for the best-supported scenario calculated using 1% of simulated datasets closest to the observed values. Simulations and approximate Bayesian computation analyses were performed including only nuclear makers and considering both nuclear and mitochondrial markers. Relative median absolute errors (RMAE) based on 500 pseudo-observed datasets are also given for each parameter. N1, N2, etc.—Effective population size of extant populations; t1—estimated date of lineage splitting in years (assuming a generation time of 8.33 years [104]); μ, mutation rate. Population codes are described as in Fig 1 of the main manuscript. (DOCX) [file pone.0210282.s013.docx]

**S13 Table**. Posterior parameter estimates (median and 95% confidence intervals) for the best-supported scenario calculated using 1% of simulated datasets closest to the observed values. Simulations and approximate Bayesian computation analyses were performed including only nuclear makers and considering both nuclear and mitochondrial markers. Relative median absolute errors (RMAE) based on 500 pseudo-observed datasets are also given for each parameter. N1, N2, etc. - Effective population size of extant populations; t1 - estimated date of lineage splitting in years (assuming a generation time of 8.33 years [104]); **μ**, mutation rate. Population codes are described as in Fig 1 of the main manuscript.

| **Parameters** | **European** | | | |  | **Parameters** | **Iberian** | | | |
| --- | --- | --- | --- | --- | --- | --- | --- | --- | --- | --- |
|  | **Median** | **q [2.5]** | **q[97.5]** | **RMAE** |  |  | **Median** | **q [2.5]** | **q[97.5]** | **RMAE** |
| **N1 (IB)** | 4800 | 1680 | 9120 | 0.207 |  | **N1 (MTR)** | 5030 | 2100 | 7790 | 0.230 |
| **N2 (FR)** | 4380 | 1420 | 8980 | 0.218 |  | **N2 (PNB)** | 1310 | 271 | 6790 | 0.241 |
| **N3 (EN)** | 1660 | 498 | 6530 | 0.215 |  | **N3 (SMR)** | 4130 | 1080 | 9030 | 0.249 |
| **N4 (NO)** | 1870 | 523 | 7480 | 0.271 |  | **N4 (CFR)** | 1090 | 244 | 5730 | 0.247 |
| **N5 (SE)** | 789 | 233 | 4510 | 0.270 |  |  |  |  |  |  |
| **N6 (CZ)** | 8750 | 5860 | 9900 | 0.137 |  |  |  |  |  |  |
| **N7 (HU)** | 5070 | 2500 | 7910 | 0.172 |  |  |  |  |  |  |
| **t1** | 11495 | 5131 | 21075 | 0.207 |  | **t1** | 5022 | 1641 | 10079 | 0.414 |
| **μ** | 1.19E-4 | 1.00E-4 | 2.19E-4 | 0.200 |  | **μ** | 1.14E-4 | 1.00E-4 | 2.50E-4 | 0.098 |
