# Supplementary material for: Red deer in Iberia: Molecular ecological studies in a southern refugium and inferences on European postglacial colonization history
Source: PLoS One. 2019 Jan 8;14(1):e0210282. doi: 10.1371/journal.pone.0210282 (PMC6324796; doi:10.1371/journal.pone.0210282)
Supplement: S1 Fig — Plots showing the results for both the Bayesian clustering analyses conducted in STRUCTURE software (see Figs 3A and 4A and S4 Fig) and the highest ΔK value obtained following Evanno et al. [59] procedures. (DOCX) [file pone.0210282.s014.docx]

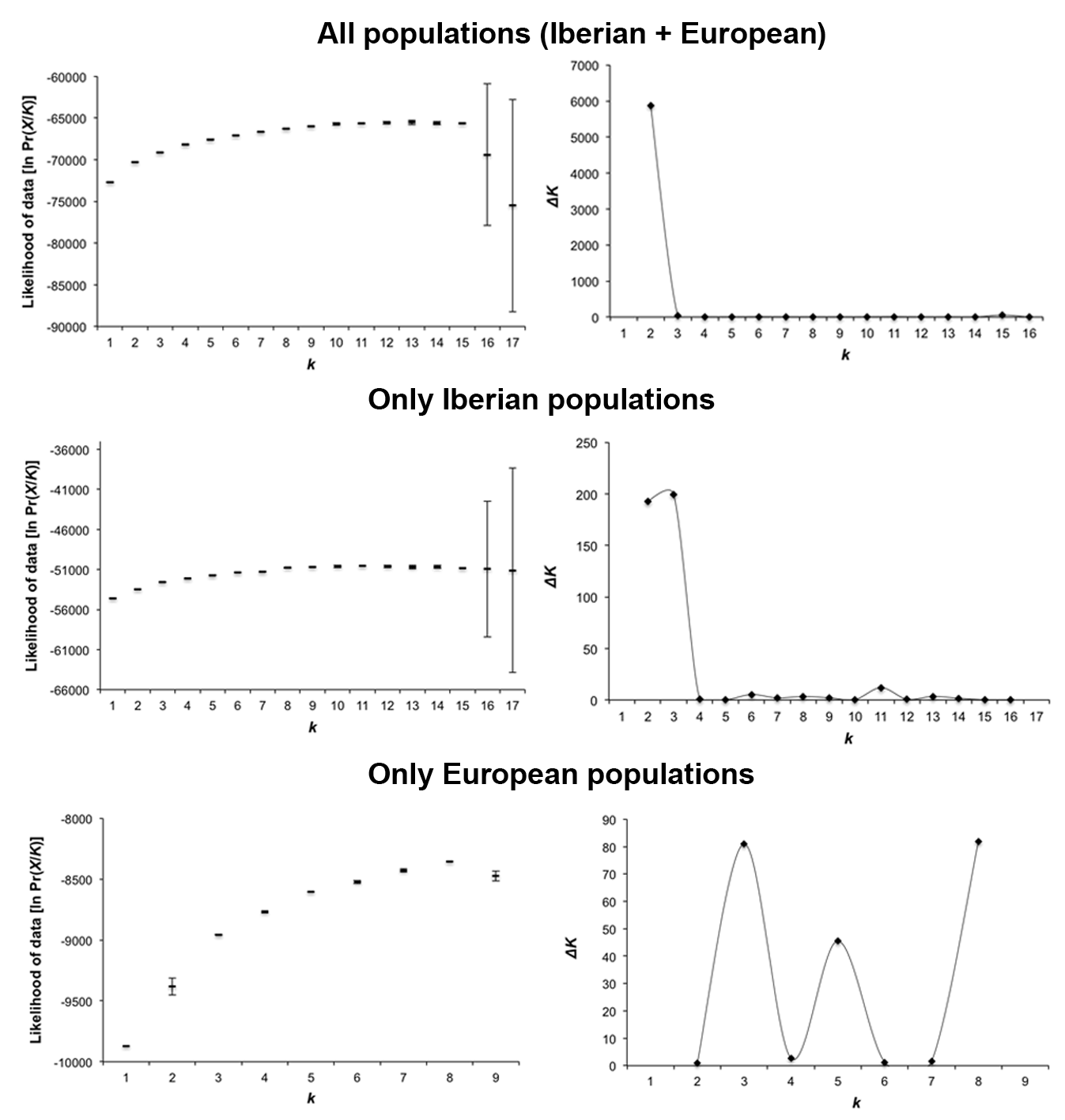


**S1 Fig.** Plots showing the results for both the Bayesian clustering analyses conducted in STRUCTURE software (see Figs 3a, 4a and S4 Fig) and the highest Δ*K* value obtained following Evanno *et al.* [56] procedures.
