# Supplementary material for: Red deer in Iberia: Molecular ecological studies in a southern refugium and inferences on European postglacial colonization history
Source: PLoS One. 2019 Jan 8;14(1):e0210282. doi: 10.1371/journal.pone.0210282 (PMC6324796; doi:10.1371/journal.pone.0210282)
Supplement: S2 Fig — Neighbor-joining trees representing the genetic differentiation among populations measured as pairwise FST for both microsatellite (left) and mtDNA (right) datasets. Population codes are described in Fig 1 of the main manuscript. (DOCX) [file pone.0210282.s015.docx]

**S2 Fig.** Neighbor-joining trees representing the genetic differentiation among populations measured as pairwise *F_ST_* for both microsatellite (left) and mtDNA (right) datasets. Population codes are described in Fig 1 of the main manuscript.
