# Supplementary material for: Red deer in Iberia: Molecular ecological studies in a southern refugium and inferences on European postglacial colonization history
Source: PLoS One. 2019 Jan 8;14(1):e0210282. doi: 10.1371/journal.pone.0210282 (PMC6324796; doi:10.1371/journal.pone.0210282)
Supplement: S3 Fig — Plots showing the relationship between genetic distance [pairwise FST/(1-FST)] and geographic distance (log km) between the Iberian red deer populations quantified for both microsatellites and mitochondrial datasets (isolation-by-distance). (DOCX) [file pone.0210282.s016.docx]

**
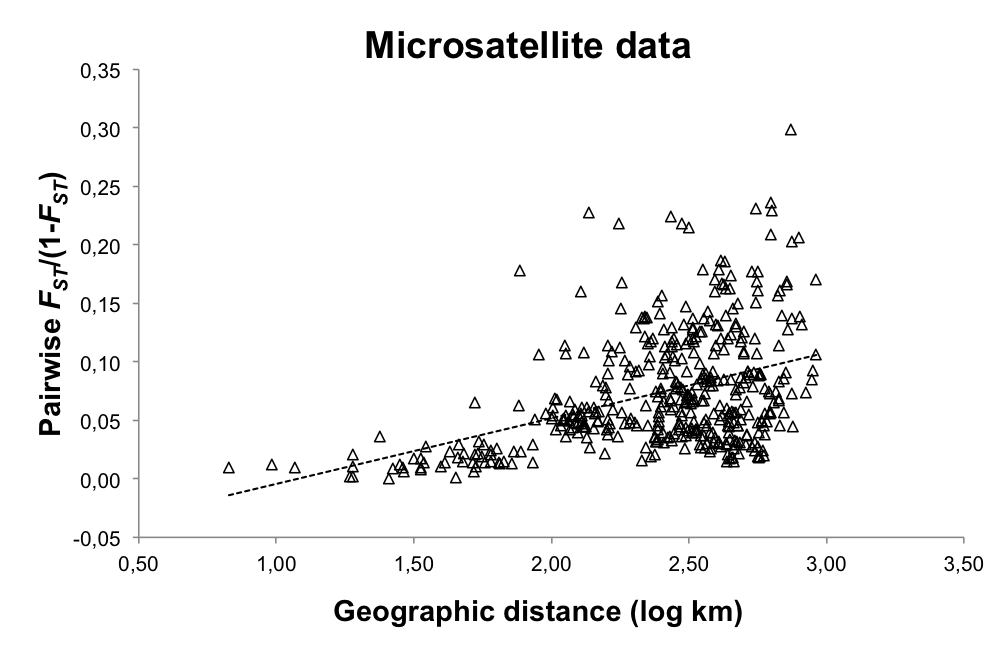

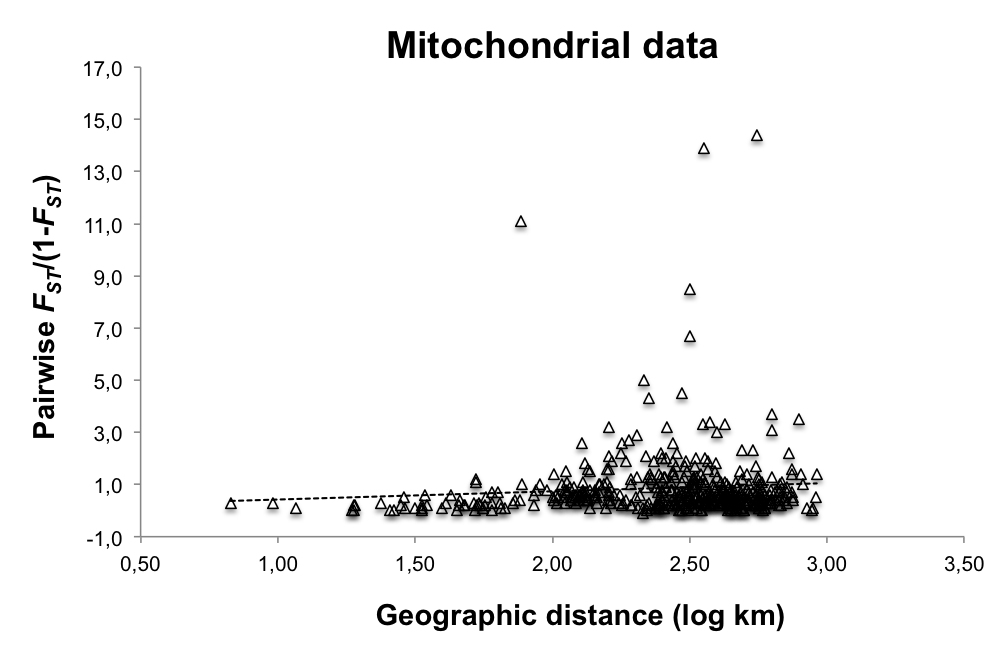
**

**S3 Fig.** Plots showing the relationship between the genetic distance [pairwise *F_ST_*/(1-*F_ST_*)] and geographic distance (log km) between the Iberian red deer populations quantified for both microsatellites and mitochondrial datasets (isolation-by-distance).
