# Supplementary material for: Red deer in Iberia: Molecular ecological studies in a southern refugium and inferences on European postglacial colonization history
Source: PLoS One. 2019 Jan 8;14(1):e0210282. doi: 10.1371/journal.pone.0210282 (PMC6324796; doi:10.1371/journal.pone.0210282)
Supplement: S4 Fig — Bayesian clustering analyses performed in STRUCTURE [56] on microsatellite data from the central and north European red deer populations, considering the best ΔK values obtained following Evanno et al. [59] procedures (see also S1 Fig). Proportional membership to each cluster is indicated for K = 3 and K = 8. Population codes are described as in Fig 1 of the main manuscript. (DOCX) [file pone.0210282.s017.docx]

**
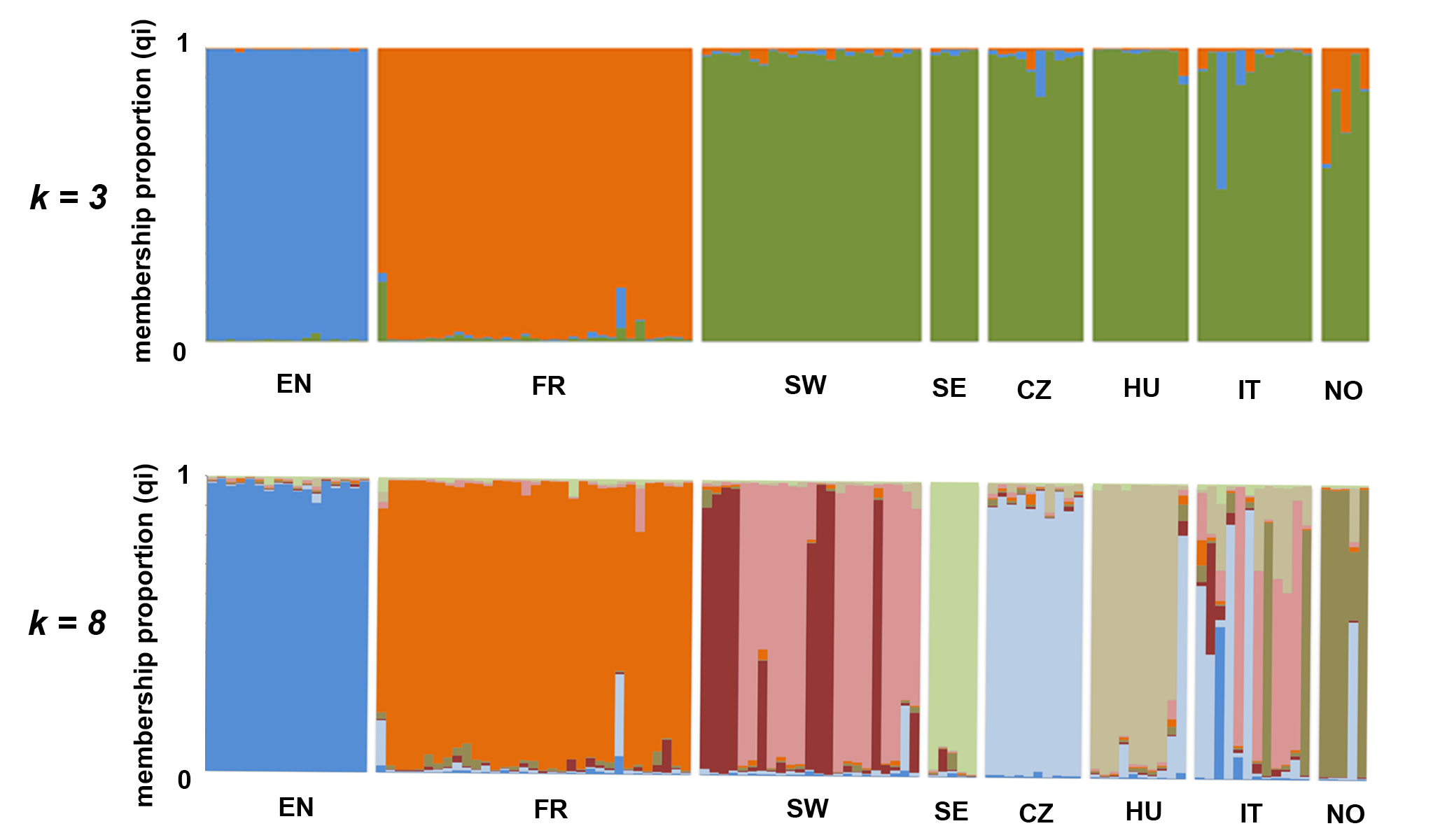
**

**S4 Fig**. Bayesian clustering analyses performed in STRUCTURE [53] on microsatellite data from the central and north European red deer populations, considering the best Δ*K* values obtained following Evanno *et al.* [56] procedures (see also S1 Fig). Proportional membership to each cluster is indicated for *K* = 3 and *K* = 8. Population codes are described as in Fig 1 of the main manuscript.
