# Supplementary material for: Red deer in Iberia: Molecular ecological studies in a southern refugium and inferences on European postglacial colonization history
Source: PLoS One. 2019 Jan 8;14(1):e0210282. doi: 10.1371/journal.pone.0210282 (PMC6324796; doi:10.1371/journal.pone.0210282)
Supplement: S5 Fig — Median joining network showing the evolutionary relationship of D-loop haplotypes found in this study (670 bp fragment size) for the red deer in Europe. Numbers within circles correspond to haplotype name described in the main manuscript for the 329 bp haplotypes (see details in S1 Table). A bar on each solid line represents a mutational step; small black circles show undetected/extinct intermediate haplotype states; color codes within the circles are depicted for Western European lineage (haplogroup A) and represent the country where the haplotypes were found. The Iberian sub-lineages are grouped by colored shading following Fig 6. (DOCX) [file pone.0210282.s018.docx]

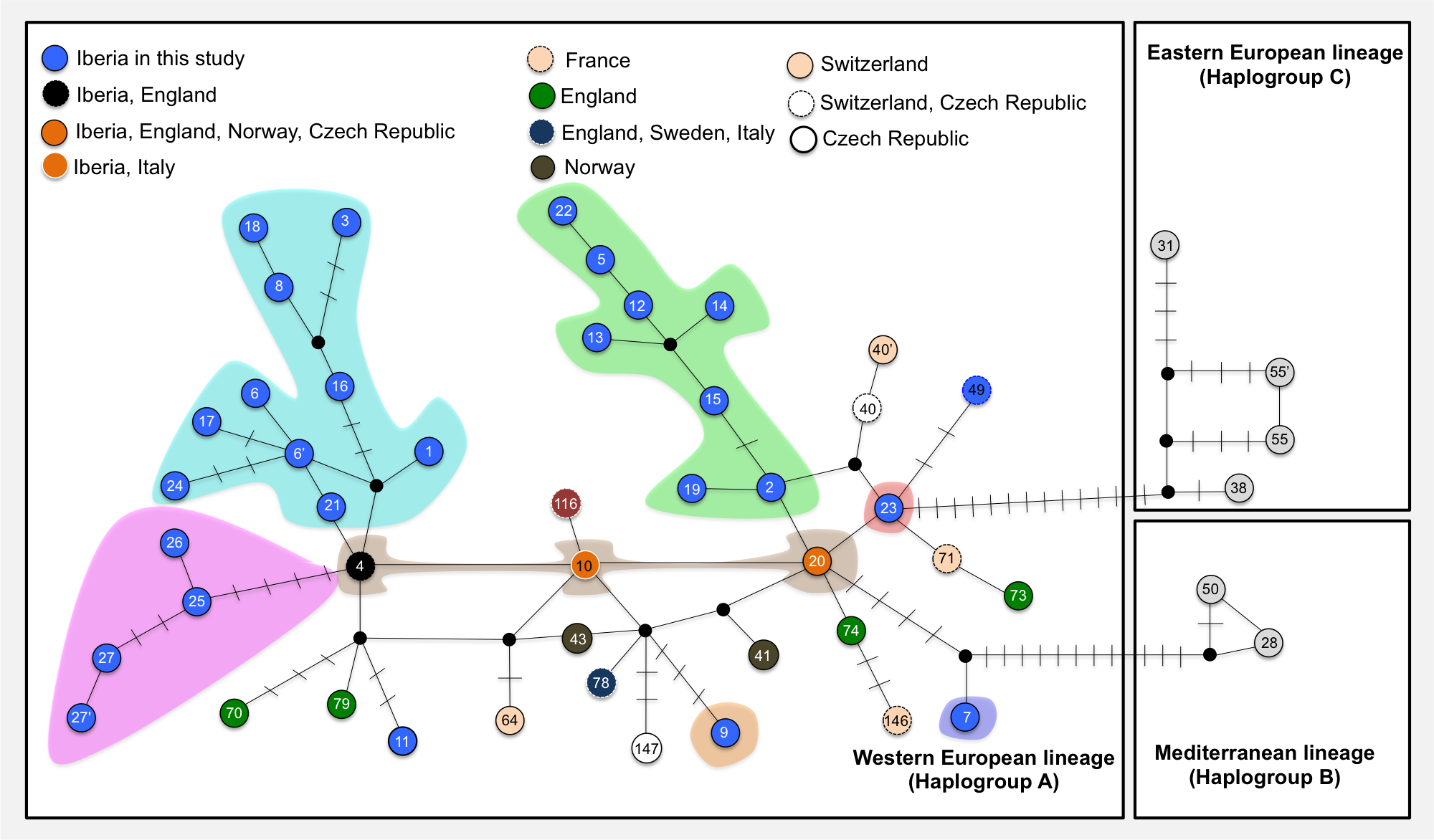


**S5 Fig.** Median joining network showing the evolutionary relationship of D-loop haplotypes found in this study (670 bp fragment size) for the red deer in Europe. Numbers within circles correspond to haplotype name described in the main manuscript for the 329bp haplotypes (see details in S1 Table). A bar in each solid line represents a mutational step; small black circles show undetected/extinct intermediate haplotype states; color codes within the circles are depicted for western European lineage (haplogroup A) and represent the country where the haplotypes were found. The Iberian sub-lineages are grouped by colored shading following **Fig 6**.
