# Supplementary material for: Red deer in Iberia: Molecular ecological studies in a southern refugium and inferences on European postglacial colonization history
Source: PLoS One. 2019 Jan 8;14(1):e0210282. doi: 10.1371/journal.pone.0210282 (PMC6324796; doi:10.1371/journal.pone.0210282)
Supplement: S6 Fig — Calibration plot showing the relationship between the predicted probability for red deer occurrence according to its climatic niche and the observed frequency of the species in the validation dataset. Open symbols indicate bins with < 15 localities, in which the frequency observed should be considered with caution [139]. (DOCX) [file pone.0210282.s019.docx]

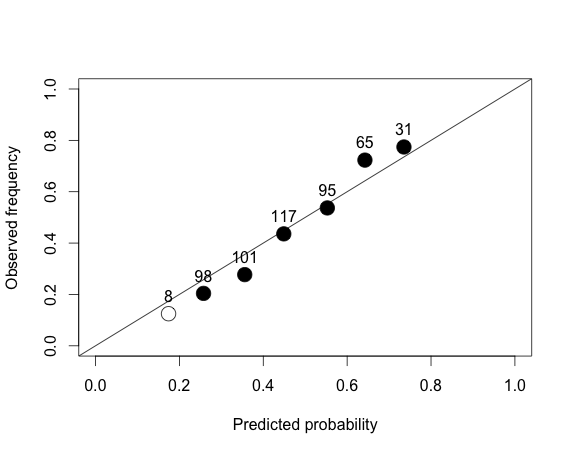


**S6 Fig.** Calibration plot showing the relationship between the predicted probability for red deer occurrence according to its climatic niche and the observed frequency of the species in the validation dataset. Open symbols indicate bins with < 15 localities, in which the frequency observed should be considered with caution (Jovani & Tella 2006).
