# Supplementary material for: Red deer in Iberia: Molecular ecological studies in a southern refugium and inferences on European postglacial colonization history
Source: PLoS One. 2019 Jan 8;14(1):e0210282. doi: 10.1371/journal.pone.0210282 (PMC6324796; doi:10.1371/journal.pone.0210282)
Supplement: S7 Fig — Climatic suitability for the occurrence of Cervus elaphus in western Europe and North Africa during the Mid-Holocene (6 ky BP), Last Glacial Maximum (LGM, 22 ky BP) and interglacial period (120 ky BP) represented for the generalized boosting model (GBM), classification tree analysis (CTA) and the ensemble of their forecasts, according to the statistical model shown in S10 Table. (DOCX) [file pone.0210282.s020.docx]

**
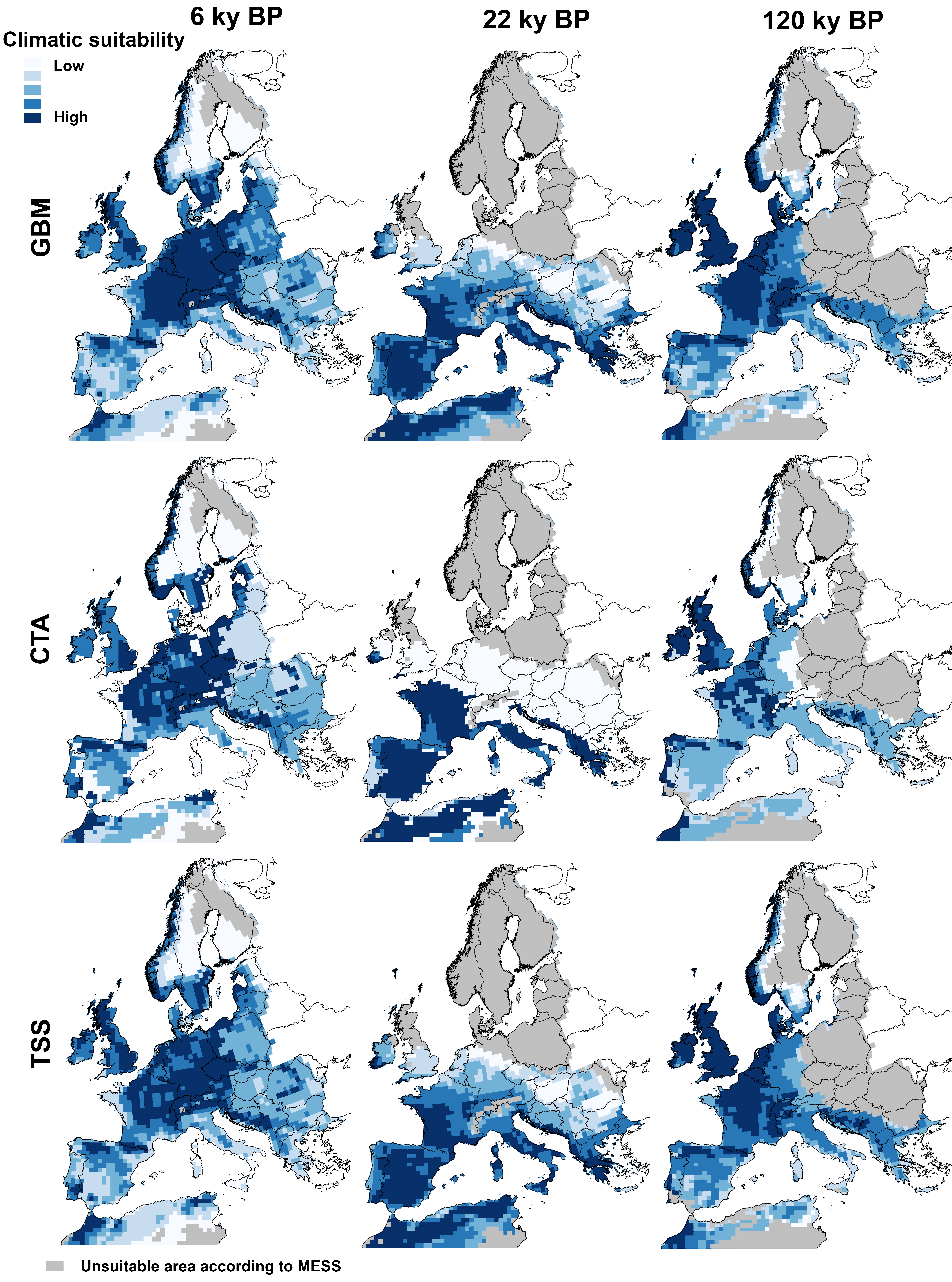
**

**S7 Fig.** Climatic suitability for *Cervus elaphus* occurrence in western Europe and North Africa during the Mid-Holocene (6 kyBP), Last Glacial Maximum (LGM, 22 kyBP) and interglacial period (120 kyBP) represented for the generalized boosting model (GBM), classification tree analysis (CTA) and the ensemble of their forecasts (TSS), according to the statistical model shown in S10 Table.
