# Supplementary material for: Red deer in Iberia: Molecular ecological studies in a southern refugium and inferences on European postglacial colonization history
Source: PLoS One. 2019 Jan 8;14(1):e0210282. doi: 10.1371/journal.pone.0210282 (PMC6324796; doi:10.1371/journal.pone.0210282)
Supplement: S8 Fig — Map showing the geographic distribution of the red deer fossil records dated within the period of Last Glacial Maximum (Fig 9) and the climatic suitability for occurrence of red deer at 22 kyBP (Fig 8), predicted according to the climatic niche for the species determined by a generalized linear model (GLM) model. (DOCX) [file pone.0210282.s021.docx]

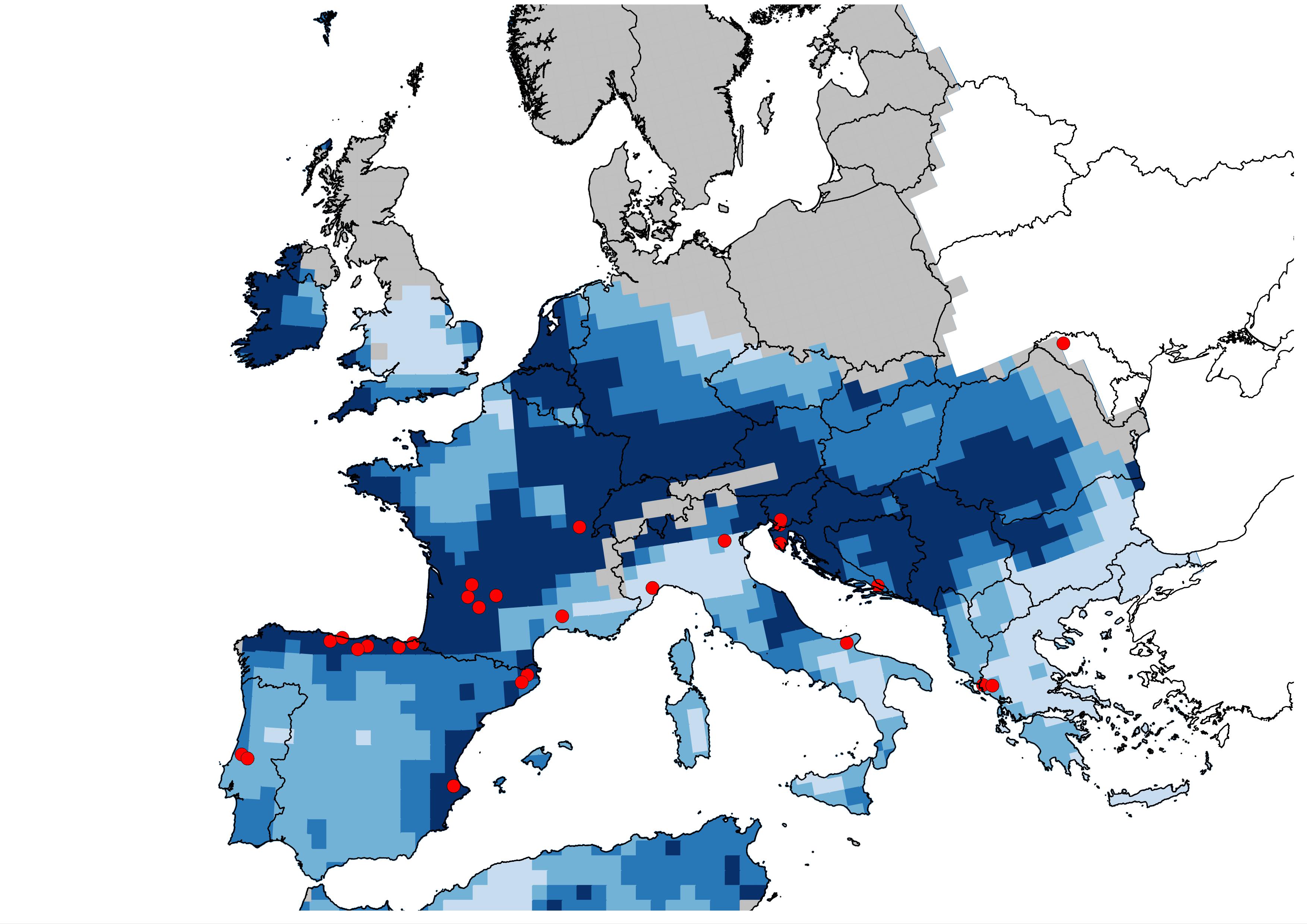


**S8 Fig**. Map showing the geographic distribution of the red deer fossil records dated within the period of Last Glacial Maximum (**Fig 9**) and the climatic suitability for occurrence of red deer at 22 kyBP (**Fig 8**), predicted according to the climatic niche for the species determined by a GLM model.
